# Supplementary material for: Measuring clients’ experiences with antenatal care before or after childbirth: it matters
Source: PeerJ. 2018 Nov 27;6:e5851. doi: 10.7717/peerj.5851 (PMC6266936; doi:10.7717/peerj.5851)
Supplement: Supplemental Information 2 [file peerj-06-5851-s002.docx]

# CODE – antenatal experiences before and after birth

Date: May 21th, 2018

Author: M. Scheerhagen

Table 2.

The association between the late antenatal experiences measured during pregnancy and after childbirth, expressed as having a negative experience, below the median score and mean score (n=462).

### Negative score

**Total.

FREQUENCIES RQ_EvT_Neg_B RQ_EvT_Neg_B_Re.

CROSSTABS TABLES = RQ_EvT_Neg_B BY RQ_EvT_Neg_B_Re

/CELLS COUNT ROW EXPECTED COLUMN RESID

/STATISTICS KAPPA.

*personal.

FREQUENCIES RQ_PS_Neg_B RQ_PS_Neg_B_Re.

CROSSTABS TABLES = RQ_PS_Neg_B BY RQ_PS_Neg_B_Re

/CELLS COUNT ROW EXPECTED COLUMN RESID

/STATISTICS KAPPA.

*Setting.

FREQUENCIES RQ_ST_Neg_B RQ_ST_Neg_B_Re.

CROSSTABS TABLES = RQ_ST_Neg_B BY RQ_ST_Neg_B_Re

/CELLS COUNT ROW EXPECTED COLUMN RESID

/STATISTICS KAPPA.

**respect.

FREQUENCIES RQ_R_Neg_B RQ_R_Neg_B_Re.

CROSSTABS TABLES = RQ_R_Neg_B BY RQ_R_Neg_B_Re

/CELLS COUNT ROW EXPECTED COLUMN RESID

/STATISTICS KAPPA.

*autnomie.

FREQUENCIES RQ_A_Neg_B RQ_A_Neg_B_Re.

CROSSTABS TABLES = RQ_A_Neg_B BY RQ_A_Neg_B_Re

/CELLS COUNT ROW EXPECTED COLUMN RESID

/STATISTICS KAPPA.

*privacy.

FREQUENCIES RQ_P_Neg_B RQ_P_Neg_B_Re.

CROSSTABS TABLES = RQ_P_Neg_B BY RQ_P_Neg_B_Re

/CELLS COUNT ROW EXPECTED COLUMN RESID

/STATISTICS KAPPA.

*communicatie.

FREQUENCIES RQ_C_Neg_B RQ_C_Neg_B_Re.

CROSSTABS TABLES = RQ_C_Neg_B BY RQ_C_Neg_B_Re

/CELLS COUNT ROW EXPECTED COLUMN RESID

/STATISTICS KAPPA.

*tijd tot hulp.

FREQUENCIES RQ_T_Neg_B RQ_T_Neg_B_Re.

CROSSTABS TABLES = RQ_T_Neg_B BY RQ_T_Neg_B_Re

/CELLS COUNT ROW EXPECTED COLUMN RESID

/STATISTICS KAPPA.

*sociale ondersteuning.

FREQUENCIES RQ_S_Neg_B RQ_S_Neg_B_Re.

CROSSTABS TABLES = RQ_S_Neg_B BY RQ_S_Neg_B_Re

/CELLS COUNT ROW EXPECTED COLUMN RESID

/STATISTICS KAPPA.

*faciliteiten.

FREQUENCIES RQ_F_Neg_B RQ_F_Neg_B_Re.

CROSSTABS TABLES = RQ_F_Neg_B BY RQ_F_Neg_B_Re

/CELLS COUNT ROW EXPECTED COLUMN RESID

/STATISTICS KAPPA.

*keuze en continuiteit.

FREQUENCIES RQ_K_Neg_B RQ_K_Neg_B_Re.

CROSSTABS TABLES = RQ_K_Neg_B BY RQ_K_Neg_B_Re

/CELLS COUNT ROW EXPECTED COLUMN RESID

/STATISTICS KAPPA.

*Test, 1e controles - 2e helft zwangerschap.

NUMERIC RQ_EvT_AA_N_B (F2.0).

COMPUTE RQ_EvT_AA_N_B = -999.

IF (RQ_EvT_Neg_B = RQ_EvT_Neg_B_Re) RQ_EvT_AA_N_B =1.

IF (RQ_EvT_Neg_B <> RQ_EvT_Neg_B_Re) RQ_EvT_AA_N_B =2.

VARIABLE LABELS RQ_EvT_AA_N_B 'Overeenstemming % negatief late zwangerschap (test-hertest) - overall'.

VALUE LABELS RQ_EvT_AA_N_B

1 'late zwangerschap (test-hertest)zelfde categorieen'

2 'late zwangerschap (test-hertest)niet in zelfde categorieen'.

FREQUENCIES RQ_EvT_AA_N_B.

NUMERIC RQ_PS_AA_N_B (F2.0).

COMPUTE RQ_PS_AA_N_B = -999.

IF (RQ_PS_Neg_B = RQ_PS_Neg_B_Re) RQ_PS_AA_N_B =1.

IF (RQ_PS_Neg_B <> RQ_PS_Neg_B_Re) RQ_PS_AA_N_B =2.

VARIABLE LABELS RQ_PS_AA_N_B 'Overeenstemming % negatief late zwangerschap (test-hertest) - personal'.

VALUE LABELS RQ_PS_AA_N_B

1 'late zwangerschap (test-hertest)zelfde categorieen'

2 'late zwangerschap (test-hertest)niet in zelfde categorieen'.

FREQUENCIES RQ_PS_AA_N_B.

NUMERIC RQ_ST_AA_N_B (F2.0).

COMPUTE RQ_ST_AA_N_B = -999.

IF (RQ_ST_Neg_B = RQ_ST_Neg_B_Re) RQ_ST_AA_N_B =1.

IF (RQ_ST_Neg_B <> RQ_ST_Neg_B_Re) RQ_ST_AA_N_B =2.

VARIABLE LABELS RQ_ST_AA_N_B 'Overeenstemming % negatief late zwangerschap (test-hertest) - setting'.

VALUE LABELS RQ_ST_AA_N_B

1 'late zwangerschap (test-hertest)zelfde categorieen'

2 'late zwangerschap (test-hertest)niet in zelfde categorieen'.

FREQUENCIES RQ_ST_AA_N_B.

NUMERIC RQ_R_AA_N_B (F2.0).

COMPUTE RQ_R_AA_N_B = -999.

IF (RQ_R_Neg_B = RQ_R_Neg_B_Re) RQ_R_AA_N_B =1.

IF (RQ_R_Neg_B <> RQ_R_Neg_B_Re) RQ_R_AA_N_B =2.

VARIABLE LABELS RQ_R_AA_N_B 'Overeenstemming % negatief late zwangerschap (test-hertest) - respect'.

VALUE LABELS RQ_R_AA_N_B

1 'late zwangerschap (test-hertest)zelfde categorieen'

2 'late zwangerschap (test-hertest)niet in zelfde categorieen'.

FREQUENCIES RQ_R_AA_N_B.

NUMERIC RQ_A_AA_N_B (F2.0).

COMPUTE RQ_A_AA_N_B = -999.

IF (RQ_A_Neg_B = RQ_A_Neg_B_Re) RQ_A_AA_N_B =1.

IF (RQ_A_Neg_B <> RQ_A_Neg_B_Re) RQ_A_AA_N_B =2.

VARIABLE LABELS RQ_A_AA_N_B 'Overeenstemming % negatief late zwangerschap (test-hertest) - autonomie'.

VALUE LABELS RQ_A_AA_N_B

1 'late zwangerschap (test-hertest)zelfde categorieen'

2 'late zwangerschap (test-hertest)niet in zelfde categorieen'.

FREQUENCIES RQ_A_AA_N_B.

NUMERIC RQ_P_AA_N_B (F2.0).

COMPUTE RQ_P_AA_N_B = -999.

IF (RQ_P_Neg_B = RQ_P_Neg_B_Re) RQ_P_AA_N_B =1.

IF (RQ_P_Neg_B <> RQ_P_Neg_B_Re) RQ_P_AA_N_B =2.

VARIABLE LABELS RQ_P_AA_N_B 'Overeenstemming % negatief late zwangerschap (test-hertest) - privacy'.

VALUE LABELS RQ_P_AA_N_B

1 'late zwangerschap (test-hertest)zelfde categorieen'

2 'late zwangerschap (test-hertest)niet in zelfde categorieen'.

FREQUENCIES RQ_P_AA_N_B.

NUMERIC RQ_C_AA_N_B (F2.0).

COMPUTE RQ_C_AA_N_B = -999.

IF (RQ_C_Neg_B = RQ_C_Neg_B_Re) RQ_C_AA_N_B =1.

IF (RQ_C_Neg_B <> RQ_C_Neg_B_Re) RQ_C_AA_N_B =2.

VARIABLE LABELS RQ_C_AA_N_B 'Overeenstemming % negatief late zwangerschap (test-hertest) - communicatie'.

VALUE LABELS RQ_C_AA_N_B

1 'late zwangerschap (test-hertest)zelfde categorieen'

2 'late zwangerschap (test-hertest)niet in zelfde categorieen'.

FREQUENCIES RQ_C_AA_N_B.

NUMERIC RQ_T_AA_N_B (F2.0).

COMPUTE RQ_T_AA_N_B = -999.

IF (RQ_T_Neg_B = RQ_T_Neg_B_Re) RQ_T_AA_N_B =1.

IF (RQ_T_Neg_B <> RQ_T_Neg_B_Re) RQ_T_AA_N_B =2.

VARIABLE LABELS RQ_T_AA_N_B 'Overeenstemming % negatief late zwangerschap (test-hertest) - tijd tot hulp'.

VALUE LABELS RQ_T_AA_N_B

1 'late zwangerschap (test-hertest)zelfde categorieen'

2 'late zwangerschap (test-hertest)niet in zelfde categorieen'.

FREQUENCIES RQ_T_AA_N_B.

NUMERIC RQ_S_AA_N_B (F2.0).

COMPUTE RQ_S_AA_N_B = -999.

IF (RQ_S_Neg_B = RQ_S_Neg_B_Re) RQ_S_AA_N_B =1.

IF (RQ_S_Neg_B <> RQ_S_Neg_B_Re) RQ_S_AA_N_B =2.

VARIABLE LABELS RQ_S_AA_N_B 'Overeenstemming % negatief late zwangerschap (test-hertest) - sociale ondersteuning'.

VALUE LABELS RQ_S_AA_N_B

1 'late zwangerschap (test-hertest)zelfde categorieen'

2 'late zwangerschap (test-hertest)niet in zelfde categorieen'.

FREQUENCIES RQ_S_AA_N_B.

NUMERIC RQ_F_AA_N_B (F2.0).

COMPUTE RQ_F_AA_N_B = -999.

IF (RQ_F_Neg_B = RQ_F_Neg_B_Re) RQ_F_AA_N_B =1.

IF (RQ_F_Neg_B <> RQ_F_Neg_B_Re) RQ_F_AA_N_B =2.

VARIABLE LABELS RQ_F_AA_N_B 'Overeenstemming % negatief late zwangerschap (test-hertest) - faciltieiten'.

VALUE LABELS RQ_F_AA_N_B

1 'late zwangerschap (test-hertest)zelfde categorieen'

2 'late zwangerschap (test-hertest)niet in zelfde categorieen'.

FREQUENCIES RQ_F_AA_N_B.

NUMERIC RQ_K_AA_N_B (F2.0).

COMPUTE RQ_K_AA_N_B = -999.

IF (RQ_K_Neg_B = RQ_K_Neg_B_Re) RQ_K_AA_N_B =1.

IF (RQ_K_Neg_B <> RQ_K_Neg_B_Re) RQ_K_AA_N_B =2.

VARIABLE LABELS RQ_K_AA_N_B 'Overeenstemming % negatief late zwangerschap (test-hertest) - Keuze en continuiteit'.

VALUE LABELS RQ_K_AA_N_B

1 'late zwangerschap (test-hertest)zelfde categorieen'

2 'late zwangerschap (test-hertest)niet in zelfde categorieen'.

FREQUENCIES RQ_K_AA_N_B.

### Median score

**Total.

FREQUENCIES RQ_EvT_MD_B RQ_EvT_MD_B_Re.

CROSSTABS TABLES = RQ_EvT_MD_B BY RQ_EvT_MD_B_Re

/CELLS COUNT ROW EXPECTED COLUMN RESID

/STATISTICS KAPPA.

*personal.

FREQUENCIES RQ_PS_MD_B RQ_PS_MD_B_Re.

CROSSTABS TABLES = RQ_PS_MD_B BY RQ_PS_MD_B_Re

/CELLS COUNT ROW EXPECTED COLUMN RESID

/STATISTICS KAPPA.

*Setting.

FREQUENCIES RQ_ST_MD_B RQ_ST_MD_B_Re.

CROSSTABS TABLES = RQ_ST_MD_B BY RQ_ST_MD_B_Re

/CELLS COUNT ROW EXPECTED COLUMN RESID

/STATISTICS KAPPA.

**respect.

FREQUENCIES RQ_R_MD_B RQ_R_MD_B_Re.

CROSSTABS TABLES = RQ_R_MD_B BY RQ_R_MD_B_Re

/CELLS COUNT ROW EXPECTED COLUMN RESID

/STATISTICS KAPPA.

*autnomie.

FREQUENCIES RQ_A_MD_B RQ_A_MD_B_Re.

CROSSTABS TABLES = RQ_A_MD_B BY RQ_A_MD_B_Re

/CELLS COUNT ROW EXPECTED COLUMN RESID

/STATISTICS KAPPA.

*privacy.

FREQUENCIES RQ_P_MD_B RQ_P_MD_B_Re.

CROSSTABS TABLES = RQ_P_MD_B BY RQ_P_MD_B_Re

/CELLS COUNT ROW EXPECTED COLUMN RESID

/STATISTICS KAPPA.

*communicatie.

FREQUENCIES RQ_C_MD_B RQ_C_MD_B_Re.

CROSSTABS TABLES = RQ_C_MD_B BY RQ_C_MD_B_Re

/CELLS COUNT ROW EXPECTED COLUMN RESID

/STATISTICS KAPPA.

*tijd tot hulp.

FREQUENCIES RQ_T_MD_B RQ_T_MD_B_Re.

CROSSTABS TABLES = RQ_T_MD_B BY RQ_T_MD_B_Re

/CELLS COUNT ROW EXPECTED COLUMN RESID

/STATISTICS KAPPA.

*sociale ondersteuning.

FREQUENCIES RQ_S_MD_B RQ_S_MD_B_Re.

CROSSTABS TABLES = RQ_S_MD_B BY RQ_S_MD_B_Re

/CELLS COUNT ROW EXPECTED COLUMN RESID

/STATISTICS KAPPA.

*faciliteiten.

FREQUENCIES RQ_F_MD_B RQ_F_MD_B_Re.

CROSSTABS TABLES = RQ_F_MD_B BY RQ_F_MD_B_Re

/CELLS COUNT ROW EXPECTED COLUMN RESID

/STATISTICS KAPPA.

*keuze en continuiteit.

FREQUENCIES RQ_K_MD_B RQ_K_MD_B_Re.

CROSSTABS TABLES = RQ_K_MD_B BY RQ_K_MD_B_Re

/CELLS COUNT ROW EXPECTED COLUMN RESID

/STATISTICS KAPPA.

*Test, 1e controles - 2e helft zwangerschap.

NUMERIC RQ_EvT_AA_MD_B (F2.0).

COMPUTE RQ_EvT_AA_MD_B = $SYSMIS.

IF (RQ_EvT_MD_B = RQ_EvT_MD_B_Re) RQ_EvT_AA_MD_B =1.

IF (RQ_EvT_MD_B <> RQ_EvT_MD_B_Re) RQ_EvT_AA_MD_B =2.

VARIABLE LABELS RQ_EvT_AA_MD_B 'Overeenstemming mediaan late zwangerschap (test-hertest)- overall'.

VALUE LABELS RQ_EvT_AA_MD_B

1 'test-hertest zelfde categorieen'

2 'test-hertest niet in zelfde categorieen'.

FREQUENCIES RQ_EvT_AA_MD_B.

NUMERIC RQ_PS_AA_MD_B (F2.0).

COMPUTE RQ_PS_AA_MD_B = $SYSMIS.

IF (RQ_PS_MD_B = RQ_PS_MD_B_Re) RQ_PS_AA_MD_B =1.

IF (RQ_PS_MD_B <> RQ_PS_MD_B_Re) RQ_PS_AA_MD_B =2.

VARIABLE LABELS RQ_PS_AA_MD_B 'Overeenstemming mediaan late zwangerschap (test-hertest)- personal'.

VALUE LABELS RQ_PS_AA_MD_B

1 'test-hertest zelfde categorieen'

2 'test-hertest niet in zelfde categorieen'.

FREQUENCIES RQ_PS_AA_MD_B.

NUMERIC RQ_ST_AA_MD_B (F2.0).

COMPUTE RQ_ST_AA_MD_B = $SYSMIS.

IF (RQ_ST_MD_B = RQ_ST_MD_B_Re) RQ_ST_AA_MD_B =1.

IF (RQ_ST_MD_B <> RQ_ST_MD_B_Re) RQ_ST_AA_MD_B =2.

VARIABLE LABELS RQ_ST_AA_MD_B 'Overeenstemming mediaan late zwangerschap (test-hertest)- setting'.

VALUE LABELS RQ_ST_AA_MD_B

1 'test-hertest zelfde categorieen'

2 'test-hertest niet in zelfde categorieen'.

FREQUENCIES RQ_ST_AA_MD_B.

NUMERIC RQ_R_AA_MD_B (F2.0).

COMPUTE RQ_R_AA_MD_B = $SYSMIS.

IF (RQ_R_MD_B = RQ_R_MD_B_Re) RQ_R_AA_MD_B =1.

IF (RQ_R_MD_B <> RQ_R_MD_B_Re) RQ_R_AA_MD_B =2.

VARIABLE LABELS RQ_R_AA_MD_B 'Overeenstemming mediaan late zwangerschap (test-hertest)- respect'.

VALUE LABELS RQ_R_AA_MD_B

1 'test-hertest zelfde categorieen'

2 'test-hertest niet in zelfde categorieen'.

FREQUENCIES RQ_R_AA_MD_B.

NUMERIC RQ_A_AA_MD_B (F2.0).

COMPUTE RQ_A_AA_MD_B = $SYSMIS.

IF (RQ_A_MD_B = RQ_A_MD_B_Re) RQ_A_AA_MD_B =1.

IF (RQ_A_MD_B <> RQ_A_MD_B_Re) RQ_A_AA_MD_B =2.

VARIABLE LABELS RQ_A_AA_MD_B 'Overeenstemming mediaan late zwangerschap (test-hertest)- autonomie'.

VALUE LABELS RQ_A_AA_MD_B

1 'test-hertest zelfde categorieen'

2 'test-hertest niet in zelfde categorieen'.

FREQUENCIES RQ_A_AA_MD_B.

NUMERIC RQ_P_AA_MD_B (F2.0).

COMPUTE RQ_P_AA_MD_B = $SYSMIS.

IF (RQ_P_MD_B = RQ_P_MD_B_Re) RQ_P_AA_MD_B =1.

IF (RQ_P_MD_B <> RQ_P_MD_B_Re) RQ_P_AA_MD_B =2.

VARIABLE LABELS RQ_P_AA_MD_B 'Overeenstemming mediaan late zwangerschap (test-hertest)- privacy'.

VALUE LABELS RQ_P_AA_MD_B

1 'test-hertest zelfde categorieen'

2 'test-hertest niet in zelfde categorieen'.

FREQUENCIES RQ_P_AA_MD_B.

NUMERIC RQ_C_AA_MD_B (F2.0).

COMPUTE RQ_C_AA_MD_B = $SYSMIS.

IF (RQ_C_MD_B = RQ_C_MD_B_Re) RQ_C_AA_MD_B =1.

IF (RQ_C_MD_B <> RQ_C_MD_B_Re) RQ_C_AA_MD_B =2.

VARIABLE LABELS RQ_C_AA_MD_B 'Overeenstemming mediaan late zwangerschap (test-hertest)- communicatie'.

VALUE LABELS RQ_C_AA_MD_B

1 'test-hertest zelfde categorieen'

2 'test-hertest niet in zelfde categorieen'.

FREQUENCIES RQ_C_AA_MD_B.

NUMERIC RQ_T_AA_MD_B (F2.0).

COMPUTE RQ_T_AA_MD_B = $SYSMIS.

IF (RQ_T_MD_B = RQ_T_MD_B_Re) RQ_T_AA_MD_B =1.

IF (RQ_T_MD_B <> RQ_T_MD_B_Re) RQ_T_AA_MD_B =2.

VARIABLE LABELS RQ_T_AA_MD_B 'Overeenstemming mediaan late zwangerschap (test-hertest)- tijd tot hulp'.

VALUE LABELS RQ_T_AA_MD_B

1 'test-hertest zelfde categorieen'

2 'test-hertest niet in zelfde categorieen'.

FREQUENCIES RQ_T_AA_MD_B.

NUMERIC RQ_S_AA_MD_B (F2.0).

COMPUTE RQ_S_AA_MD_B = $SYSMIS.

IF (RQ_S_MD_B = RQ_S_MD_B_Re) RQ_S_AA_MD_B =1.

IF (RQ_S_MD_B <> RQ_S_MD_B_Re) RQ_S_AA_MD_B =2.

VARIABLE LABELS RQ_S_AA_MD_B 'Overeenstemming mediaan late zwangerschap (test-hertest)- sociale ondersteuning'.

VALUE LABELS RQ_S_AA_MD_B

1 'test-hertest zelfde categorieen'

2 'test-hertest niet in zelfde categorieen'.

FREQUENCIES RQ_S_AA_MD_B.

NUMERIC RQ_F_AA_MD_B (F2.0).

COMPUTE RQ_F_AA_MD_B = $SYSMIS.

IF (RQ_F_MD_B = RQ_F_MD_B_Re) RQ_F_AA_MD_B =1.

IF (RQ_F_MD_B <> RQ_F_MD_B_Re) RQ_F_AA_MD_B =2.

VARIABLE LABELS RQ_F_AA_MD_B 'Overeenstemming mediaan late zwangerschap (test-hertest)- faciltieiten'.

VALUE LABELS RQ_F_AA_MD_B

1 'test-hertest zelfde categorieen'

2 'test-hertest niet in zelfde categorieen'.

FREQUENCIES RQ_F_AA_MD_B.

NUMERIC RQ_K_AA_MD_B (F2.0).

COMPUTE RQ_K_AA_MD_B = $SYSMIS.

IF (RQ_K_MD_B = RQ_K_MD_B_Re) RQ_K_AA_MD_B =1.

IF (RQ_K_MD_B <> RQ_K_MD_B_Re) RQ_K_AA_MD_B =2.

VARIABLE LABELS RQ_K_AA_MD_B 'Overeenstemming mediaan late zwangerschap (test-hertest)- Keuze en continuiteit'.

VALUE LABELS RQ_K_AA_MD_B

1 'test-hertest zelfde categorieen'

2 'test-hertest niet in zelfde categorieen'.

FREQUENCIES RQ_K_AA_MD_B.

### Mean score

*ICC

*totaal score.

RELIABILITY

/VARIABLES=RQ_EvT_B RQ_EvT_B_Re

/SCALE('ICC total') ALL

/MODEL=ALPHA

/STATISTICS=DESCRIPTIVE SCALE

/ICC=MODEL(MIXED) TYPE(ABSOLUTE) CIN=95 TESTVAL=0.

*personal score.

RELIABILITY

/VARIABLES=RQ_PS_Dom_B RQ_PS_Dom_B_Re

/SCALE('ICC persoonsdomeinen') ALL

/MODEL=ALPHA

/STATISTICS=DESCRIPTIVE SCALE

/ICC=MODEL(MIXED) TYPE(ABSOLUTE) CIN=95 TESTVAL=0.

*setting score.

RELIABILITY

/VARIABLES=RQ_ST_Dom_B RQ_ST_Dom_B_Re

/SCALE('ICC settingdomeinen') ALL

/MODEL=ALPHA

/STATISTICS=DESCRIPTIVE SCALE

/ICC=MODEL(MIXED) TYPE(ABSOLUTE) CIN=95 TESTVAL=0.

*respect.

RELIABILITY

/VARIABLES=RQ_R_Dom_B RQ_R_Dom_B_Re

/SCALE('ICC Respect') ALL

/MODEL=ALPHA

/STATISTICS=DESCRIPTIVE SCALE

/ICC=MODEL(MIXED) TYPE(ABSOLUTE) CIN=95 TESTVAL=0.

*autonomie.

RELIABILITY

/VARIABLES=RQ_A_Dom_B RQ_A_Dom_B_Re

/SCALE('ICC autonomie') ALL

/MODEL=ALPHA

/STATISTICS=DESCRIPTIVE SCALE

/ICC=MODEL(MIXED) TYPE(ABSOLUTE) CIN=95 TESTVAL=0.

*privacy.

RELIABILITY

/VARIABLES=RQ_P_Dom_B RQ_P_Dom_B_Re

/SCALE('ICC privacy') ALL

/MODEL=ALPHA

/STATISTICS=DESCRIPTIVE SCALE

/ICC=MODEL(MIXED) TYPE(ABSOLUTE) CIN=95 TESTVAL=0.

*communication.

RELIABILITY

/VARIABLES=RQ_C_Dom_B RQ_C_Dom_B_Re

/SCALE('ICC communicatie B-B_Re') ALL

/MODEL=ALPHA

/STATISTICS=DESCRIPTIVE SCALE

/ICC=MODEL(MIXED) TYPE(ABSOLUTE) CIN=95 TESTVAL=0.

*tijd tot hulp.

RELIABILITY

/VARIABLES=RQ_T_Dom_B RQ_T_Dom_B_Re

/SCALE('ICC tijd tot hulp') ALL

/MODEL=ALPHA

/STATISTICS=DESCRIPTIVE SCALE

/ICC=MODEL(MIXED) TYPE(ABSOLUTE) CIN=95 TESTVAL=0.

*Sociale ondersteuning.

RELIABILITY

/VARIABLES=RQ_S_Dom_B RQ_S_Dom_B_Re

/SCALE('ICC Sociale ondersteuning') ALL

/MODEL=ALPHA

/STATISTICS=DESCRIPTIVE SCALE

/ICC=MODEL(MIXED) TYPE(ABSOLUTE) CIN=95 TESTVAL=0.

*Faciliteiten..

RELIABILITY

/VARIABLES=RQ_F_Dom_B RQ_F_Dom_B_Re

/SCALE('ICC faciliteiten') ALL

/MODEL=ALPHA

/STATISTICS=DESCRIPTIVE SCALE

/ICC=MODEL(MIXED) TYPE(ABSOLUTE) CIN=95 TESTVAL=0.

*Keuze en continuiteit.

RELIABILITY

/VARIABLES=RQ_K_Dom_B RQ_K_Dom_B_Re

/SCALE('ICC keuze en continuiteit') ALL

/MODEL=ALPHA

/STATISTICS=DESCRIPTIVE SCALE

/ICC=MODEL(MIXED) TYPE(ABSOLUTE) CIN=95 TESTVAL=0.

DESCRIPTIVES

RQ_EvT_B

RQ_PS_Dom_B

RQ_ST_Dom_B

RQ_R_Dom_B

RQ_A_Dom_B

RQ_P_Dom_B

RQ_C_Dom_B

RQ_T_Dom_B

RQ_S_Dom_B

RQ_F_Dom_B

RQ_K_Dom_B

/STATISTICS MEAN STDDEV.

DESCRIPTIVES

RQ_EvT_B_Re

RQ_PS_Dom_B_Re

RQ_ST_Dom_B_Re

RQ_R_Dom_B_Re

RQ_A_Dom_B_Re

RQ_P_Dom_B_Re

RQ_C_Dom_B_Re

RQ_T_Dom_B_Re

RQ_S_Dom_B_Re

RQ_F_Dom_B_Re

RQ_K_Dom_B_Re

/STATISTICS MEAN STDDEV.

## Table 3.

Level of absolute agreement between the items measured during pregnancy and after childbirth (n=462).

### Negative score

* STAP 1: berekenen percentage negatief op item niveau TEST.

*respect.

NUMERIC RQ_R_Pri_B_N (F2.0).

COMPUTE RQ_R_Pri_B_N = 1.

IF (RQ_R_Pri_B_M=1 OR (RQ_R_Pri_B_M = 2 AND OV_MeE_R_A=1)) RQ_R_Pri_B_N =2.

VARIABLE LABELS RQ_R_Pri_B_N 'Negatief Rekening houden met privacy, 2e helft zws'.

VALUE LABELS RQ_R_Pri_B_N

2 'negatief'

1 'positief'.

FREQUENCIES RQ_R_Pri_B_N.

NUMERIC RQ_R_RES_B_N (F2.0).

COMPUTE RQ_R_RES_B_N = 1.

IF (RQ_R_Res_B_M=1 OR (RQ_R_Res_B_M = 2 AND OV_MeE_R_A=1)) RQ_R_Res_B_N =2.

VARIABLE LABELS RQ_R_RES_B_N 'Negatief Behandeld met respect, 2e helft zws'.

VALUE LABELS RQ_R_RES_B_N

2 'negatief'

1 'positief'.

FREQUENCIES RQ_R_RES_B_N.

NUMERIC RQ_R_Per_B_N (F2.0).

COMPUTE RQ_R_Per_B_N = 1.

IF (RQ_R_Per_B_M=1 OR (RQ_R_Per_B_M = 2 AND OV_MeE_R_A=1)) RQ_R_Per_B_N =2.

VARIABLE LABELS RQ_R_Per_B_N 'Negatief Persoonlijke aandacht, 2e helft zws'.

VALUE LABELS RQ_R_Per_B_N

2 'negatief'

1 'positief'.

FREQUENCIES RQ_R_Per_B_N.

NUMERIC RQ_R_Vri_B_N (F2.0).

COMPUTE RQ_R_Vri_B_N = 1.

IF (RQ_R_Vri_B_M=1 OR (RQ_R_Vri_B_M = 2 AND OV_MeE_R_A=1)) RQ_R_Vri_B_N =2.

VARIABLE LABELS RQ_R_Vri_B_N 'Negatief Vriendelijk behandeld, 2e helft zws'.

VALUE LABELS RQ_R_Vri_B_N

2 'negatief'

1 'positief'.

FREQUENCIES RQ_R_Vri_B_N.

NUMERIC RQ_R_Wen_B_N (F2.0).

COMPUTE RQ_R_Wen_B_N = 1.

IF (RQ_R_Wen_B_M=1 OR (RQ_R_Wen_B_M = 2 AND OV_MeE_R_A=1)) RQ_R_Wen_B_N =2.

VARIABLE LABELS RQ_R_Wen_B_N 'Negatief Rekening houden met wensen en behoeften, 2e helft zws'.

VALUE LABELS RQ_R_Wen_B_N

2 'negatief'

1 'positief'.

FREQUENCIES RQ_R_Wen_B_N.

NUMERIC RQ_R_Ver_B_N (F2.0).

COMPUTE RQ_R_Ver_B_N = 1.

IF (RQ_R_Ver_B_M=1 OR (RQ_R_Ver_B_M = 2 AND OV_MeE_R_A=1)) RQ_R_Ver_B_N =2.

VARIABLE LABELS RQ_R_Ver_B_N 'Negatief Alles vertellen aan zorgverlener, 2e helft zws'.

VALUE LABELS RQ_R_Ver_B_N

2 'negatief'

1 'positief'.

FREQUENCIES RQ_R_Ver_B_N.

*autonomie.

NUMERIC RQ_A_Wei_B_N (F2.0).

COMPUTE RQ_A_Wei_B_N = 1.

IF (RQ_A_Wei_B_M=1 OR (RQ_A_Wei_B_M = 2 AND OV_MeE_A_A=1)) RQ_A_Wei_B_N =2.

VARIABLE LABELS RQ_A_Wei_B_N 'Negatief Weigeren behandeling, 2e helft zws'.

VALUE LABELS RQ_A_Wei_B_N

2 'negatief'

1 'positief'.

FREQUENCIES RQ_A_Wei_B_N.

NUMERIC RQ_A_Mee_B_N (F2.0).

COMPUTE RQ_A_Mee_B_N = 1.

IF (RQ_A_Mee_B_M=1 OR (RQ_A_Mee_B_M = 2 AND OV_MeE_A_A=1)) RQ_A_Mee_B_N =2.

VARIABLE LABELS RQ_A_Mee_B_N 'Negatief Meebeslissen, 2e helft zws'.

VALUE LABELS RQ_A_Mee_B_N

2 'negatief'

1 'positief'.

FREQUENCIES RQ_A_Mee_B_N.

NUMERIC RQ_A_SvD_B_N (F2.0).

COMPUTE RQ_A_SvD_B_N = 1.

IF (RQ_A_SvD_M_Cat3=1 OR (RQ_A_SvD_M_Cat3 = 2 AND OV_MeE_A_A=1)) RQ_A_SvD_B_N =2.

VARIABLE LABELS RQ_A_SvD_B_N 'Negatief Syndroom van Down, 2e helft zws'.

VALUE LABELS RQ_A_SvD_B_N

2 'negatief'

1 'positief'.

FREQUENCIES RQ_A_SvD_B_N.

NUMERIC RQ_A_GBP_B_N (F2.0).

COMPUTE RQ_A_GBP_B_N = 1.

IF (RQ_A_Gbp_B_M_Cat5=1 OR (RQ_A_Gbp_B_M_Cat5 = 2 AND OV_MeE_A_A=1)) RQ_A_GBP_B_N =2.

VARIABLE LABELS RQ_A_GBP_B_N 'Negatief Geboorteplan, 2e helft zws'.

VALUE LABELS RQ_A_GBP_B_N

2 'negatief'

1 'positief'.

FREQUENCIES RQ_A_GBP_B_N.

*privacy.

NUMERIC RQ_P_Med_B_N (F2.0).

COMPUTE RQ_P_Med_B_N = 1.

IF (RQ_P_Med_B_M=1 OR (RQ_P_Med_B_M = 1 AND OV_MeE_P_A=1)) RQ_P_Med_B_N =2.

VARIABLE LABELS RQ_P_Med_B_N 'Negatief Medische dossier, 2e helft zws, hertest'.

VALUE LABELS RQ_P_Med_B_N

2 'negatief'

1 'positief'.

FREQUENCIES RQ_P_Med_B_N.

NUMERIC RQ_P_Mln_B_N (F2.0).

COMPUTE RQ_P_Mln_B_N = 1.

IF (RQ_P_Mln_B_M=1 OR (RQ_P_Mln_B_M = 2 AND OV_MeE_P_A=1)) RQ_P_Mln_B_N =2.

VARIABLE LABELS RQ_P_Mln_B_N 'Negatief Meeluisteren, 2e helft zws, hertest'.

VALUE LABELS RQ_P_Mln_B_N

2 'negatief'

1 'positief'.

FREQUENCIES RQ_P_Mln_B_N.

*communicatie.

NUMERIC RQ_C_Ant_B_N (F2.0).

COMPUTE RQ_C_Ant_B_N = 1.

IF (RQ_C_Ant_B_M=1 OR (RQ_C_Ant_B_M = 2 AND OV_MeE_C_A=1)) RQ_C_Ant_B_N =2.

VARIABLE LABELS RQ_C_Ant_B_N 'Negatief Antwoord op vragen, 2e helft zws'.

VALUE LABELS RQ_C_Ant_B_N

2 'negatief'

1 'positief'.

FREQUENCIES RQ_C_Ant_B_N.

NUMERIC RQ_C_Adv_B_N (F2.0).

COMPUTE RQ_C_Adv_B_N = 1.

IF (RQ_C_Adv_B_M=1 OR (RQ_C_Adv_B_M = 2 AND OV_MeE_C_A=1)) RQ_C_Adv_B_N =2.

VARIABLE LABELS RQ_C_Adv_B_N 'Negatief krijgen zelfde adviezen, 2e helft zws'.

VALUE LABELS RQ_C_Adv_B_N

2 'negatief'

1 'positief'.

FREQUENCIES RQ_C_Adv_B_N.

NUMERIC RQ_C_Uit_B_N (F2.0).

COMPUTE RQ_C_Uit_B_N = 1.

IF (RQ_C_Uit_B_M=1 OR (RQ_C_Uit_B_M = 2 AND OV_MeE_C_A=1)) RQ_C_Uit_B_N =2.

VARIABLE LABELS RQ_C_Uit_B_N 'Negatief Begrijpen uitleg, 2e helft zws'.

VALUE LABELS RQ_C_Uit_B_N

2 'negatief'

1 'positief'.

FREQUENCIES RQ_C_Uit_B_N.

NUMERIC RQ_C_Inf_B_N (F2.0).

COMPUTE RQ_C_Inf_B_N = 1.

IF (RQ_C_Inf_B_M=1 OR (RQ_C_Inf_B_M = 2 AND OV_MeE_C_A=1)) RQ_C_Inf_B_N =2.

VARIABLE LABELS RQ_C_Inf_B_N 'Negatief Informatie tijdens behandeling, 2e helft zws'.

VALUE LABELS RQ_C_Inf_B_N

2 'negatief'

1 'positief'.

FREQUENCIES RQ_C_Inf_B_N.

*tijd tot hulp.

NUMERIC RQ_T_Ghd_B_N (F2.0).

COMPUTE RQ_T_Ghd_B_N = 1.

IF (RQ_T_Ghd_B_M_Cat5=1 OR (RQ_T_Ghd_B_M_Cat5 = 2 AND OV_MeE_T_A=1)) RQ_T_Ghd_B_N =2.

VARIABLE LABELS RQ_T_Ghd_B_N 'Negatief Hulp als dringend, 2e helft zws'.

VALUE LABELS RQ_T_Ghd_B_N

2 'negatief'

1 'positief'.

FREQUENCIES RQ_T_Ghd_B_N.

NUMERIC RQ_T_Gnd_B_N (F2.0).

COMPUTE RQ_T_Gnd_B_N = 1.

IF (RQ_T_Gnd_B_M=1 OR (RQ_T_Gnd_B_M = 2 AND OV_MeE_T_A=1)) RQ_T_Gnd_B_N =2.

VARIABLE LABELS RQ_T_Gnd_B_N 'Negatief Hulp als niet dringend, 2e helft zws'.

VALUE LABELS RQ_T_Gnd_B_N

2 'negatief'

1 'positief'.

FREQUENCIES RQ_T_Gnd_B_N.

NUMERIC RQ_T_TYD_B_N (F2.0).

COMPUTE RQ_T_TYD_B_N = 1.

IF (RQ_T_Tyd_B_M=1 OR (RQ_T_Tyd_B_M = 2 AND OV_MeE_T_A=1)) RQ_T_TYD_B_N =2.

VARIABLE LABELS RQ_T_TYD_B_N 'Negatief Tijd als nodig, 2e helft zws'.

VALUE LABELS RQ_T_TYD_B_N

2 'negatief'

1 'positief'.

FREQUENCIES RQ_T_TYD_B_N.

NUMERIC RQ_T_ANW_B_N (F2.0).

COMPUTE RQ_T_ANW_B_N = 1.

IF (RQ_T_Anw_B_M=1 OR (RQ_T_Anw_B_M = 2 AND OV_MeE_T_A=1)) RQ_T_ANW_B_N =2.

VARIABLE LABELS RQ_T_ANW_B_N 'Negatief Bij afspraak snel aan de beurt, 2e helft zws'.

VALUE LABELS RQ_T_ANW_B_N

2 'negatief'

1 'positief'.

FREQUENCIES RQ_T_ANW_B_N.

NUMERIC RQ_T_BER_B_N (F2.0).

COMPUTE RQ_T_BER_B_N = 1.

IF (RQ_T_Ber_B_M=1 OR (RQ_T_Ber_B_M = 2 AND OV_MeE_T_A=1)) RQ_T_BER_B_N =2.

VARIABLE LABELS RQ_T_BER_B_N 'Negatief bereikbaarheid locatie, 2e helft zws'.

VALUE LABELS RQ_T_BER_B_N

2 'negatief'

1 'positief'.

FREQUENCIES RQ_T_BER_B_N.

NUMERIC RQ_T_TEL_B_N (F2.0).

COMPUTE RQ_T_TEL_B_N = 1.

IF (RQ_T_Tel_B_M=1 OR (RQ_T_Tel_B_M = 2 AND OV_MeE_T_A=1)) RQ_T_TEL_B_N =2.

VARIABLE LABELS RQ_T_TEL_B_N 'Negatief telefonische bereikbaarheid, 2e helft zws'.

VALUE LABELS RQ_T_TEL_B_N

2 'negatief'

1 'positief'.

FREQUENCIES RQ_T_TEL_B_N.

*Sociale ondersteuning.

NUMERIC RQ_S_Fam_B_N (F2.0).

COMPUTE RQ_S_Fam_B_N = 1.

IF (RQ_S_Fam_B_M_Cat5=1 OR (RQ_S_Fam_B_M_Cat5 = 2 AND OV_MeE_S_A=1)) RQ_S_Fam_B_N =2.

VARIABLE LABELS RQ_S_Fam_B_N 'Negatief Betrekken familie, 2e helft zws'.

VALUE LABELS RQ_S_Fam_B_N

2 'negatief'

1 'positief'.

FREQUENCIES RQ_S_Fam_B_N.

NUMERIC RQ_S_Rhm_B_N (F2.0).

COMPUTE RQ_S_Rhm_B_N = 1.

IF (RQ_S_Rhm_B_M=1 OR (RQ_S_Rhm_B_M = 2 AND OV_MeE_S_A=1)) RQ_S_Rhm_B_N =2.

VARIABLE LABELS RQ_S_Rhm_B_N 'Negatief Rekening houden met gezin, 2e helft zws'.

VALUE LABELS RQ_S_Rhm_B_N

2 'negatief'

1 'positief'.

FREQUENCIES RQ_S_Rhm_B_N.

NUMERIC RQ_S_Ste_B_N (F2.0).

COMPUTE RQ_S_Ste_B_N = 1.

IF (RQ_S_Ste_B_M=1 OR (RQ_S_Ste_B_M = 2 AND OV_MeE_S_A=1)) RQ_S_Ste_B_N =2.

VARIABLE LABELS RQ_S_Ste_B_N 'Negatief Gesteund voelen, 2e helft zws'.

VALUE LABELS RQ_S_Ste_B_N

2 'negatief'

1 'positief'.

FREQUENCIES RQ_S_Ste_B_N.

*Faciliteiten.

NUMERIC RQ_F_Cmf_B_N (F2.0).

COMPUTE RQ_F_Cmf_B_N = 1.

IF (RQ_F_Cmf_B_M=1 OR (RQ_F_Cmf_B_M = 2 AND OV_MeE_F_A=1)) RQ_F_Cmf_B_N =2.

VARIABLE LABELS RQ_F_Cmf_B_N 'Negatief Comfort, 2e helft zws'.

VALUE LABELS RQ_F_Cmf_B_N

2 'negatief'

1 'positief'.

FREQUENCIES RQ_F_Cmf_B_N.

NUMERIC RQ_F_Hyg_B_N (F2.0).

COMPUTE RQ_F_Hyg_B_N = 1.

IF (RQ_F_Hyg_B_M=1 OR (RQ_F_Hyg_B_M = 2 AND OV_MeE_F_A=1)) RQ_F_Hyg_B_N =2.

VARIABLE LABELS RQ_F_Hyg_B_N 'Negatief Hygiene, 2e helft zws'.

VALUE LABELS RQ_F_Hyg_B_N

2 'negatief'

1 'positief'.

FREQUENCIES RQ_F_Hyg_B_N.

NUMERIC RQ_F_Toe_B_N (F2.0).

COMPUTE RQ_F_Toe_B_N = 1.

IF (RQ_F_Toe_B_M=1 OR (RQ_F_Toe_B_M = 2 AND OV_MeE_F_A=1)) RQ_F_Toe_B_N =2.

VARIABLE LABELS RQ_F_Toe_B_N 'Negatief Toegankelijkheid ruimtes, 2e helft zws'.

VALUE LABELS RQ_F_Toe_B_N

2 'negatief'

1 'positief'.

FREQUENCIES RQ_F_Toe_B_N.

*Keuze en continuiteit.

NUMERIC RQ_K_Wis_B_N (F2.0).

COMPUTE RQ_K_Wis_B_N = 1.

IF (RQ_K_Wis_B_M_Cat4=1 OR (RQ_K_Wis_B_M_Cat4 = 2 AND OV_MeE_K_A=1)) RQ_K_Wis_B_N =2.

VARIABLE LABELS RQ_K_Wis_B_N 'Negatief Wisselen zorgverlener, 2e helft zws'.

VALUE LABELS RQ_K_Wis_B_N

2 'negatief'

1 'positief'.

FREQUENCIES RQ_K_Wis_B_N.

NUMERIC RQ_K_Vwz_B_N (F2.0).

COMPUTE RQ_K_Vwz_B_N = 1.

IF (RQ_K_Vwz_B_M_Cat5=1 OR (RQ_K_Vwz_B_M_Cat5 = 2 AND OV_MeE_K_A=1)) RQ_K_Vwz_B_N =2.

VARIABLE LABELS RQ_K_Vwz_B_N 'Negatief Verwijzing naar het ziekenhuis, 2e helft zws'.

VALUE LABELS RQ_K_Vwz_B_N

2 'negatief'

1 'positief'.

FREQUENCIES RQ_K_Vwz_B_N.

NUMERIC RQ_K_Soo_B_N (F2.0).

COMPUTE RQ_K_Soo_B_N = 1.

IF (RQ_K_Soo_B_M=1 OR (RQ_K_Soo_B_M = 2 AND OV_MeE_K_A=1)) RQ_K_Soo_B_N =2.

VARIABLE LABELS RQ_K_Soo_B_N 'Negatief Keuze soort zorgverlener, 2e helft zws'.

VALUE LABELS RQ_K_Soo_B_N

2 'negatief'

1 'positief'.

FREQUENCIES RQ_K_Soo_B_N.

NUMERIC RQ_K_Lei_B_N (F2.0).

COMPUTE RQ_K_Lei_B_N = 1.

IF (RQ_K_Lei_B_M=1 OR (RQ_K_Lei_B_M = 2 AND OV_MeE_K_A=1)) RQ_K_Lei_B_N =2.

VARIABLE LABELS RQ_K_Lei_B_N 'Negatief Leiding zorg, 2e helft zws'.

VALUE LABELS RQ_K_Lei_B_N

2 'negatief'

1 'positief'.

FREQUENCIES RQ_K_Lei_B_N.

* STAP 2: berekenen percentage negatief op item niveau HERTEST.

*respect.

NUMERIC RQ_R_Pri_B_N_RE (F2.0).

COMPUTE RQ_R_Pri_B_N_RE = 1.

IF (RQ_R_Pri_B_RE_M=1 OR (RQ_R_Pri_B_RE_M = 2 AND OV_MeE_R_A=1)) RQ_R_Pri_B_N_RE =2.

VARIABLE LABELS RQ_R_Pri_B_N_RE 'Negatief Rekening houden met privacy, 2e helft zws'.

VALUE LABELS RQ_R_Pri_B_N_RE

2 'negatief'

1 'positief'.

FREQUENCIES RQ_R_Pri_B_N_RE.

NUMERIC RQ_R_RES_B_N_RE (F2.0).

COMPUTE RQ_R_RES_B_N_RE = 1.

IF (RQ_R_Res_B_RE_M=1 OR (RQ_R_Res_B_RE_M = 2 AND OV_MeE_R_A=1)) RQ_R_Res_B_N_RE =2.

VARIABLE LABELS RQ_R_RES_B_N_RE 'Negatief Behandeld met respect, 2e helft zws'.

VALUE LABELS RQ_R_RES_B_N_RE

2 'negatief'

1 'positief'.

FREQUENCIES RQ_R_RES_B_N_RE.

NUMERIC RQ_R_Per_B_N_RE (F2.0).

COMPUTE RQ_R_Per_B_N_RE = 1.

IF (RQ_R_Per_B_RE_M=1 OR (RQ_R_Per_B_RE_M = 2 AND OV_MeE_R_A=1)) RQ_R_Per_B_N_RE =2.

VARIABLE LABELS RQ_R_Per_B_N_RE 'Negatief Persoonlijke aandacht, 2e helft zws'.

VALUE LABELS RQ_R_Per_B_N_RE

2 'negatief'

1 'positief'.

FREQUENCIES RQ_R_Per_B_N_RE.

NUMERIC RQ_R_Vri_B_N_RE (F2.0).

COMPUTE RQ_R_Vri_B_N_RE = 1.

IF (RQ_R_Vri_B_RE_M=1 OR (RQ_R_Vri_B_RE_M = 2 AND OV_MeE_R_A=1)) RQ_R_Vri_B_N_RE =2.

VARIABLE LABELS RQ_R_Vri_B_N_RE 'Negatief Vriendelijk behandeld, 2e helft zws'.

VALUE LABELS RQ_R_Vri_B_N_RE

2 'negatief'

1 'positief'.

FREQUENCIES RQ_R_Vri_B_N_RE.

NUMERIC RQ_R_Wen_B_N_RE (F2.0).

COMPUTE RQ_R_Wen_B_N_RE = 1.

IF (RQ_R_Wen_B_RE_M=1 OR (RQ_R_Wen_B_RE_M = 2 AND OV_MeE_R_A=1)) RQ_R_Wen_B_N_RE =2.

VARIABLE LABELS RQ_R_Wen_B_N_RE 'Negatief Rekening houden met wensen en behoeften, 2e helft zws'.

VALUE LABELS RQ_R_Wen_B_N_RE

2 'negatief'

1 'positief'.

FREQUENCIES RQ_R_Wen_B_N_RE.

NUMERIC RQ_R_Ver_B_N_RE (F2.0).

COMPUTE RQ_R_Ver_B_N_RE = 1.

IF (RQ_R_Ver_B_RE_M=1 OR (RQ_R_Ver_B_RE_M = 2 AND OV_MeE_R_A=1)) RQ_R_Ver_B_N_RE =2.

VARIABLE LABELS RQ_R_Ver_B_N_RE 'Negatief Alles vertellen aan zorgverlener, 2e helft zws'.

VALUE LABELS RQ_R_Ver_B_N_RE

2 'negatief'

1 'positief'.

FREQUENCIES RQ_R_Ver_B_N_RE.

*autonomie.

NUMERIC RQ_A_Wei_B_N_Re (F2.0).

COMPUTE RQ_A_Wei_B_N_Re = 1.

IF (RQ_A_Wei_B_Re_M=1 OR (RQ_A_Wei_B_Re_M = 1 AND OV_MeE_A_A=1)) RQ_A_Wei_B_N_Re =2.

VARIABLE LABELS RQ_A_Wei_B_N_Re 'Negatief Weigeren behandeling, 2e helft zws, hertest'.

VALUE LABELS RQ_A_Wei_B_N_Re

2 'negatief'

1 'positief'.

FREQUENCIES RQ_A_Wei_B_N_Re.

NUMERIC RQ_A_Mee_B_N_Re (F2.0).

COMPUTE RQ_A_Mee_B_N_Re = 1.

IF (RQ_A_Mee_B_Re_M=1 OR (RQ_A_Mee_B_Re_M = 2 AND OV_MeE_A_A=1)) RQ_A_Mee_B_N_Re =2.

VARIABLE LABELS RQ_A_Mee_B_N_Re 'Negatief Meebeslissen, 2e helft zws, hertest'.

VALUE LABELS RQ_A_Mee_B_N_Re

2 'negatief'

1 'positief'.

FREQUENCIES RQ_A_Mee_B_N_Re.

NUMERIC RQ_A_SvD_B_N_Re (F2.0).

COMPUTE RQ_A_SvD_B_N_Re = 1.

IF (RQ_A_SvD_Re_M_Cat3=1 OR (RQ_A_SvD_Re_M_Cat3 = 2 AND OV_MeE_A_A=1)) RQ_A_SvD_B_N_Re =2.

VARIABLE LABELS RQ_A_SvD_B_N_Re 'Negatief Syndroom van Down, 2e helft zws, hertest'.

VALUE LABELS RQ_A_SvD_B_N_Re

2 'negatief'

1 'positief'.

FREQUENCIES RQ_A_SvD_B_N_Re.

NUMERIC RQ_A_Gbp_B_N_Re (F2.0).

COMPUTE RQ_A_Gbp_B_N_Re = 1.

IF (RQ_A_Gbp_Re_M_Cat5=1 OR (RQ_A_Gbp_Re_M_Cat5 = 2 AND OV_MeE_A_A=1)) RQ_A_Gbp_B_N_Re =2.

VARIABLE LABELS RQ_A_Gbp_B_N_Re 'Negatief Geboorteplan, 2e helft zws, hertest'.

VALUE LABELS RQ_A_Gbp_B_N_Re

2 'negatief'

1 'positief'.

FREQUENCIES RQ_A_Gbp_B_N_Re.

*privacy.

NUMERIC RQ_P_Med_B_N_RE (F2.0).

COMPUTE RQ_P_Med_B_N_RE = 1.

IF (RQ_P_Med_B_RE_M=1 OR (RQ_P_Med_B_RE_M = 1 AND OV_MeE_P_A=1)) RQ_P_Med_B_N_RE =2.

VARIABLE LABELS RQ_P_Med_B_N_RE 'Negatief Medische dossier, 2e helft zws, hertest'.

VALUE LABELS RQ_P_Med_B_N_RE

2 'negatief'

1 'positief'.

FREQUENCIES RQ_P_Med_B_N_RE.

NUMERIC RQ_P_Mln_B_N_RE (F2.0).

COMPUTE RQ_P_Mln_B_N_RE = 1.

IF (RQ_P_Mln_B_RE_M=1 OR (RQ_P_Mln_B_RE_M = 2 AND OV_MeE_P_A=1)) RQ_P_Mln_B_N_RE =2.

VARIABLE LABELS RQ_P_Mln_B_N_RE 'Negatief Meeluisteren, 2e helft zws, hertest'.

VALUE LABELS RQ_P_Mln_B_N_RE

2 'negatief'

1 'positief'.

FREQUENCIES RQ_P_Mln_B_N_RE.

*communicatie.

NUMERIC RQ_C_Ant_B_N_RE (F2.0).

COMPUTE RQ_C_Ant_B_N_RE = 1.

IF (RQ_C_Ant_B_RE_M=1 OR (RQ_C_Ant_B_RE_M = 2 AND OV_MeE_C_A=1)) RQ_C_Ant_B_N_RE =2.

VARIABLE LABELS RQ_C_Ant_B_N_RE 'Negatief Antwoord op vragen, 2e helft zws'.

VALUE LABELS RQ_C_Ant_B_N_RE

2 'negatief'

1 'positief'.

FREQUENCIES RQ_C_Ant_B_N_RE.

NUMERIC RQ_C_Adv_B_N_RE (F2.0).

COMPUTE RQ_C_Adv_B_N_RE = 1.

IF (RQ_C_Adv_B_RE_M=1 OR (RQ_C_Adv_B_RE_M = 2 AND OV_MeE_C_A=1)) RQ_C_Adv_B_N_RE =2.

VARIABLE LABELS RQ_C_Adv_B_N_RE 'Negatief krijgen zelfde adviezen, 2e helft zws'.

VALUE LABELS RQ_C_Adv_B_N_RE

2 'negatief'

1 'positief'.

FREQUENCIES RQ_C_Adv_B_N_RE.

NUMERIC RQ_C_Uit_B_N_RE (F2.0).

COMPUTE RQ_C_Uit_B_N_RE = 1.

IF (RQ_C_Uit_B_RE_M=1 OR (RQ_C_Uit_B_RE_M = 2 AND OV_MeE_C_A=1)) RQ_C_Uit_B_N_RE =2.

VARIABLE LABELS RQ_C_Uit_B_N_RE 'Negatief Begrijpen uitleg, 2e helft zws'.

VALUE LABELS RQ_C_Uit_B_N_RE

2 'negatief'

1 'positief'.

FREQUENCIES RQ_C_Uit_B_N_RE.

NUMERIC RQ_C_Inf_B_N_RE (F2.0).

COMPUTE RQ_C_Inf_B_N_RE = 1.

IF (RQ_C_Inf_B_RE_M=1 OR (RQ_C_Inf_B_RE_M = 2 AND OV_MeE_C_A=1)) RQ_C_Inf_B_N_RE =2.

VARIABLE LABELS RQ_C_Inf_B_N_RE 'Negatief Informatie tijdens behandeling, 2e helft zws'.

VALUE LABELS RQ_C_Inf_B_N_RE

2 'negatief'

1 'positief'.

FREQUENCIES RQ_C_Inf_B_N_RE.

*tijd tot hulp.

NUMERIC RQ_T_Ghd_B_N_RE (F2.0).

COMPUTE RQ_T_Ghd_B_N_RE = 1.

IF (RQ_T_Ghd_B_RE_M_Cat5=1 OR (RQ_T_Ghd_B_RE_M_Cat5 = 2 AND OV_MeE_T_A=1)) RQ_T_Ghd_B_N_RE =2.

VARIABLE LABELS RQ_T_Ghd_B_N_RE 'Negatief Hulp als dringend, 2e helft zws'.

VALUE LABELS RQ_T_Ghd_B_N_RE

2 'negatief'

1 'positief'.

FREQUENCIES RQ_T_Ghd_B_N_RE.

NUMERIC RQ_T_Gnd_B_N_RE (F2.0).

COMPUTE RQ_T_Gnd_B_N_RE = 1.

IF (RQ_T_Gnd_B_RE_M=1 OR (RQ_T_Gnd_B_RE_M = 2 AND OV_MeE_T_A=1)) RQ_T_Gnd_B_N_RE =2.

VARIABLE LABELS RQ_T_Gnd_B_N_RE 'Negatief Hulp als niet dringend, 2e helft zws'.

VALUE LABELS RQ_T_Gnd_B_N_RE

2 'negatief'

1 'positief'.

FREQUENCIES RQ_T_Gnd_B_N_RE.

NUMERIC RQ_T_TYD_B_N_RE (F2.0).

COMPUTE RQ_T_TYD_B_N_RE = 1.

IF (RQ_T_Tyd_B_RE_M=1 OR (RQ_T_Tyd_B_RE_M = 2 AND OV_MeE_T_A=1)) RQ_T_TYD_B_N_RE =2.

VARIABLE LABELS RQ_T_TYD_B_N_RE 'Negatief Tijd als nodig, 2e helft zws'.

VALUE LABELS RQ_T_TYD_B_N_RE

2 'negatief'

1 'positief'.

FREQUENCIES RQ_T_TYD_B_N_RE.

NUMERIC RQ_T_ANW_B_N_RE (F2.0).

COMPUTE RQ_T_ANW_B_N_RE = 1.

IF (RQ_T_Anw_B_RE_M=1 OR (RQ_T_Anw_B_RE_M = 2 AND OV_MeE_T_A=1)) RQ_T_ANW_B_N_RE =2.

VARIABLE LABELS RQ_T_ANW_B_N_RE 'Negatief Bij afspraak snel aan de beurt, 2e helft zws'.

VALUE LABELS RQ_T_ANW_B_N_RE

2 'negatief'

1 'positief'.

FREQUENCIES RQ_T_ANW_B_N_RE.

NUMERIC RQ_T_BER_B_N_RE (F2.0).

COMPUTE RQ_T_BER_B_N_RE = 1.

IF (RQ_T_Ber_B_RE_M=1 OR (RQ_T_Ber_B_RE_M = 2 AND OV_MeE_T_A=1)) RQ_T_BER_B_N_RE =2.

VARIABLE LABELS RQ_T_BER_B_N_RE 'Negatief bereikbaarheid locatie, 2e helft zws'.

VALUE LABELS RQ_T_BER_B_N_RE

2 'negatief'

1 'positief'.

FREQUENCIES RQ_T_BER_B_N_RE.

NUMERIC RQ_T_TEL_B_N_RE (F2.0).

COMPUTE RQ_T_TEL_B_N_RE = 1.

IF (RQ_T_Tel_B_RE_M=1 OR (RQ_T_Tel_B_RE_M = 2 AND OV_MeE_T_A=1)) RQ_T_TEL_B_N_RE =2.

VARIABLE LABELS RQ_T_TEL_B_N_RE 'Negatief telefonische bereikbaarheid, 2e helft zws'.

VALUE LABELS RQ_T_TEL_B_N_RE

2 'negatief'

1 'positief'.

FREQUENCIES RQ_T_TEL_B_N_RE.

*Sociale ondersteuning.

NUMERIC RQ_S_Fam_B_N_RE (F2.0).

COMPUTE RQ_S_Fam_B_N_RE = 1.

IF (RQ_S_Fam_B_RE_M_Cat5=1 OR (RQ_S_Fam_B_RE_M_Cat5 = 2 AND OV_MeE_S_A=1)) RQ_S_Fam_B_N_RE =2.

VARIABLE LABELS RQ_S_Fam_B_N_RE 'Negatief Betrekken familie, 2e helft zws'.

VALUE LABELS RQ_S_Fam_B_N_RE

2 'negatief'

1 'positief'.

FREQUENCIES RQ_S_Fam_B_N_RE.

NUMERIC RQ_S_Rhm_B_N_RE (F2.0).

COMPUTE RQ_S_Rhm_B_N_RE = 1.

IF (RQ_S_Rhm_B_RE_M=1 OR (RQ_S_Rhm_B_RE_M = 2 AND OV_MeE_S_A=1)) RQ_S_Rhm_B_N_RE =2.

VARIABLE LABELS RQ_S_Rhm_B_N_RE 'Negatief Rekening houden met gezin, 2e helft zws'.

VALUE LABELS RQ_S_Rhm_B_N_RE

2 'negatief'

1 'positief'.

FREQUENCIES RQ_S_Rhm_B_N_RE.

NUMERIC RQ_S_Ste_B_N_RE (F2.0).

COMPUTE RQ_S_Ste_B_N_RE = 1.

IF (RQ_S_Ste_B_RE_M=1 OR (RQ_S_Ste_B_RE_M = 2 AND OV_MeE_S_A=1)) RQ_S_Ste_B_N_RE =2.

VARIABLE LABELS RQ_S_Ste_B_N_RE 'Negatief Gesteund voelen, 2e helft zws'.

VALUE LABELS RQ_S_Ste_B_N_RE

2 'negatief'

1 'positief'.

FREQUENCIES RQ_S_Ste_B_N_RE.

*Faciliteiten.

NUMERIC RQ_F_Cmf_B_N_RE (F2.0).

COMPUTE RQ_F_Cmf_B_N_RE = 1.

IF (RQ_F_Cmf_B_RE_M=1 OR (RQ_F_Cmf_B_RE_M = 2 AND OV_MeE_F_A=1)) RQ_F_Cmf_B_N_RE =2.

VARIABLE LABELS RQ_F_Cmf_B_N_RE 'Negatief Comfort, 2e helft zws'.

VALUE LABELS RQ_F_Cmf_B_N_RE

2 'negatief'

1 'positief'.

FREQUENCIES RQ_F_Cmf_B_N_RE.

NUMERIC RQ_F_Hyg_B_N_RE (F2.0).

COMPUTE RQ_F_Hyg_B_N_RE = 1.

IF (RQ_F_Hyg_B_RE_M=1 OR (RQ_F_Hyg_B_RE_M = 2 AND OV_MeE_F_A=1)) RQ_F_Hyg_B_N_RE =2.

VARIABLE LABELS RQ_F_Hyg_B_N_RE 'Negatief Hygiene, 2e helft zws'.

VALUE LABELS RQ_F_Hyg_B_N_RE

2 'negatief'

1 'positief'.

FREQUENCIES RQ_F_Hyg_B_N_RE.

NUMERIC RQ_F_Toe_B_N_RE (F2.0).

COMPUTE RQ_F_Toe_B_N_RE = 1.

IF (RQ_F_Toe_B_RE_M=1 OR (RQ_F_Toe_B_RE_M = 2 AND OV_MeE_F_A=1)) RQ_F_Toe_B_N_RE =2.

VARIABLE LABELS RQ_F_Toe_B_N_RE 'Negatief Toegankelijkheid ruimtes, 2e helft zws'.

VALUE LABELS RQ_F_Toe_B_N_RE

2 'negatief'

1 'positief'.

FREQUENCIES RQ_F_Toe_B_N_RE.

*Keuze en continuiteit.

NUMERIC RQ_K_Wis_B_N_RE (F2.0).

COMPUTE RQ_K_Wis_B_N_RE = 1.

IF (RQ_K_Wis_B_RE_M_Cat4=1 OR (RQ_K_Wis_B_RE_M_Cat4 = 2 AND OV_MeE_K_A=1)) RQ_K_Wis_B_N_RE =2.

VARIABLE LABELS RQ_K_Wis_B_N_RE 'Negatief Wisselen zorgverlener, 2e helft zws'.

VALUE LABELS RQ_K_Wis_B_N_RE

2 'negatief'

1 'positief'.

FREQUENCIES RQ_K_Wis_B_N_RE.

NUMERIC RQ_K_Vwz_B_N_RE (F2.0).

COMPUTE RQ_K_Vwz_B_N_RE = 1.

IF (RQ_K_Vwz_B_RE_M_Cat5=1 OR (RQ_K_Vwz_B_RE_M_Cat5 = 2 AND OV_MeE_K_A=1)) RQ_K_Vwz_B_N_RE =2.

VARIABLE LABELS RQ_K_Vwz_B_N_RE 'Negatief Verwijzing naar het ziekenhuis, 2e helft zws'.

VALUE LABELS RQ_K_Vwz_B_N_RE

2 'negatief'

1 'positief'.

FREQUENCIES RQ_K_Vwz_B_N_RE.

NUMERIC RQ_K_Soo_B_N_RE (F2.0).

COMPUTE RQ_K_Soo_B_N_RE = 1.

IF (RQ_K_Soo_B_RE_M=1 OR (RQ_K_Soo_B_RE_M = 2 AND OV_MeE_K_A=1)) RQ_K_Soo_B_N_RE =2.

VARIABLE LABELS RQ_K_Soo_B_N_RE 'Negatief Keuze soort zorgverlener, 2e helft zws'.

VALUE LABELS RQ_K_Soo_B_N_RE

2 'negatief'

1 'positief'.

FREQUENCIES RQ_K_Soo_B_N_RE.

NUMERIC RQ_K_Lei_B_N_RE (F2.0).

COMPUTE RQ_K_Lei_B_N_RE = 1.

IF (RQ_K_Lei_B_RE_M=1 OR (RQ_K_Lei_B_RE_M = 2 AND OV_MeE_K_A=1)) RQ_K_Lei_B_N_RE =2.

VARIABLE LABELS RQ_K_Lei_B_N_RE 'Negatief Leiding zorg, 2e helft zws'.

VALUE LABELS RQ_K_Lei_B_N_RE

2 'negatief'

1 'positief'.

FREQUENCIES RQ_K_Lei_B_N_RE.

*STAP 3:Berekenen absolute agreement % Negatief TEST-HERTEST.

*Respect.

NUMERIC RQ_R_Pri_B_N_AA_C3 (F2).

COMPUTE RQ_R_Pri_B_N_AA_C3 = $SYSMIS.

IF (RQ_R_Pri_B_N=RQ_R_Pri_B_N_RE) RQ_R_Pri_B_N_AA_C3 =1.

IF (RQ_R_Pri_B_N>RQ_R_Pri_B_N_RE) RQ_R_Pri_B_N_AA_C3 =3.

IF (RQ_R_Pri_B_N<RQ_R_Pri_B_N_RE) RQ_R_Pri_B_N_AA_C3 =2.

VARIABLE LABELS RQ_R_Pri_B_N_AA_C3 'Absoluut agreement voor "Rekening houden privacy" test-thentest - NEG, C3' .

VALUE LABELS RQ_R_Pri_B_N_AA_C3

1 'test-thentest gelijk'

2 'test positiever dan hertest'

3 'test negatiever dan hertest' .

FREQUENCIES RQ_R_Pri_B_N_AA_C3.

NUMERIC RQ_R_RES_B_N_AA_C3 (F2).

COMPUTE RQ_R_RES_B_N_AA_C3 = $SYSMIS.

IF (RQ_R_RES_B_N=RQ_R_RES_B_N_RE) RQ_R_RES_B_N_AA_C3 =1.

IF (RQ_R_RES_B_N>RQ_R_RES_B_N_RE) RQ_R_RES_B_N_AA_C3 =3.

IF (RQ_R_RES_B_N<RQ_R_RES_B_N_RE) RQ_R_RES_B_N_AA_C3 =2.

VARIABLE LABELS RQ_R_RES_B_N_AA_C3 'Absoluut agreement voor "Behandeld met respect" test-thentest - NEG, C3' .

VALUE LABELS RQ_R_RES_B_N_AA_C3

1 'test-thentest gelijk'

2 'test positiever dan hertest'

3 'test negatiever dan hertest' .

FREQUENCIES RQ_R_RES_B_N_AA_C3.

NUMERIC RQ_R_Per_B_N_AA_C3 (F2).

COMPUTE RQ_R_Per_B_N_AA_C3 = $SYSMIS.

IF (RQ_R_Per_B_N=RQ_R_Per_B_N_RE) RQ_R_Per_B_N_AA_C3 =1.

IF (RQ_R_Per_B_N>RQ_R_Per_B_N_RE) RQ_R_Per_B_N_AA_C3 =3.

IF (RQ_R_Per_B_N<RQ_R_Per_B_N_RE) RQ_R_Per_B_N_AA_C3 =2.

VARIABLE LABELS RQ_R_Per_B_N_AA_C3 'Absoluut agreement voor "Persoonlijke aandacht" test-thentest - NEG, C3' .

VALUE LABELS RQ_R_Per_B_N_AA_C3

1 'test-thentest gelijk'

2 'test positiever dan hertest'

3 'test negatiever dan hertest' .

FREQUENCIES RQ_R_Per_B_N_AA_C3.

NUMERIC RQ_R_Vri_B_N_AA_C3 (F2).

COMPUTE RQ_R_Vri_B_N_AA_C3 = $SYSMIS.

IF (RQ_R_Vri_B_N=RQ_R_Vri_B_N_RE) RQ_R_Vri_B_N_AA_C3 =1.

IF (RQ_R_Vri_B_N>RQ_R_Vri_B_N_RE) RQ_R_Vri_B_N_AA_C3 =3.

IF (RQ_R_Vri_B_N<RQ_R_Vri_B_N_RE) RQ_R_Vri_B_N_AA_C3 =2.

VARIABLE LABELS RQ_R_Vri_B_N_AA_C3 'Absoluut agreement voor "Vriendelijk behandeld" test-thentest - NEG, C3' .

VALUE LABELS RQ_R_Vri_B_N_AA_C3

1 'test-thentest gelijk'

2 'test positiever dan hertest'

3 'test negatiever dan hertest' .

FREQUENCIES RQ_R_Vri_B_N_AA_C3.

NUMERIC RQ_R_Wen_B_N_AA_C3 (F2).

COMPUTE RQ_R_Wen_B_N_AA_C3 = $SYSMIS.

IF (RQ_R_Wen_B_N=RQ_R_Wen_B_N_RE) RQ_R_Wen_B_N_AA_C3 =1.

IF (RQ_R_Wen_B_N>RQ_R_Wen_B_N_RE) RQ_R_Wen_B_N_AA_C3 =3.

IF (RQ_R_Wen_B_N<RQ_R_Wen_B_N_RE) RQ_R_Wen_B_N_AA_C3 =2.

VARIABLE LABELS RQ_R_Wen_B_N_AA_C3 'Absoluut agreement voor "Wensen en behoeften" test-thentest - NEG, C3' .

VALUE LABELS RQ_R_Wen_B_N_AA_C3

1 'test-thentest gelijk'

2 'test positiever dan hertest'

3 'test negatiever dan hertest' .

FREQUENCIES RQ_R_Wen_B_N_AA_C3.

NUMERIC RQ_R_Ver_B_N_AA_C3 (F2).

COMPUTE RQ_R_Ver_B_N_AA_C3 = $SYSMIS.

IF (RQ_R_Ver_B_N=RQ_R_Ver_B_N_RE) RQ_R_Ver_B_N_AA_C3 =1.

IF (RQ_R_Ver_B_N>RQ_R_Ver_B_N_RE) RQ_R_Ver_B_N_AA_C3 =3.

IF (RQ_R_Ver_B_N<RQ_R_Ver_B_N_RE) RQ_R_Ver_B_N_AA_C3 =2.

VARIABLE LABELS RQ_R_Ver_B_N_AA_C3 'Absoluut agreement voor "Vertrouwen" test-thentest - NEG, C3' .

VALUE LABELS RQ_R_Ver_B_N_AA_C3

1 'test-thentest gelijk'

2 'test positiever dan hertest'

3 'test negatiever dan hertest' .

FREQUENCIES RQ_R_Ver_B_N_AA_C3.

*autonomie.

NUMERIC RQ_A_Wei_B_N_AA_C3 (F2).

COMPUTE RQ_A_Wei_B_N_AA_C3 = $SYSMIS.

IF (RQ_A_Wei_B_N=RQ_A_Wei_B_N_Re) RQ_A_Wei_B_N_AA_C3 =1.

IF (RQ_A_Wei_B_N>RQ_A_Wei_B_N_Re) RQ_A_Wei_B_N_AA_C3 =3.

IF (RQ_A_Wei_B_N<RQ_A_Wei_B_N_Re) RQ_A_Wei_B_N_AA_C3 =2.

VARIABLE LABELS RQ_A_Wei_B_N_AA_C3 'Absoluut agreement voor "weigeren behandeling" test-thentest - NEG, C3' .

VALUE LABELS RQ_A_Wei_B_N_AA_C3

1 'test-thentest gelijk'

2 'test positiever dan hertest'

3 'test negatiever dan hertest' .

FREQUENCIES RQ_A_Wei_B_N_AA_C3.

NUMERIC RQ_A_Mee_B_N_AA_C3 (F2).

COMPUTE RQ_A_Mee_B_N_AA_C3 = $SYSMIS.

IF (RQ_A_Mee_B_N=RQ_A_Mee_B_N_Re) RQ_A_Mee_B_N_AA_C3 =1.

IF (RQ_A_Mee_B_N>RQ_A_Mee_B_N_Re) RQ_A_Mee_B_N_AA_C3 =3.

IF (RQ_A_Mee_B_N<RQ_A_Mee_B_N_Re) RQ_A_Mee_B_N_AA_C3 =2.

VARIABLE LABELS RQ_A_Mee_B_N_AA_C3 'Absoluut agreement voor "Meebeslissen behandeling" test-thentest - NEG, C3' .

VALUE LABELS RQ_A_Mee_B_N_AA_C3

1 'test-thentest gelijk'

2 'test positiever dan hertest'

3 'test negatiever dan hertest' .

FREQUENCIES RQ_A_Mee_B_N_AA_C3.

NUMERIC RQ_A_SvD_B_N_AA_C3 (F2).

COMPUTE RQ_A_SvD_B_N_AA_C3 = $SYSMIS.

IF (RQ_A_SvD_B_N=RQ_A_SvD_B_N_Re) RQ_A_SvD_B_N_AA_C3 =1.

IF (RQ_A_SvD_B_N>RQ_A_SvD_B_N_Re) RQ_A_SvD_B_N_AA_C3 =3.

IF (RQ_A_SvD_B_N<RQ_A_SvD_B_N_Re) RQ_A_SvD_B_N_AA_C3 =2.

VARIABLE LABELS RQ_A_SvD_B_N_AA_C3 'Absoluut agreement voor "Syndroom van down" test-thentest - NEG, C3' .

VALUE LABELS RQ_A_SvD_B_N_AA_C3

1 'test-thentest gelijk'

2 'test positiever dan hertest'

3 'test negatiever dan hertest' .

FREQUENCIES RQ_A_SvD_B_N_AA_C3.

NUMERIC RQ_A_Gbp_B_N_AA_C3 (F2).

COMPUTE RQ_A_Gbp_B_N_AA_C3 = $SYSMIS.

IF (RQ_A_GBP_B_N=RQ_A_Gbp_B_N_Re) RQ_A_Gbp_B_N_AA_C3 =1.

IF (RQ_A_GBP_B_N>RQ_A_Gbp_B_N_Re) RQ_A_Gbp_B_N_AA_C3 =3.

IF (RQ_A_GBP_B_N<RQ_A_Gbp_B_N_Re) RQ_A_Gbp_B_N_AA_C3 =2.

VARIABLE LABELS RQ_A_Gbp_B_N_AA_C3 'Absoluut agreement voor "Geboorteplan" test-thentest - NEG, C3' .

VALUE LABELS RQ_A_Gbp_B_N_AA_C3

1 'test-thentest gelijk'

2 'test positiever dan hertest'

3 'test negatiever dan hertest' .

FREQUENCIES RQ_A_Gbp_B_N_AA_C3.

*Privacy.

NUMERIC RQ_P_Med_B_N_AA_C3 (F2).

COMPUTE RQ_P_Med_B_N_AA_C3 = $SYSMIS.

IF (RQ_P_Med_B_N=RQ_P_Med_B_N_RE) RQ_P_Med_B_N_AA_C3 =1.

IF (RQ_P_Med_B_N>RQ_P_Med_B_N_RE) RQ_P_Med_B_N_AA_C3 =3.

IF (RQ_P_Med_B_N<RQ_P_Med_B_N_RE) RQ_P_Med_B_N_AA_C3 =2.

VARIABLE LABELS RQ_P_Med_B_N_AA_C3 'Absoluut agreement voor "Medisch dossier" test-thentest - NEG, C3' .

VALUE LABELS RQ_P_Med_B_N_AA_C3

1 'test-thentest gelijk'

2 'test positiever dan hertest'

3 'test negatiever dan hertest' .

FREQUENCIES RQ_P_Med_B_N_AA_C3 RQ_P_Med_B_N RQ_P_Med_B_N_RE.

NUMERIC RQ_P_MLN_B_N_AA_C3 (F2).

COMPUTE RQ_P_MLN_B_N_AA_C3 = $SYSMIS.

IF (RQ_P_Mln_B_N=RQ_P_Mln_B_N_RE) RQ_P_MLN_B_N_AA_C3 =1.

IF (RQ_P_Mln_B_N>RQ_P_Mln_B_N_RE) RQ_P_MLN_B_N_AA_C3 =3.

IF (RQ_P_Mln_B_N<RQ_P_Mln_B_N_RE) RQ_P_MLN_B_N_AA_C3 =2.

VARIABLE LABELS RQ_P_MLN_B_N_AA_C3 'Absoluut agreement voor "Meeluisteren" test-thentest - NEG, C3' .

VALUE LABELS RQ_P_MLN_B_N_AA_C3

1 'test-thentest gelijk'

2 'test positiever dan hertest'

3 'test negatiever dan hertest' .

FREQUENCIES RQ_P_MLN_B_N_AA_C3.

*Communicatie.

NUMERIC RQ_C_Ant_B_N_AA_C3 (F2).

COMPUTE RQ_C_Ant_B_N_AA_C3 = $SYSMIS.

IF (RQ_C_Ant_B_N=RQ_C_Ant_B_N_RE) RQ_C_Ant_B_N_AA_C3 =1.

IF (RQ_C_Ant_B_N>RQ_C_Ant_B_N_RE) RQ_C_Ant_B_N_AA_C3 =3.

IF (RQ_C_Ant_B_N<RQ_C_Ant_B_N_RE) RQ_C_Ant_B_N_AA_C3 =2.

VARIABLE LABELS RQ_C_Ant_B_N_AA_C3 'Absoluut agreement voor "Antwoord op vragen" test-thentest - NEG, C3' .

VALUE LABELS RQ_C_Ant_B_N_AA_C3

1 'test-thentest gelijk'

2 'test positiever dan hertest'

3 'test negatiever dan hertest' .

FREQUENCIES RQ_C_Ant_B_N_AA_C3.

NUMERIC RQ_C_Adv_B_N_AA_C3 (F2).

COMPUTE RQ_C_Adv_B_N_AA_C3 = $SYSMIS.

IF (RQ_C_Adv_B_N=RQ_C_Adv_B_N_RE) RQ_C_Adv_B_N_AA_C3 =1.

IF (RQ_C_Adv_B_N>RQ_C_Adv_B_N_RE) RQ_C_Adv_B_N_AA_C3 =3.

IF (RQ_C_Adv_B_N<RQ_C_Adv_B_N_RE) RQ_C_Adv_B_N_AA_C3 =2.

VARIABLE LABELS RQ_C_Adv_B_N_AA_C3 'Absoluut agreement voor "Krijgen dezelfde adviezen" test-thentest - NEG, C3' .

VALUE LABELS RQ_C_Adv_B_N_AA_C3

1 'test-thentest gelijk'

2 'test positiever dan hertest'

3 'test negatiever dan hertest' .

FREQUENCIES RQ_C_Adv_B_N_AA_C3.

NUMERIC RQ_C_Uit_B_N_AA_C3 (F2).

COMPUTE RQ_C_Uit_B_N_AA_C3 = $SYSMIS.

IF (RQ_C_Uit_B_N=RQ_C_Uit_B_N_RE) RQ_C_Uit_B_N_AA_C3 =1.

IF (RQ_C_Uit_B_N>RQ_C_Uit_B_N_RE) RQ_C_Uit_B_N_AA_C3 =3.

IF (RQ_C_Uit_B_N<RQ_C_Uit_B_N_RE) RQ_C_Uit_B_N_AA_C3 =2.

VARIABLE LABELS RQ_C_Uit_B_N_AA_C3 'Absoluut agreement voor "Begrijpen uitleg" test-thentest - NEG, C3' .

VALUE LABELS RQ_C_Uit_B_N_AA_C3

1 'test-thentest gelijk'

2 'test positiever dan hertest'

3 'test negatiever dan hertest' .

FREQUENCIES RQ_C_Uit_B_N_AA_C3.

NUMERIC RQ_C_Inf_B_N_AA_C3 (F2).

COMPUTE RQ_C_Inf_B_N_AA_C3 = $SYSMIS.

IF (RQ_C_Inf_B_N=RQ_C_Inf_B_N_RE) RQ_C_Inf_B_N_AA_C3 =1.

IF (RQ_C_Inf_B_N>RQ_C_Inf_B_N_RE) RQ_C_Inf_B_N_AA_C3 =3.

IF (RQ_C_Inf_B_N<RQ_C_Inf_B_N_RE) RQ_C_Inf_B_N_AA_C3 =2.

VARIABLE LABELS RQ_C_Inf_B_N_AA_C3 'Absoluut agreement voor "Informatie tijdens behandeling" test-thentest - NEG, C3' .

VALUE LABELS RQ_C_Inf_B_N_AA_C3

1 'test-thentest gelijk'

2 'test positiever dan hertest'

3 'test negatiever dan hertest' .

FREQUENCIES RQ_C_Inf_B_N_AA_C3.

*Tijd tot hulp.

NUMERIC RQ_T_Ghd_B_N_AA_C3 (F2).

COMPUTE RQ_T_Ghd_B_N_AA_C3 = $SYSMIS.

IF (RQ_T_Ghd_B_N=RQ_T_Ghd_B_N_RE) RQ_T_Ghd_B_N_AA_C3 =1.

IF (RQ_T_Ghd_B_N>RQ_T_Ghd_B_N_RE) RQ_T_Ghd_B_N_AA_C3 =3.

IF (RQ_T_Ghd_B_N<RQ_T_Ghd_B_N_RE) RQ_T_Ghd_B_N_AA_C3 =2.

VARIABLE LABELS RQ_T_Ghd_B_N_AA_C3 'Absoluut agreement voor "Hulp als dringend" test-thentest - NEG, C3' .

VALUE LABELS RQ_T_Ghd_B_N_AA_C3

1 'test-thentest gelijk'

2 'test positiever dan hertest'

3 'test negatiever dan hertest' .

FREQUENCIES RQ_T_Ghd_B_N_AA_C3.

NUMERIC RQ_T_Gnd_B_N_AA_C3 (F2).

COMPUTE RQ_T_Gnd_B_N_AA_C3 = $SYSMIS.

IF (RQ_T_Gnd_B_N=RQ_T_Gnd_B_N_RE) RQ_T_Gnd_B_N_AA_C3 =1.

IF (RQ_T_Gnd_B_N>RQ_T_Gnd_B_N_RE) RQ_T_Gnd_B_N_AA_C3 =3.

IF (RQ_T_Gnd_B_N<RQ_T_Gnd_B_N_RE) RQ_T_Gnd_B_N_AA_C3 =2.

VARIABLE LABELS RQ_T_Gnd_B_N_AA_C3 'Absoluut agreement voor "Hulp als niet dringend" test-thentest - NEG, C3' .

VALUE LABELS RQ_T_Gnd_B_N_AA_C3

1 'test-thentest gelijk'

2 'test positiever dan hertest'

3 'test negatiever dan hertest' .

FREQUENCIES RQ_T_Gnd_B_N_AA_C3.

NUMERIC RQ_T_Tyd_B_N_AA_C3 (F2).

COMPUTE RQ_T_Tyd_B_N_AA_C3 = $SYSMIS.

IF (RQ_T_TYD_B_N=RQ_T_TYD_B_N_RE) RQ_T_Tyd_B_N_AA_C3 =1.

IF (RQ_T_TYD_B_N>RQ_T_TYD_B_N_RE) RQ_T_Tyd_B_N_AA_C3 =3.

IF (RQ_T_TYD_B_N<RQ_T_TYD_B_N_RE) RQ_T_Tyd_B_N_AA_C3 =2.

VARIABLE LABELS RQ_T_Tyd_B_N_AA_C3 'Absoluut agreement voor "Tijd als nodig" test-thentest - NEG, C3' .

VALUE LABELS RQ_T_Tyd_B_N_AA_C3

1 'test-thentest gelijk'

2 'test positiever dan hertest'

3 'test negatiever dan hertest' .

FREQUENCIES RQ_T_Tyd_B_N_AA_C3.

NUMERIC RQ_T_Anw_B_N_AA_C3 (F2).

COMPUTE RQ_T_Anw_B_N_AA_C3 = $SYSMIS.

IF (RQ_T_ANW_B_N=RQ_T_ANW_B_N_RE) RQ_T_Anw_B_N_AA_C3 =1.

IF (RQ_T_ANW_B_N>RQ_T_ANW_B_N_RE) RQ_T_Anw_B_N_AA_C3 =3.

IF (RQ_T_ANW_B_N<RQ_T_ANW_B_N_RE) RQ_T_Anw_B_N_AA_C3 =2.

VARIABLE LABELS RQ_T_Anw_B_N_AA_C3 'Absoluut agreement voor "bij afspraak snel aan de beurt" test-thentest - NEG, C3' .

VALUE LABELS RQ_T_Anw_B_N_AA_C3

1 'test-thentest gelijk'

2 'test positiever dan hertest'

3 'test negatiever dan hertest' .

FREQUENCIES RQ_T_Anw_B_N_AA_C3.

NUMERIC RQ_T_BER_B_N_AA_C3 (F2).

COMPUTE RQ_T_BER_B_N_AA_C3 = $SYSMIS.

IF (RQ_T_BER_B_N=RQ_T_BER_B_N_RE) RQ_T_BER_B_N_AA_C3 =1.

IF (RQ_T_BER_B_N>RQ_T_BER_B_N_RE) RQ_T_BER_B_N_AA_C3 =3.

IF (RQ_T_BER_B_N<RQ_T_BER_B_N_RE) RQ_T_BER_B_N_AA_C3 =2.

VARIABLE LABELS RQ_T_BER_B_N_AA_C3 'Absoluut agreement voor "Bereikbaarheid locatie" test-thentest - NEG, C3' .

VALUE LABELS RQ_T_BER_B_N_AA_C3

1 'test-thentest gelijk'

2 'test positiever dan hertest'

3 'test negatiever dan hertest' .

FREQUENCIES RQ_T_BER_B_N_AA_C3.

NUMERIC RQ_T_TEL_B_N_AA_C3 (F2).

COMPUTE RQ_T_TEL_B_N_AA_C3 = $SYSMIS.

IF (RQ_T_TEL_B_N=RQ_T_TEL_B_N_RE) RQ_T_TEL_B_N_AA_C3 =1.

IF (RQ_T_TEL_B_N>RQ_T_TEL_B_N_RE) RQ_T_TEL_B_N_AA_C3 =3.

IF (RQ_T_TEL_B_N<RQ_T_TEL_B_N_RE) RQ_T_TEL_B_N_AA_C3 =2.

VARIABLE LABELS RQ_T_TEL_B_N_AA_C3 'Absoluut agreement voor "Telefonische bereikbaarheid" test-thentest - NEG, C3' .

VALUE LABELS RQ_T_TEL_B_N_AA_C3

1 'test-thentest gelijk'

2 'test positiever dan hertest'

3 'test negatiever dan hertest' .

FREQUENCIES RQ_T_TEL_B_N_AA_C3.

*Sociale ondersteuning.

NUMERIC RQ_S_Fam_B_N_AA_C3 (F2).

COMPUTE RQ_S_Fam_B_N_AA_C3 = $SYSMIS.

IF (RQ_S_Fam_B_N=RQ_S_Fam_B_N_RE) RQ_S_Fam_B_N_AA_C3 =1.

IF (RQ_S_Fam_B_N>RQ_S_Fam_B_N_RE) RQ_S_Fam_B_N_AA_C3 =3.

IF (RQ_S_Fam_B_N<RQ_S_Fam_B_N_RE) RQ_S_Fam_B_N_AA_C3 =2.

VARIABLE LABELS RQ_S_Fam_B_N_AA_C3 'Absoluut agreement voor "Betrekken familie" test-thentest - NEG, C3' .

VALUE LABELS RQ_S_Fam_B_N_AA_C3

1 'test-thentest gelijk'

2 'test positiever dan hertest'

3 'test negatiever dan hertest' .

FREQUENCIES RQ_S_Fam_B_N_AA_C3.

NUMERIC RQ_S_Rhm_B_N_AA_C3 (F2).

COMPUTE RQ_S_Rhm_B_N_AA_C3 = $SYSMIS.

IF (RQ_S_Rhm_B_N=RQ_S_Rhm_B_N_RE) RQ_S_Rhm_B_N_AA_C3 =1.

IF (RQ_S_Rhm_B_N>RQ_S_Rhm_B_N_RE) RQ_S_Rhm_B_N_AA_C3 =3.

IF (RQ_S_Rhm_B_N<RQ_S_Rhm_B_N_RE) RQ_S_Rhm_B_N_AA_C3 =2.

VARIABLE LABELS RQ_S_Rhm_B_N_AA_C3 'Absoluut agreement voor "Rekening houden met gezin" test-thentest - NEG, C3' .

VALUE LABELS RQ_S_Rhm_B_N_AA_C3

1 'test-thentest gelijk'

2 'test positiever dan hertest'

3 'test negatiever dan hertest' .

FREQUENCIES RQ_S_Rhm_B_N_AA_C3.

NUMERIC RQ_S_Ste_B_N_AA_C3 (F2).

COMPUTE RQ_S_Ste_B_N_AA_C3 = $SYSMIS.

IF (RQ_S_Ste_B_N=RQ_S_Ste_B_N_RE) RQ_S_Ste_B_N_AA_C3 =1.

IF (RQ_S_Ste_B_N>RQ_S_Ste_B_N_RE) RQ_S_Ste_B_N_AA_C3 =3.

IF (RQ_S_Ste_B_N<RQ_S_Ste_B_N_RE) RQ_S_Ste_B_N_AA_C3 =2.

VARIABLE LABELS RQ_S_Ste_B_N_AA_C3 'Absoluut agreement voor "Gesteund voelen" test-thentest - NEG, C3' .

VALUE LABELS RQ_S_Ste_B_N_AA_C3

1 'test-thentest gelijk'

2 'test positiever dan hertest'

3 'test negatiever dan hertest' .

FREQUENCIES RQ_S_Ste_B_N_AA_C3.

*Faciliteiten.

NUMERIC RQ_F_Cmf_B_N_AA_C3 (F2).

COMPUTE RQ_F_Cmf_B_N_AA_C3 = $SYSMIS.

IF (RQ_F_Cmf_B_N=RQ_F_Cmf_B_N_RE) RQ_F_Cmf_B_N_AA_C3 =1.

IF (RQ_F_Cmf_B_N>RQ_F_Cmf_B_N_RE) RQ_F_Cmf_B_N_AA_C3 =3.

IF (RQ_F_Cmf_B_N<RQ_F_Cmf_B_N_RE) RQ_F_Cmf_B_N_AA_C3 =2.

VARIABLE LABELS RQ_F_Cmf_B_N_AA_C3 'Absoluut agreement voor "Comfort" test-thentest - NEG, C3' .

VALUE LABELS RQ_F_Cmf_B_N_AA_C3

1 'test-thentest gelijk'

2 'test positiever dan hertest'

3 'test negatiever dan hertest' .

FREQUENCIES RQ_F_Cmf_B_N_AA_C3.

NUMERIC RQ_F_Hyg_B_N_AA_C3 (F2).

COMPUTE RQ_F_Hyg_B_N_AA_C3 = $SYSMIS.

IF (RQ_F_Hyg_B_N=RQ_F_Hyg_B_N_RE) RQ_F_Hyg_B_N_AA_C3 =1.

IF (RQ_F_Hyg_B_N>RQ_F_Hyg_B_N_RE) RQ_F_Hyg_B_N_AA_C3 =3.

IF (RQ_F_Hyg_B_N<RQ_F_Hyg_B_N_RE) RQ_F_Hyg_B_N_AA_C3 =2.

VARIABLE LABELS RQ_F_Hyg_B_N_AA_C3 'Absoluut agreement voor "Hygiene" test-thentest - NEG, C3' .

VALUE LABELS RQ_F_Hyg_B_N_AA_C3

1 'test-thentest gelijk'

2 'test positiever dan hertest'

3 'test negatiever dan hertest' .

FREQUENCIES RQ_F_Hyg_B_N_AA_C3.

NUMERIC RQ_F_Toe_B_N_AA_C3 (F2).

COMPUTE RQ_F_Toe_B_N_AA_C3 = $SYSMIS.

IF (RQ_F_Toe_B_N=RQ_F_Toe_B_N_RE) RQ_F_Toe_B_N_AA_C3 =1.

IF (RQ_F_Toe_B_N>RQ_F_Toe_B_N_RE) RQ_F_Toe_B_N_AA_C3 =3.

IF (RQ_F_Toe_B_N<RQ_F_Toe_B_N_RE) RQ_F_Toe_B_N_AA_C3 =2.

VARIABLE LABELS RQ_F_Toe_B_N_AA_C3 'Absoluut agreement voor "Toegankelijkheid ruimtes" test-thentest - NEG, C3' .

VALUE LABELS RQ_F_Toe_B_N_AA_C3

1 'test-thentest gelijk'

2 'test positiever dan hertest'

3 'test negatiever dan hertest' .

FREQUENCIES RQ_F_Toe_B_N_AA_C3.

*Keuze en continuiteit.

NUMERIC RQ_K_Wis_B_N_AA_C3 (F2).

COMPUTE RQ_K_Wis_B_N_AA_C3 = $SYSMIS.

IF (RQ_K_Wis_B_N=RQ_K_Wis_B_N_RE) RQ_K_Wis_B_N_AA_C3 =1.

IF (RQ_K_Wis_B_N>RQ_K_Wis_B_N_RE) RQ_K_Wis_B_N_AA_C3 =3.

IF (RQ_K_Wis_B_N<RQ_K_Wis_B_N_RE) RQ_K_Wis_B_N_AA_C3 =2.

VARIABLE LABELS RQ_K_Wis_B_N_AA_C3 'Absoluut agreement voor "Wisselen zorgverlener" test-thentest - NEG, C3' .

VALUE LABELS RQ_K_Wis_B_N_AA_C3

1 'test-thentest gelijk'

2 'test positiever dan hertest'

3 'test negatiever dan hertest' .

FREQUENCIES RQ_K_Wis_B_N_AA_C3.

NUMERIC RQ_K_Vwz_B_N_AA_C3 (F2).

COMPUTE RQ_K_Vwz_B_N_AA_C3 = $SYSMIS.

IF (RQ_K_Vwz_B_N=RQ_K_Vwz_B_N_RE) RQ_K_Vwz_B_N_AA_C3 =1.

IF (RQ_K_Vwz_B_N>RQ_K_Vwz_B_N_RE) RQ_K_Vwz_B_N_AA_C3 =3.

IF (RQ_K_Vwz_B_N<RQ_K_Vwz_B_N_RE) RQ_K_Vwz_B_N_AA_C3 =2.

VARIABLE LABELS RQ_K_Vwz_B_N_AA_C3 'Absoluut agreement voor "Verwijzen naar ziekenhuis" test-thentest - NEG, C3' .

VALUE LABELS RQ_K_Vwz_B_N_AA_C3

1 'test-thentest gelijk'

2 'test positiever dan hertest'

3 'test negatiever dan hertest' .

FREQUENCIES RQ_K_Vwz_B_N_AA_C3.

NUMERIC RQ_K_Soo_B_N_AA_C3 (F2).

COMPUTE RQ_K_Soo_B_N_AA_C3 = $SYSMIS.

IF (RQ_K_Soo_B_N=RQ_K_Soo_B_N_RE) RQ_K_Soo_B_N_AA_C3 =1.

IF (RQ_K_Soo_B_N>RQ_K_Soo_B_N_RE) RQ_K_Soo_B_N_AA_C3 =3.

IF (RQ_K_Soo_B_N<RQ_K_Soo_B_N_RE) RQ_K_Soo_B_N_AA_C3 =2.

VARIABLE LABELS RQ_K_Soo_B_N_AA_C3 'Absoluut agreement voor "Keuze soort zorgverlener" test-thentest - NEG, C3' .

VALUE LABELS RQ_K_Soo_B_N_AA_C3

1 'test-thentest gelijk'

2 'test positiever dan hertest'

3 'test negatiever dan hertest' .

FREQUENCIES RQ_K_Soo_B_N_AA_C3.

NUMERIC RQ_K_Lei_B_N_AA_C3 (F2).

COMPUTE RQ_K_Lei_B_N_AA_C3 = $SYSMIS.

IF (RQ_K_Lei_B_N=RQ_K_Lei_B_N_RE) RQ_K_Lei_B_N_AA_C3 =1.

IF (RQ_K_Lei_B_N>RQ_K_Lei_B_N_RE) RQ_K_Lei_B_N_AA_C3 =3.

IF (RQ_K_Lei_B_N<RQ_K_Lei_B_N_RE) RQ_K_Lei_B_N_AA_C3 =2.

VARIABLE LABELS RQ_K_Lei_B_N_AA_C3 'Absoluut agreement voor "Leiding zorg" test-thentest - NEG, C3' .

VALUE LABELS RQ_K_Lei_B_N_AA_C3

1 'test-thentest gelijk'

2 'test positiever dan hertest'

3 'test negatiever dan hertest' .

FREQUENCIES RQ_K_Lei_B_N_AA_C3.

### Median score

FREQUENCIES RQ_R_Pri_B_M RQ_R_Res_B_M RQ_R_Per_B_M RQ_R_Vri_B_M RQ_R_Wen_B_M RQ_R_Ver_B_M

RQ_A_Wei_B_M RQ_A_Mee_B_M RQ_A_SvD_M_Cat3 RQ_A_Gbp_B_M_Cat5

RQ_P_Bsp_B_M_Cat5 RQ_P_Med_B_M RQ_P_Mln_B_M

RQ_C_Ant_B_M RQ_C_Adv_B_M RQ_C_Uit_B_M RQ_C_Inf_B_M

RQ_T_Ghd_B_M_Cat5 RQ_T_Gnd_B_M RQ_T_Tyd_B_M RQ_T_Anw_B_M RQ_T_Ber_B_M RQ_T_Tel_B_M

RQ_S_Fam_B_M_Cat5 RQ_S_Rhm_B_M RQ_S_Ste_B_M

RQ_F_Cmf_B_M RQ_F_Hyg_B_M RQ_F_Toe_B_M

RQ_K_Wis_B_M_Cat4 RQ_K_Vwz_B_M_Cat5 RQ_K_Soo_B_M RQ_K_Lei_B_M

/STATISTICS median.

*MD TEST.

*Respect.

NUMERIC MD_R_Pri_B (F2).

COMPUTE MD_R_Pri_B =4.

VARIABLE LABELS MD_R_Pri_B 'Mediaan Rekening houden privacy, test'.

FREQUENCIES MD_R_Pri_B.

NUMERIC MD_R_Res_B (F2).

COMPUTE MD_R_Res_B =4.

VARIABLE LABELS MD_R_Res_B 'Mediaan Behandeld met respect, test'.

FREQUENCIES MD_R_Res_B.

NUMERIC MD_R_Per_B (F2).

COMPUTE MD_R_Per_B =4.

VARIABLE LABELS MD_R_Per_B 'Mediaan Persoonlijke aandacht, test'.

FREQUENCIES MD_R_Per_B.

NUMERIC MD_R_Vri_B (F2).

COMPUTE MD_R_Vri_B =4.

VARIABLE LABELS MD_R_Vri_B 'Mediaan Vriendelijk behandeld, test'.

FREQUENCIES MD_R_Vri_B.

NUMERIC MD_R_Wen_B (F2).

COMPUTE MD_R_Wen_B =4.

VARIABLE LABELS MD_R_Wen_B 'Mediaan Wensen en behoeften, test'.

FREQUENCIES MD_R_Wen_B.

NUMERIC MD_R_Ver_B (F2).

COMPUTE MD_R_Ver_B =4.

VARIABLE LABELS MD_R_Ver_B 'Mediaan Vertrouwen, test'.

FREQUENCIES MD_R_Ver_B.

*Autonomie.

NUMERIC MD_A_Wei_B (F2).

COMPUTE MD_A_Wei_B =4.

VARIABLE LABELS MD_A_Wei_B 'Mediaan weigeren behandeling, test'.

FREQUENCIES MD_A_Wei_B.

NUMERIC MD_A_Mee_B (F2).

COMPUTE MD_A_Mee_B =4.

VARIABLE LABELS MD_A_Mee_B 'Mediaan meebeslissen, test'.

FREQUENCIES MD_A_Mee_B.

NUMERIC MD_A_SvD_B (F2).

COMPUTE MD_A_SvD_B =4.

VARIABLE LABELS MD_A_SvD_B 'Mediaan syndroom van down, test'.

FREQUENCIES MD_A_SvD_B.

NUMERIC MD_A_GBP_B (F2).

COMPUTE MD_A_GBP_B =4.

VARIABLE LABELS MD_A_GBP_B 'Mediaan geboorteplan, test'.

FREQUENCIES MD_A_GBP_B.

*Privacy.

NUMERIC MD_P_Med_B (F2).

COMPUTE MD_P_Med_B =4.

VARIABLE LABELS MD_P_Med_B 'Mediaan Medisch dossier, test'.

FREQUENCIES MD_P_Med_B.

NUMERIC MD_P_Mln_B (F2).

COMPUTE MD_P_Mln_B =4.

VARIABLE LABELS MD_P_Mln_B 'Mediaan Meeluisteren, test'.

FREQUENCIES MD_P_Mln_B.

*Communicatie.

NUMERIC MD_C_Ant_B (F2).

COMPUTE MD_C_Ant_B =4.

VARIABLE LABELS MD_C_Ant_B 'Mediaan Antwoord op vragen, test'.

FREQUENCIES MD_C_Ant_B.

NUMERIC MD_C_Adv_B (F2).

COMPUTE MD_C_Adv_B =4.

VARIABLE LABELS MD_C_Adv_B 'Mediaan Krijgen zelfde adviezen, test'.

FREQUENCIES MD_C_Adv_B.

NUMERIC MD_C_Uit_B (F2).

COMPUTE MD_C_Uit_B =4.

VARIABLE LABELS MD_C_Uit_B 'Mediaan begerijpen uitleg, test'.

FREQUENCIES MD_C_Uit_B.

NUMERIC MD_C_Inf_B (F2).

COMPUTE MD_C_Inf_B =4.

VARIABLE LABELS MD_C_Inf_B 'Mediaan Informeren tijdens behandeling, test'.

FREQUENCIES MD_C_Inf_B.

*Tijd tot hulp.

NUMERIC MD_T_Ghd_B (F2).

COMPUTE MD_T_Ghd_B =4.

VARIABLE LABELS MD_T_Ghd_B 'Mediaan Hulp als dringend, test'.

FREQUENCIES MD_T_Ghd_B.

NUMERIC MD_T_Gnd_B (F2).

COMPUTE MD_T_Gnd_B =4.

VARIABLE LABELS MD_T_Gnd_B 'Mediaan Hulp als niet dringen, test'.

FREQUENCIES MD_T_Gnd_B.

NUMERIC MD_T_Tyd_B (F2).

COMPUTE MD_T_Tyd_B =4.

VARIABLE LABELS MD_T_Tyd_B 'Mediaan Tijd als behoeften, test'.

FREQUENCIES MD_T_Tyd_B.

NUMERIC MD_T_Anw_B (F2).

COMPUTE MD_T_Anw_B =3.

VARIABLE LABELS MD_T_Anw_B 'Mediaan Snel aan de beurt, test'.

FREQUENCIES MD_T_Anw_B.

NUMERIC MD_T_Ber_B (F2).

COMPUTE MD_T_Ber_B =4.

VARIABLE LABELS MD_T_Ber_B 'Mediaan Bereikbaarheid locatie, test'.

FREQUENCIES MD_T_Ber_B.

NUMERIC MD_T_Tel_B (F2).

COMPUTE MD_T_Tel_B =4.

VARIABLE LABELS MD_T_Tel_B 'Mediaan Telefonische bereikbaarheid, test'.

FREQUENCIES MD_T_Tel_B.

*Sociale ondersteuning.

NUMERIC MD_S_Fam_B (F2).

COMPUTE MD_S_Fam_B =4.

VARIABLE LABELS MD_S_Fam_B 'Mediaan Betrekken familie, test'.

FREQUENCIES MD_S_Fam_B.

NUMERIC MD_S_Rhm_B (F2).

COMPUTE MD_S_Rhm_B =4.

VARIABLE LABELS MD_S_Rhm_B 'Mediaan Rekening houden met gezin, test'.

FREQUENCIES MD_S_Rhm_B.

NUMERIC MD_S_Ste_B (F2).

COMPUTE MD_S_Ste_B =4.

VARIABLE LABELS MD_S_Ste_B 'Mediaan Gesteund voelen, test'.

FREQUENCIES MD_S_Ste_B.

*Faciliteiten.

NUMERIC MD_F_Cmf_B (F2).

COMPUTE MD_F_Cmf_B =4.

VARIABLE LABELS MD_F_Cmf_B 'Mediaan Comfort, test'.

FREQUENCIES MD_F_Cmf_B.

NUMERIC MD_F_Hyg_B (F2).

COMPUTE MD_F_Hyg_B =4.

VARIABLE LABELS MD_F_Hyg_B 'Mediaan Hygiene, test'.

FREQUENCIES MD_F_Hyg_B.

NUMERIC MD_F_Toe_B (F2).

COMPUTE MD_F_Toe_B =4.

VARIABLE LABELS MD_F_Toe_B 'Mediaan Toegankelijkheid ruimtes, test'.

FREQUENCIES MD_F_Toe_B.

*Keuze en continuiteit.

NUMERIC MD_K_Wis_B (F2).

COMPUTE MD_K_Wis_B =4.

VARIABLE LABELS MD_K_Wis_B 'Mediaan Wisselen zorgverlener, test'.

FREQUENCIES MD_K_Wis_B.

NUMERIC MD_K_Vwz_B (F2).

COMPUTE MD_K_Vwz_B =4.

VARIABLE LABELS MD_K_Vwz_B 'Mediaan Verwijzen ziekenhuis, test'.

FREQUENCIES MD_K_Vwz_B.

NUMERIC MD_K_Soo_B (F2).

COMPUTE MD_K_Soo_B =4.

VARIABLE LABELS MD_K_Soo_B 'Mediaan kiezen soort zorgverlener, test'.

FREQUENCIES MD_K_Soo_B.

NUMERIC MD_K_Lei_B (F2).

COMPUTE MD_K_Lei_B =4.

VARIABLE LABELS MD_K_Lei_B 'Mediaan leiding zorg, test'.

FREQUENCIES MD_K_Lei_B.

* STAP 1: berekenen percentage Onder mediaan op item niveau TEST.

*respect.

NUMERIC RQ_R_Pri_B_MD (F2.0).

COMPUTE RQ_R_Pri_B_MD = 1.

IF (RQ_R_Pri_B_M<MD_R_Pri_B) RQ_R_Pri_B_MD =2.

VARIABLE LABELS RQ_R_Pri_B_MD 'Onder mediaan Rekening houden met privacy, B - test'.

VALUE LABELS RQ_R_Pri_B_MD

2 'Onder mediaan'

1 'Gelijk of boven mediaan'.

FREQUENCIES RQ_R_Pri_B_MD.

NUMERIC RQ_R_RES_B_MD (F2.0).

COMPUTE RQ_R_RES_B_MD = 1.

IF (RQ_R_Res_B_M<MD_R_Res_B) RQ_R_Res_B_MD =2.

VARIABLE LABELS RQ_R_RES_B_MD 'Onder mediaan Behandeld met respect, B - test'.

VALUE LABELS RQ_R_RES_B_MD

2 'Onder mediaan'

1 'Gelijk of boven mediaan'.

FREQUENCIES RQ_R_RES_B_MD.

NUMERIC RQ_R_Per_B_MD (F2.0).

COMPUTE RQ_R_Per_B_MD = 1.

IF (RQ_R_Per_B_M<MD_R_Per_B) RQ_R_Per_B_MD =2.

VARIABLE LABELS RQ_R_Per_B_MD 'Onder mediaan Persoonlijke aandacht, B - test'.

VALUE LABELS RQ_R_Per_B_MD

2 'Onder mediaan'

1 'Gelijk of boven mediaan'.

FREQUENCIES RQ_R_Per_B_MD.

NUMERIC RQ_R_Vri_B_MD (F2.0).

COMPUTE RQ_R_Vri_B_MD = 1.

IF (RQ_R_Vri_B_M<MD_R_Vri_B) RQ_R_Vri_B_MD =2.

VARIABLE LABELS RQ_R_Vri_B_MD 'Onder mediaan Vriendelijk behandeld, B - test'.

VALUE LABELS RQ_R_Vri_B_MD

2 'Onder mediaan'

1 'Gelijk of boven mediaan'.

FREQUENCIES RQ_R_Vri_B_MD.

NUMERIC RQ_R_Wen_B_MD (F2.0).

COMPUTE RQ_R_Wen_B_MD = 1.

IF (RQ_R_Wen_B_M<MD_R_Wen_B) RQ_R_Wen_B_MD =2.

VARIABLE LABELS RQ_R_Wen_B_MD 'Onder mediaan Rekening houden met wensen en behoeften, B - test'.

VALUE LABELS RQ_R_Wen_B_MD

2 'Onder mediaan'

1 'Gelijk of boven mediaan'.

FREQUENCIES RQ_R_Wen_B_MD.

NUMERIC RQ_R_Ver_B_MD (F2.0).

COMPUTE RQ_R_Ver_B_MD = 1.

IF (RQ_R_Ver_B_M<MD_R_Ver_B) RQ_R_Ver_B_MD =2.

VARIABLE LABELS RQ_R_Ver_B_MD 'Onder mediaan Alles vertellen aan zorgverlener, B - test'.

VALUE LABELS RQ_R_Ver_B_MD

2 'Onder mediaan'

1 'Gelijk of boven mediaan'.

FREQUENCIES RQ_R_Ver_B_MD.

*autonomie.

NUMERIC RQ_A_Wei_B_MD (F2.0).

COMPUTE RQ_A_Wei_B_MD = 1.

IF (RQ_A_Wei_B_M<MD_A_Wei_B) RQ_A_Wei_B_MD =2.

VARIABLE LABELS RQ_A_Wei_B_MD 'Onder mediaan Weigeren behandeling, B - test'.

VALUE LABELS RQ_A_Wei_B_MD

2 'Onder mediaan'

1 'Gelijk of boven mediaan'.

FREQUENCIES RQ_A_Wei_B_MD.

NUMERIC RQ_A_Mee_B_MD (F2.0).

COMPUTE RQ_A_Mee_B_MD = 1.

IF (RQ_A_Mee_B_M<MD_A_Mee_B) RQ_A_Mee_B_MD =2.

VARIABLE LABELS RQ_A_Mee_B_MD 'Onder mediaan Meebeslissen, B - test'.

VALUE LABELS RQ_A_Mee_B_MD

2 'Onder mediaan'

1 'Gelijk of boven mediaan'.

FREQUENCIES RQ_A_Mee_B_MD.

NUMERIC RQ_A_SvD_B_MD (F2.0).

COMPUTE RQ_A_SvD_B_MD = 1.

IF (RQ_A_SvD_M_Cat3<MD_A_SvD_B) RQ_A_SvD_B_MD =2.

VARIABLE LABELS RQ_A_SvD_B_MD 'Onder mediaan Syndroom van Down, B - test'.

VALUE LABELS RQ_A_SvD_B_MD

2 'Onder mediaan'

1 'Gelijk of boven mediaan'.

FREQUENCIES RQ_A_SvD_B_MD.

NUMERIC RQ_A_GBP_B_MD (F2.0).

COMPUTE RQ_A_GBP_B_MD = 1.

IF (RQ_A_Gbp_B_M_Cat5<MD_A_GBP_B) RQ_A_GBP_B_MD =2.

VARIABLE LABELS RQ_A_GBP_B_MD 'Onder mediaan Geboorteplan, B - test'.

VALUE LABELS RQ_A_GBP_B_MD

2 'Onder mediaan'

1 'Gelijk of boven mediaan'.

FREQUENCIES RQ_A_GBP_B_MD.

*privacy.

NUMERIC RQ_P_Med_B_MD (F2.0).

COMPUTE RQ_P_Med_B_MD = 1.

IF (RQ_P_Med_B_M<MD_P_Med_B) RQ_P_Med_B_MD =2.

VARIABLE LABELS RQ_P_Med_B_MD 'Onder mediaan Medische dossier, B - test, hertest'.

VALUE LABELS RQ_P_Med_B_MD

2 'Onder mediaan'

1 'Gelijk of boven mediaan'.

FREQUENCIES RQ_P_Med_B_MD.

NUMERIC RQ_P_Mln_B_MD (F2.0).

COMPUTE RQ_P_Mln_B_MD = 1.

IF (RQ_P_Mln_B_M<MD_P_Mln_B) RQ_P_Mln_B_MD =2.

VARIABLE LABELS RQ_P_Mln_B_MD 'Onder mediaan Meeluisteren, B - test, hertest'.

VALUE LABELS RQ_P_Mln_B_MD

2 'Onder mediaan'

1 'Gelijk of boven mediaan'.

FREQUENCIES RQ_P_Mln_B_MD.

*communicatie.

NUMERIC RQ_C_Ant_B_MD (F2.0).

COMPUTE RQ_C_Ant_B_MD = 1.

IF (RQ_C_Ant_B_M<MD_C_Ant_B) RQ_C_Ant_B_MD =2.

VARIABLE LABELS RQ_C_Ant_B_MD 'Onder mediaan Antwoord op vragen, B - test'.

VALUE LABELS RQ_C_Ant_B_MD

2 'Onder mediaan'

1 'Gelijk of boven mediaan'.

FREQUENCIES RQ_C_Ant_B_MD.

NUMERIC RQ_C_Adv_B_MD (F2.0).

COMPUTE RQ_C_Adv_B_MD = 1.

IF (RQ_C_Adv_B_M<MD_C_Adv_B) RQ_C_Adv_B_MD =2.

VARIABLE LABELS RQ_C_Adv_B_MD 'Onder mediaan krijgen zelfde adviezen, B - test'.

VALUE LABELS RQ_C_Adv_B_MD

2 'Onder mediaan'

1 'Gelijk of boven mediaan'.

FREQUENCIES RQ_C_Adv_B_MD.

NUMERIC RQ_C_Uit_B_MD (F2.0).

COMPUTE RQ_C_Uit_B_MD = 1.

IF (RQ_C_Uit_B_M<MD_C_Uit_B) RQ_C_Uit_B_MD =2.

VARIABLE LABELS RQ_C_Uit_B_MD 'Onder mediaan Begrijpen uitleg, B - test'.

VALUE LABELS RQ_C_Uit_B_MD

2 'Onder mediaan'

1 'Gelijk of boven mediaan'.

FREQUENCIES RQ_C_Uit_B_MD.

NUMERIC RQ_C_Inf_B_MD (F2.0).

COMPUTE RQ_C_Inf_B_MD = 1.

IF (RQ_C_Inf_B_M<MD_C_Inf_B) RQ_C_Inf_B_MD =2.

VARIABLE LABELS RQ_C_Inf_B_MD 'Onder mediaan Informatie tijdens behandeling, B - test'.

VALUE LABELS RQ_C_Inf_B_MD

2 'Onder mediaan'

1 'Gelijk of boven mediaan'.

FREQUENCIES RQ_C_Inf_B_MD.

*tijd tot hulp.

NUMERIC RQ_T_Ghd_B_MD (F2.0).

COMPUTE RQ_T_Ghd_B_MD = 1.

IF (RQ_T_Ghd_B_M_Cat5<MD_T_Ghd_B) RQ_T_Ghd_B_MD =2.

VARIABLE LABELS RQ_T_Ghd_B_MD 'Onder mediaan Hulp als dringend, B - test'.

VALUE LABELS RQ_T_Ghd_B_MD

2 'Onder mediaan'

1 'Gelijk of boven mediaan'.

FREQUENCIES RQ_T_Ghd_B_MD.

NUMERIC RQ_T_Gnd_B_MD (F2.0).

COMPUTE RQ_T_Gnd_B_MD = 1.

IF (RQ_T_Gnd_B_M<MD_T_Gnd_B) RQ_T_Gnd_B_MD =2.

VARIABLE LABELS RQ_T_Gnd_B_MD 'Onder mediaan Hulp als niet dringend, B - test'.

VALUE LABELS RQ_T_Gnd_B_MD

2 'Onder mediaan'

1 'Gelijk of boven mediaan'.

FREQUENCIES RQ_T_Gnd_B_MD.

NUMERIC RQ_T_TYD_B_MD (F2.0).

COMPUTE RQ_T_TYD_B_MD = 1.

IF (RQ_T_Tyd_B_M<MD_T_Tyd_B) RQ_T_TYD_B_MD =2.

VARIABLE LABELS RQ_T_TYD_B_MD 'Onder mediaan Tijd als nodig, B - test'.

VALUE LABELS RQ_T_TYD_B_MD

2 'Onder mediaan'

1 'Gelijk of boven mediaan'.

FREQUENCIES RQ_T_TYD_B_MD.

NUMERIC RQ_T_ANW_B_MD (F2.0).

COMPUTE RQ_T_ANW_B_MD = 1.

IF (RQ_T_Anw_B_M<MD_T_Anw_B) RQ_T_ANW_B_MD =2.

VARIABLE LABELS RQ_T_ANW_B_MD 'Onder mediaan Bij afspraak snel aan de beurt, B - test'.

VALUE LABELS RQ_T_ANW_B_MD

2 'Onder mediaan'

1 'Gelijk of boven mediaan'.

FREQUENCIES RQ_T_ANW_B_MD.

NUMERIC RQ_T_BER_B_MD (F2.0).

COMPUTE RQ_T_BER_B_MD = 1.

IF (RQ_T_Ber_B_M<MD_T_Ber_B) RQ_T_BER_B_MD =2.

VARIABLE LABELS RQ_T_BER_B_MD 'Onder mediaan bereikbaarheid locatie, B - test'.

VALUE LABELS RQ_T_BER_B_MD

2 'Onder mediaan'

1 'Gelijk of boven mediaan'.

FREQUENCIES RQ_T_BER_B_MD.

NUMERIC RQ_T_TEL_B_MD (F2.0).

COMPUTE RQ_T_TEL_B_MD = 1.

IF (RQ_T_Tel_B_M<MD_T_Tel_B) RQ_T_TEL_B_MD =2.

VARIABLE LABELS RQ_T_TEL_B_MD 'Onder mediaan telefonische bereikbaarheid, B - test'.

VALUE LABELS RQ_T_TEL_B_MD

2 'Onder mediaan'

1 'Gelijk of boven mediaan'.

FREQUENCIES RQ_T_TEL_B_MD.

*Sociale ondersteuning.

NUMERIC RQ_S_Fam_B_MD (F2.0).

COMPUTE RQ_S_Fam_B_MD = 1.

IF (RQ_S_Fam_B_M_Cat5<MD_S_Fam_B) RQ_S_Fam_B_MD =2.

VARIABLE LABELS RQ_S_Fam_B_MD 'Onder mediaan Betrekken familie, B - test'.

VALUE LABELS RQ_S_Fam_B_MD

2 'Onder mediaan'

1 'Gelijk of boven mediaan'.

FREQUENCIES RQ_S_Fam_B_MD.

NUMERIC RQ_S_Rhm_B_MD (F2.0).

COMPUTE RQ_S_Rhm_B_MD = 1.

IF (RQ_S_Rhm_B_M<MD_S_Rhm_B) RQ_S_Rhm_B_MD =2.

VARIABLE LABELS RQ_S_Rhm_B_MD 'Onder mediaan Rekening houden met gezin, B - test'.

VALUE LABELS RQ_S_Rhm_B_MD

2 'Onder mediaan'

1 'Gelijk of boven mediaan'.

FREQUENCIES RQ_S_Rhm_B_MD.

NUMERIC RQ_S_Ste_B_MD (F2.0).

COMPUTE RQ_S_Ste_B_MD = 1.

IF (RQ_S_Ste_B_M<MD_S_Ste_B) RQ_S_Ste_B_MD =2.

VARIABLE LABELS RQ_S_Ste_B_MD 'Onder mediaan Gesteund voelen, B - test'.

VALUE LABELS RQ_S_Ste_B_MD

2 'Onder mediaan'

1 'Gelijk of boven mediaan'.

FREQUENCIES RQ_S_Ste_B_MD.

*Faciliteiten.

NUMERIC RQ_F_Cmf_B_MD (F2.0).

COMPUTE RQ_F_Cmf_B_MD = 1.

IF (RQ_F_Cmf_B_M<MD_F_Cmf_B) RQ_F_Cmf_B_MD =2.

VARIABLE LABELS RQ_F_Cmf_B_MD 'Onder mediaan Comfort, B - test'.

VALUE LABELS RQ_F_Cmf_B_MD

2 'Onder mediaan'

1 'Gelijk of boven mediaan'.

FREQUENCIES RQ_F_Cmf_B_MD.

NUMERIC RQ_F_Hyg_B_MD (F2.0).

COMPUTE RQ_F_Hyg_B_MD = 1.

IF (RQ_F_Hyg_B_M<MD_F_Hyg_B) RQ_F_Hyg_B_MD =2.

VARIABLE LABELS RQ_F_Hyg_B_MD 'Onder mediaan Hygiene, B - test'.

VALUE LABELS RQ_F_Hyg_B_MD

2 'Onder mediaan'

1 'Gelijk of boven mediaan'.

FREQUENCIES RQ_F_Hyg_B_MD.

NUMERIC RQ_F_Toe_B_MD (F2.0).

COMPUTE RQ_F_Toe_B_MD = 1.

IF (RQ_F_Toe_B_M<MD_F_Toe_B) RQ_F_Toe_B_MD =2.

VARIABLE LABELS RQ_F_Toe_B_MD 'Onder mediaan Toegankelijkheid ruimtes, B - test'.

VALUE LABELS RQ_F_Toe_B_MD

2 'Onder mediaan'

1 'Gelijk of boven mediaan'.

FREQUENCIES RQ_F_Toe_B_MD.

*Keuze en continuiteit.

NUMERIC RQ_K_Wis_B_MD (F2.0).

COMPUTE RQ_K_Wis_B_MD = 1.

IF (RQ_K_Wis_B_M_Cat4<MD_K_Wis_B) RQ_K_Wis_B_MD =2.

VARIABLE LABELS RQ_K_Wis_B_MD 'Onder mediaan Wisselen zorgverlener, B - test'.

VALUE LABELS RQ_K_Wis_B_MD

2 'Onder mediaan'

1 'Gelijk of boven mediaan'.

FREQUENCIES RQ_K_Wis_B_MD.

NUMERIC RQ_K_Vwz_B_MD (F2.0).

COMPUTE RQ_K_Vwz_B_MD = 1.

IF (RQ_K_Vwz_B_M_Cat5<MD_K_Vwz_B) RQ_K_Vwz_B_MD =2.

VARIABLE LABELS RQ_K_Vwz_B_MD 'Onder mediaan Verwijzing naar het ziekenhuis, B - test'.

VALUE LABELS RQ_K_Vwz_B_MD

2 'Onder mediaan'

1 'Gelijk of boven mediaan'.

FREQUENCIES RQ_K_Vwz_B_MD.

NUMERIC RQ_K_Soo_B_MD (F2.0).

COMPUTE RQ_K_Soo_B_MD = 1.

IF (RQ_K_Soo_B_M<MD_K_Soo_B) RQ_K_Soo_B_MD =2.

VARIABLE LABELS RQ_K_Soo_B_MD 'Onder mediaan Keuze soort zorgverlener, B - test'.

VALUE LABELS RQ_K_Soo_B_MD

2 'Onder mediaan'

1 'Gelijk of boven mediaan'.

FREQUENCIES RQ_K_Soo_B_MD.

NUMERIC RQ_K_Lei_B_MD (F2.0).

COMPUTE RQ_K_Lei_B_MD = 1.

IF (RQ_K_Lei_B_M<MD_K_Lei_B) RQ_K_Lei_B_MD =2.

VARIABLE LABELS RQ_K_Lei_B_MD 'Onder mediaan Leiding zorg, B - test'.

VALUE LABELS RQ_K_Lei_B_MD

2 'Onder mediaan'

1 'Gelijk of boven mediaan'.

FREQUENCIES RQ_K_Lei_B_MD.

* STAP 1: berekenen percentage Onder mediaan op item niveau hertest.

*respect.

NUMERIC RQ_R_Pri_B_RE_MD (F2.0).

COMPUTE RQ_R_Pri_B_RE_MD = 1.

IF (RQ_R_Pri_B_RE_M<MD_R_Pri_B) RQ_R_Pri_B_RE_MD =2.

VARIABLE LABELS RQ_R_Pri_B_RE_MD 'Onder mediaan Rekening houden met privacy, B - hertest'.

VALUE LABELS RQ_R_Pri_B_RE_MD

2 'Onder mediaan'

1 'Gelijk of boven mediaan'.

FREQUENCIES RQ_R_Pri_B_RE_MD.

NUMERIC RQ_R_RES_B_RE_MD (F2.0).

COMPUTE RQ_R_RES_B_RE_MD = 1.

IF (RQ_R_Res_B_RE_M<MD_R_Res_B) RQ_R_Res_B_RE_MD =2.

VARIABLE LABELS RQ_R_RES_B_RE_MD 'Onder mediaan Behandeld met respect, B - hertest'.

VALUE LABELS RQ_R_RES_B_RE_MD

2 'Onder mediaan'

1 'Gelijk of boven mediaan'.

FREQUENCIES RQ_R_RES_B_RE_MD.

NUMERIC RQ_R_Per_B_RE_MD (F2.0).

COMPUTE RQ_R_Per_B_RE_MD = 1.

IF (RQ_R_Per_B_RE_M<MD_R_Per_B) RQ_R_Per_B_RE_MD =2.

VARIABLE LABELS RQ_R_Per_B_RE_MD 'Onder mediaan Persoonlijke aandacht, B - hertest'.

VALUE LABELS RQ_R_Per_B_RE_MD

2 'Onder mediaan'

1 'Gelijk of boven mediaan'.

FREQUENCIES RQ_R_Per_B_RE_MD.

NUMERIC RQ_R_Vri_B_RE_MD (F2.0).

COMPUTE RQ_R_Vri_B_RE_MD = 1.

IF (RQ_R_Vri_B_RE_M<MD_R_Vri_B) RQ_R_Vri_B_RE_MD =2.

VARIABLE LABELS RQ_R_Vri_B_RE_MD 'Onder mediaan Vriendelijk behandeld, B - hertest'.

VALUE LABELS RQ_R_Vri_B_RE_MD

2 'Onder mediaan'

1 'Gelijk of boven mediaan'.

FREQUENCIES RQ_R_Vri_B_RE_MD.

NUMERIC RQ_R_Wen_B_RE_MD (F2.0).

COMPUTE RQ_R_Wen_B_RE_MD = 1.

IF (RQ_R_Wen_B_RE_M<MD_R_Wen_B) RQ_R_Wen_B_RE_MD =2.

VARIABLE LABELS RQ_R_Wen_B_RE_MD 'Onder mediaan Rekening houden met wensen en behoeften, B - hertest'.

VALUE LABELS RQ_R_Wen_B_RE_MD

2 'Onder mediaan'

1 'Gelijk of boven mediaan'.

FREQUENCIES RQ_R_Wen_B_RE_MD.

NUMERIC RQ_R_Ver_B_RE_MD (F2.0).

COMPUTE RQ_R_Ver_B_RE_MD = 1.

IF (RQ_R_Ver_B_RE_M<MD_R_Ver_B) RQ_R_Ver_B_RE_MD =2.

VARIABLE LABELS RQ_R_Ver_B_RE_MD 'Onder mediaan Alles vertellen aan zorgverlener, B - hertest'.

VALUE LABELS RQ_R_Ver_B_RE_MD

2 'Onder mediaan'

1 'Gelijk of boven mediaan'.

FREQUENCIES RQ_R_Ver_B_RE_MD.

*autonomie.

NUMERIC RQ_A_Wei_B_RE_MD (F2.0).

COMPUTE RQ_A_Wei_B_RE_MD = 1.

IF (RQ_A_Wei_B_RE_M<MD_A_Wei_B) RQ_A_Wei_B_RE_MD =2.

VARIABLE LABELS RQ_A_Wei_B_RE_MD 'Onder mediaan Weigeren behandeling, B - hertest'.

VALUE LABELS RQ_A_Wei_B_RE_MD

2 'Onder mediaan'

1 'Gelijk of boven mediaan'.

FREQUENCIES RQ_A_Wei_B_RE_MD.

NUMERIC RQ_A_Mee_B_RE_MD (F2.0).

COMPUTE RQ_A_Mee_B_RE_MD = 1.

IF (RQ_A_Mee_B_RE_M<MD_A_Mee_B) RQ_A_Mee_B_RE_MD =2.

VARIABLE LABELS RQ_A_Mee_B_RE_MD 'Onder mediaan Meebeslissen, B - hertest'.

VALUE LABELS RQ_A_Mee_B_RE_MD

2 'Onder mediaan'

1 'Gelijk of boven mediaan'.

FREQUENCIES RQ_A_Mee_B_RE_MD.

NUMERIC RQ_A_SvD_B_RE_MD (F2.0).

COMPUTE RQ_A_SvD_B_RE_MD = 1.

IF (RQ_A_SvD_Re_M_Cat3<MD_A_SvD_B) RQ_A_SvD_B_RE_MD =2.

VARIABLE LABELS RQ_A_SvD_B_RE_MD 'Onder mediaan Syndroom van Down, B - hertest'.

VALUE LABELS RQ_A_SvD_B_RE_MD

2 'Onder mediaan'

1 'Gelijk of boven mediaan'.

FREQUENCIES RQ_A_SvD_B_RE_MD.

NUMERIC RQ_A_GBP_B_RE_MD (F2.0).

COMPUTE RQ_A_GBP_B_RE_MD = 1.

IF (RQ_A_Gbp_RE_M_Cat5<MD_A_GBP_B) RQ_A_GBP_B_RE_MD =2.

VARIABLE LABELS RQ_A_GBP_B_RE_MD 'Onder mediaan Geboorteplan, B - hertest'.

VALUE LABELS RQ_A_GBP_B_RE_MD

2 'Onder mediaan'

1 'Gelijk of boven mediaan'.

FREQUENCIES RQ_A_GBP_B_RE_MD.

*privacy.

NUMERIC RQ_P_Med_B_RE_MD (F2.0).

COMPUTE RQ_P_Med_B_RE_MD = 1.

IF (RQ_P_Med_B_RE_M<MD_P_Med_B) RQ_P_Med_B_RE_MD =2.

VARIABLE LABELS RQ_P_Med_B_RE_MD 'Onder mediaan Medische dossier, B - hertest, herhertest'.

VALUE LABELS RQ_P_Med_B_RE_MD

2 'Onder mediaan'

1 'Gelijk of boven mediaan'.

FREQUENCIES RQ_P_Med_B_RE_MD.

NUMERIC RQ_P_Mln_B_RE_MD (F2.0).

COMPUTE RQ_P_Mln_B_RE_MD = 1.

IF (RQ_P_Mln_B_RE_M<MD_P_Mln_B) RQ_P_Mln_B_RE_MD =2.

VARIABLE LABELS RQ_P_Mln_B_RE_MD 'Onder mediaan Meeluisteren, B - hertest, herhertest'.

VALUE LABELS RQ_P_Mln_B_RE_MD

2 'Onder mediaan'

1 'Gelijk of boven mediaan'.

FREQUENCIES RQ_P_Mln_B_RE_MD.

*communicatie.

NUMERIC RQ_C_Ant_B_RE_MD (F2.0).

COMPUTE RQ_C_Ant_B_RE_MD = 1.

IF (RQ_C_Ant_B_RE_M<MD_C_Ant_B) RQ_C_Ant_B_RE_MD =2.

VARIABLE LABELS RQ_C_Ant_B_RE_MD 'Onder mediaan Antwoord op vragen, B - hertest'.

VALUE LABELS RQ_C_Ant_B_RE_MD

2 'Onder mediaan'

1 'Gelijk of boven mediaan'.

FREQUENCIES RQ_C_Ant_B_RE_MD.

NUMERIC RQ_C_Adv_B_RE_MD (F2.0).

COMPUTE RQ_C_Adv_B_RE_MD = 1.

IF (RQ_C_Adv_B_RE_M<MD_C_Adv_B) RQ_C_Adv_B_RE_MD =2.

VARIABLE LABELS RQ_C_Adv_B_RE_MD 'Onder mediaan krijgen zelfde adviezen, B - hertest'.

VALUE LABELS RQ_C_Adv_B_RE_MD

2 'Onder mediaan'

1 'Gelijk of boven mediaan'.

FREQUENCIES RQ_C_Adv_B_RE_MD.

NUMERIC RQ_C_Uit_B_RE_MD (F2.0).

COMPUTE RQ_C_Uit_B_RE_MD = 1.

IF (RQ_C_Uit_B_RE_M<MD_C_Uit_B) RQ_C_Uit_B_RE_MD =2.

VARIABLE LABELS RQ_C_Uit_B_RE_MD 'Onder mediaan Begrijpen uitleg, B - hertest'.

VALUE LABELS RQ_C_Uit_B_RE_MD

2 'Onder mediaan'

1 'Gelijk of boven mediaan'.

FREQUENCIES RQ_C_Uit_B_RE_MD.

NUMERIC RQ_C_Inf_B_RE_MD (F2.0).

COMPUTE RQ_C_Inf_B_RE_MD = 1.

IF (RQ_C_Inf_B_RE_M<MD_C_Inf_B) RQ_C_Inf_B_RE_MD =2.

VARIABLE LABELS RQ_C_Inf_B_RE_MD 'Onder mediaan Informatie tijdens behandeling, B - hertest'.

VALUE LABELS RQ_C_Inf_B_RE_MD

2 'Onder mediaan'

1 'Gelijk of boven mediaan'.

FREQUENCIES RQ_C_Inf_B_RE_MD.

*tijd tot hulp.

NUMERIC RQ_T_Ghd_B_RE_MD (F2.0).

COMPUTE RQ_T_Ghd_B_RE_MD = 1.

IF (RQ_T_Ghd_B_RE_M_Cat5<MD_T_Ghd_B) RQ_T_Ghd_B_RE_MD =2.

VARIABLE LABELS RQ_T_Ghd_B_RE_MD 'Onder mediaan Hulp als dringend, B - hertest'.

VALUE LABELS RQ_T_Ghd_B_RE_MD

2 'Onder mediaan'

1 'Gelijk of boven mediaan'.

FREQUENCIES RQ_T_Ghd_B_RE_MD.

NUMERIC RQ_T_Gnd_B_RE_MD (F2.0).

COMPUTE RQ_T_Gnd_B_RE_MD = 1.

IF (RQ_T_Gnd_B_RE_M<MD_T_Gnd_B) RQ_T_Gnd_B_RE_MD =2.

VARIABLE LABELS RQ_T_Gnd_B_RE_MD 'Onder mediaan Hulp als niet dringend, B - hertest'.

VALUE LABELS RQ_T_Gnd_B_RE_MD

2 'Onder mediaan'

1 'Gelijk of boven mediaan'.

FREQUENCIES RQ_T_Gnd_B_RE_MD.

NUMERIC RQ_T_TYD_B_RE_MD (F2.0).

COMPUTE RQ_T_TYD_B_RE_MD = 1.

IF (RQ_T_Tyd_B_RE_M<MD_T_Tyd_B) RQ_T_TYD_B_RE_MD =2.

VARIABLE LABELS RQ_T_TYD_B_RE_MD 'Onder mediaan Tijd als nodig, B - hertest'.

VALUE LABELS RQ_T_TYD_B_RE_MD

2 'Onder mediaan'

1 'Gelijk of boven mediaan'.

FREQUENCIES RQ_T_TYD_B_RE_MD.

NUMERIC RQ_T_ANW_B_RE_MD (F2.0).

COMPUTE RQ_T_ANW_B_RE_MD = 1.

IF (RQ_T_Anw_B_RE_M<MD_T_Anw_B) RQ_T_ANW_B_RE_MD =2.

VARIABLE LABELS RQ_T_ANW_B_RE_MD 'Onder mediaan Bij afspraak snel aan de beurt, B - hertest'.

VALUE LABELS RQ_T_ANW_B_RE_MD

2 'Onder mediaan'

1 'Gelijk of boven mediaan'.

FREQUENCIES RQ_T_ANW_B_RE_MD.

NUMERIC RQ_T_BER_B_RE_MD (F2.0).

COMPUTE RQ_T_BER_B_RE_MD = 1.

IF (RQ_T_Ber_B_RE_M<MD_T_Ber_B) RQ_T_BER_B_RE_MD =2.

VARIABLE LABELS RQ_T_BER_B_RE_MD 'Onder mediaan bereikbaarheid locatie, B - hertest'.

VALUE LABELS RQ_T_BER_B_RE_MD

2 'Onder mediaan'

1 'Gelijk of boven mediaan'.

FREQUENCIES RQ_T_BER_B_RE_MD.

NUMERIC RQ_T_TEL_B_RE_MD (F2.0).

COMPUTE RQ_T_TEL_B_RE_MD = 1.

IF (RQ_T_Tel_B_RE_M<MD_T_Tel_B) RQ_T_TEL_B_RE_MD =2.

VARIABLE LABELS RQ_T_TEL_B_RE_MD 'Onder mediaan telefonische bereikbaarheid, B - hertest'.

VALUE LABELS RQ_T_TEL_B_RE_MD

2 'Onder mediaan'

1 'Gelijk of boven mediaan'.

FREQUENCIES RQ_T_TEL_B_RE_MD.

*Sociale ondersteuning.

NUMERIC RQ_S_Fam_B_RE_MD (F2.0).

COMPUTE RQ_S_Fam_B_RE_MD = 1.

IF (RQ_S_Fam_B_RE_M_Cat5<MD_S_Fam_B) RQ_S_Fam_B_RE_MD =2.

VARIABLE LABELS RQ_S_Fam_B_RE_MD 'Onder mediaan Betrekken familie, B - hertest'.

VALUE LABELS RQ_S_Fam_B_RE_MD

2 'Onder mediaan'

1 'Gelijk of boven mediaan'.

FREQUENCIES RQ_S_Fam_B_RE_MD.

NUMERIC RQ_S_Rhm_B_RE_MD (F2.0).

COMPUTE RQ_S_Rhm_B_RE_MD = 1.

IF (RQ_S_Rhm_B_RE_M<MD_S_Rhm_B) RQ_S_Rhm_B_RE_MD =2.

VARIABLE LABELS RQ_S_Rhm_B_RE_MD 'Onder mediaan Rekening houden met gezin, B - hertest'.

VALUE LABELS RQ_S_Rhm_B_RE_MD

2 'Onder mediaan'

1 'Gelijk of boven mediaan'.

FREQUENCIES RQ_S_Rhm_B_RE_MD.

NUMERIC RQ_S_Ste_B_RE_MD (F2.0).

COMPUTE RQ_S_Ste_B_RE_MD = 1.

IF (RQ_S_Ste_B_RE_M<MD_S_Ste_B) RQ_S_Ste_B_RE_MD =2.

VARIABLE LABELS RQ_S_Ste_B_RE_MD 'Onder mediaan Gesteund voelen, B - hertest'.

VALUE LABELS RQ_S_Ste_B_RE_MD

2 'Onder mediaan'

1 'Gelijk of boven mediaan'.

FREQUENCIES RQ_S_Ste_B_RE_MD.

*Faciliteiten.

NUMERIC RQ_F_Cmf_B_RE_MD (F2.0).

COMPUTE RQ_F_Cmf_B_RE_MD = 1.

IF (RQ_F_Cmf_B_RE_M<MD_F_Cmf_B) RQ_F_Cmf_B_RE_MD =2.

VARIABLE LABELS RQ_F_Cmf_B_RE_MD 'Onder mediaan Comfort, B - hertest'.

VALUE LABELS RQ_F_Cmf_B_RE_MD

2 'Onder mediaan'

1 'Gelijk of boven mediaan'.

FREQUENCIES RQ_F_Cmf_B_RE_MD.

NUMERIC RQ_F_Hyg_B_RE_MD (F2.0).

COMPUTE RQ_F_Hyg_B_RE_MD = 1.

IF (RQ_F_Hyg_B_RE_M<MD_F_Hyg_B) RQ_F_Hyg_B_RE_MD =2.

VARIABLE LABELS RQ_F_Hyg_B_RE_MD 'Onder mediaan Hygiene, B - hertest'.

VALUE LABELS RQ_F_Hyg_B_RE_MD

2 'Onder mediaan'

1 'Gelijk of boven mediaan'.

FREQUENCIES RQ_F_Hyg_B_RE_MD.

NUMERIC RQ_F_Toe_B_RE_MD (F2.0).

COMPUTE RQ_F_Toe_B_RE_MD = 1.

IF (RQ_F_Toe_B_RE_M<MD_F_Toe_B) RQ_F_Toe_B_RE_MD =2.

VARIABLE LABELS RQ_F_Toe_B_RE_MD 'Onder mediaan Toegankelijkheid ruimtes, B - hertest'.

VALUE LABELS RQ_F_Toe_B_RE_MD

2 'Onder mediaan'

1 'Gelijk of boven mediaan'.

FREQUENCIES RQ_F_Toe_B_RE_MD.

*Keuze en continuiteit.

NUMERIC RQ_K_Wis_B_RE_MD (F2.0).

COMPUTE RQ_K_Wis_B_RE_MD = 1.

IF (RQ_K_Wis_B_RE_M_Cat4<MD_K_Wis_B) RQ_K_Wis_B_RE_MD =2.

VARIABLE LABELS RQ_K_Wis_B_RE_MD 'Onder mediaan Wisselen zorgverlener, B - hertest'.

VALUE LABELS RQ_K_Wis_B_RE_MD

2 'Onder mediaan'

1 'Gelijk of boven mediaan'.

FREQUENCIES RQ_K_Wis_B_RE_MD.

NUMERIC RQ_K_Vwz_B_RE_MD (F2.0).

COMPUTE RQ_K_Vwz_B_RE_MD = 1.

IF (RQ_K_Vwz_B_RE_M_Cat5<MD_K_Vwz_B) RQ_K_Vwz_B_RE_MD =2.

VARIABLE LABELS RQ_K_Vwz_B_RE_MD 'Onder mediaan Verwijzing naar het ziekenhuis, B - hertest'.

VALUE LABELS RQ_K_Vwz_B_RE_MD

2 'Onder mediaan'

1 'Gelijk of boven mediaan'.

FREQUENCIES RQ_K_Vwz_B_RE_MD.

NUMERIC RQ_K_Soo_B_RE_MD (F2.0).

COMPUTE RQ_K_Soo_B_RE_MD = 1.

IF (RQ_K_Soo_B_RE_M<MD_K_Soo_B) RQ_K_Soo_B_RE_MD =2.

VARIABLE LABELS RQ_K_Soo_B_RE_MD 'Onder mediaan Keuze soort zorgverlener, B - hertest'.

VALUE LABELS RQ_K_Soo_B_RE_MD

2 'Onder mediaan'

1 'Gelijk of boven mediaan'.

FREQUENCIES RQ_K_Soo_B_RE_MD.

NUMERIC RQ_K_Lei_B_RE_MD (F2.0).

COMPUTE RQ_K_Lei_B_RE_MD = 1.

IF (RQ_K_Lei_B_RE_M<MD_K_Lei_B) RQ_K_Lei_B_RE_MD =2.

VARIABLE LABELS RQ_K_Lei_B_RE_MD 'Onder mediaan Leiding zorg, B - hertest'.

VALUE LABELS RQ_K_Lei_B_RE_MD

2 'Onder mediaan'

1 'Gelijk of boven mediaan'.

FREQUENCIES RQ_K_Lei_B_RE_MD.

*

*STAP 3:Berekenen absolute agreement % Mediaan TEST-HERTEST.

*Respect.

NUMERIC RQ_R_Pri_B_MD_AA_C3 (F2).

COMPUTE RQ_R_Pri_B_MD_AA_C3 = $SYSMIS.

IF (RQ_R_Pri_B_MD=RQ_R_Pri_B_RE_MD) RQ_R_Pri_B_MD_AA_C3 =1.

IF (RQ_R_Pri_B_MD>RQ_R_Pri_B_RE_MD) RQ_R_Pri_B_MD_AA_C3 =3.

IF (RQ_R_Pri_B_MD<RQ_R_Pri_B_RE_MD) RQ_R_Pri_B_MD_AA_C3 =2.

VARIABLE LABELS RQ_R_Pri_B_MD_AA_C3 'Absoluut agreement voor "Rekening houden privacy" test-thentest - MD, C3' .

VALUE LABELS RQ_R_Pri_B_MD_AA_C3

1 'test-thentest gelijk'

2 'test positiever dan hertest'

3 'test negatiever dan hertest' .

FREQUENCIES RQ_R_Pri_B_MD_AA_C3.

NUMERIC RQ_R_RES_B_MD_AA_C3 (F2).

COMPUTE RQ_R_RES_B_MD_AA_C3 = $SYSMIS.

IF (RQ_R_RES_B_MD=RQ_R_RES_B_RE_MD) RQ_R_RES_B_MD_AA_C3 =1.

IF (RQ_R_RES_B_MD>RQ_R_RES_B_RE_MD) RQ_R_RES_B_MD_AA_C3 =3.

IF (RQ_R_RES_B_MD<RQ_R_RES_B_RE_MD) RQ_R_RES_B_MD_AA_C3 =2.

VARIABLE LABELS RQ_R_RES_B_MD_AA_C3 'Absoluut agreement voor "Behandeld met respect" test-thentest - MD, C3' .

VALUE LABELS RQ_R_RES_B_MD_AA_C3

1 'test-thentest gelijk'

2 'test positiever dan hertest'

3 'test negatiever dan hertest' .

FREQUENCIES RQ_R_RES_B_MD_AA_C3.

NUMERIC RQ_R_Per_B_MD_AA_C3 (F2).

COMPUTE RQ_R_Per_B_MD_AA_C3 = $SYSMIS.

IF (RQ_R_Per_B_MD=RQ_R_Per_B_RE_MD) RQ_R_Per_B_MD_AA_C3 =1.

IF (RQ_R_Per_B_MD>RQ_R_Per_B_RE_MD) RQ_R_Per_B_MD_AA_C3 =3.

IF (RQ_R_Per_B_MD<RQ_R_Per_B_RE_MD) RQ_R_Per_B_MD_AA_C3 =2.

VARIABLE LABELS RQ_R_Per_B_MD_AA_C3 'Absoluut agreement voor "Persoonlijke aandacht" test-thentest - MD, C3' .

VALUE LABELS RQ_R_Per_B_MD_AA_C3

1 'test-thentest gelijk'

2 'test positiever dan hertest'

3 'test negatiever dan hertest' .

FREQUENCIES RQ_R_Per_B_MD_AA_C3.

NUMERIC RQ_R_Vri_B_MD_AA_C3 (F2).

COMPUTE RQ_R_Vri_B_MD_AA_C3 = $SYSMIS.

IF (RQ_R_Vri_B_MD=RQ_R_Vri_B_RE_MD) RQ_R_Vri_B_MD_AA_C3 =1.

IF (RQ_R_Vri_B_MD>RQ_R_Vri_B_RE_MD) RQ_R_Vri_B_MD_AA_C3 =3.

IF (RQ_R_Vri_B_MD<RQ_R_Vri_B_RE_MD) RQ_R_Vri_B_MD_AA_C3 =2.

VARIABLE LABELS RQ_R_Vri_B_MD_AA_C3 'Absoluut agreement voor "Vriendelijk behandeld" test-thentest - MD, C3' .

VALUE LABELS RQ_R_Vri_B_MD_AA_C3

1 'test-thentest gelijk'

2 'test positiever dan hertest'

3 'test negatiever dan hertest' .

FREQUENCIES RQ_R_Vri_B_MD_AA_C3.

NUMERIC RQ_R_Wen_B_MD_AA_C3 (F2).

COMPUTE RQ_R_Wen_B_MD_AA_C3 = $SYSMIS.

IF (RQ_R_Wen_B_MD=RQ_R_Wen_B_RE_MD) RQ_R_Wen_B_MD_AA_C3 =1.

IF (RQ_R_Wen_B_MD>RQ_R_Wen_B_RE_MD) RQ_R_Wen_B_MD_AA_C3 =3.

IF (RQ_R_Wen_B_MD<RQ_R_Wen_B_RE_MD) RQ_R_Wen_B_MD_AA_C3 =2.

VARIABLE LABELS RQ_R_Wen_B_MD_AA_C3 'Absoluut agreement voor "Wensen en behoeften" test-thentest - MD, C3' .

VALUE LABELS RQ_R_Wen_B_MD_AA_C3

1 'test-thentest gelijk'

2 'test positiever dan hertest'

3 'test negatiever dan hertest' .

FREQUENCIES RQ_R_Wen_B_MD_AA_C3.

NUMERIC RQ_R_Ver_B_MD_AA_C3 (F2).

COMPUTE RQ_R_Ver_B_MD_AA_C3 = $SYSMIS.

IF (RQ_R_Ver_B_MD=RQ_R_Ver_B_RE_MD) RQ_R_Ver_B_MD_AA_C3 =1.

IF (RQ_R_Ver_B_MD>RQ_R_Ver_B_RE_MD) RQ_R_Ver_B_MD_AA_C3 =3.

IF (RQ_R_Ver_B_MD<RQ_R_Ver_B_RE_MD) RQ_R_Ver_B_MD_AA_C3 =2.

VARIABLE LABELS RQ_R_Ver_B_MD_AA_C3 'Absoluut agreement voor "Vertrouwen" test-thentest - MD, C3' .

VALUE LABELS RQ_R_Ver_B_MD_AA_C3

1 'test-thentest gelijk'

2 'test positiever dan hertest'

3 'test negatiever dan hertest' .

FREQUENCIES RQ_R_Ver_B_MD_AA_C3.

*autonomie.

NUMERIC RQ_A_Wei_B_MD_AA_C3 (F2).

COMPUTE RQ_A_Wei_B_MD_AA_C3 = $SYSMIS.

IF (RQ_A_Wei_B_MD=RQ_A_Wei_B_RE_MD) RQ_A_Wei_B_MD_AA_C3 =1.

IF (RQ_A_Wei_B_MD>RQ_A_Wei_B_RE_MD) RQ_A_Wei_B_MD_AA_C3 =3.

IF (RQ_A_Wei_B_MD<RQ_A_Wei_B_RE_MD) RQ_A_Wei_B_MD_AA_C3 =2.

VARIABLE LABELS RQ_A_Wei_B_MD_AA_C3 'Absoluut agreement voor "weigeren behandeling" test-thentest - MD, C3' .

VALUE LABELS RQ_A_Wei_B_MD_AA_C3

1 'test-thentest gelijk'

2 'test positiever dan hertest'

3 'test negatiever dan hertest' .

FREQUENCIES RQ_A_Wei_B_MD_AA_C3.

NUMERIC RQ_A_Mee_B_MD_AA_C3 (F2).

COMPUTE RQ_A_Mee_B_MD_AA_C3 = $SYSMIS.

IF (RQ_A_Mee_B_MD=RQ_A_Mee_B_RE_MD) RQ_A_Mee_B_MD_AA_C3 =1.

IF (RQ_A_Mee_B_MD>RQ_A_Mee_B_RE_MD) RQ_A_Mee_B_MD_AA_C3 =3.

IF (RQ_A_Mee_B_MD<RQ_A_Mee_B_RE_MD) RQ_A_Mee_B_MD_AA_C3 =2.

VARIABLE LABELS RQ_A_Mee_B_MD_AA_C3 'Absoluut agreement voor "Meebeslissen behandeling" test-thentest - MD, C3' .

VALUE LABELS RQ_A_Mee_B_MD_AA_C3

1 'test-thentest gelijk'

2 'test positiever dan hertest'

3 'test negatiever dan hertest' .

FREQUENCIES RQ_A_Mee_B_MD_AA_C3.

NUMERIC RQ_A_SvD_B_MD_AA_C3 (F2).

COMPUTE RQ_A_SvD_B_MD_AA_C3 = $SYSMIS.

IF (RQ_A_SvD_B_MD=RQ_A_SvD_B_RE_MD) RQ_A_SvD_B_MD_AA_C3 =1.

IF (RQ_A_SvD_B_MD>RQ_A_SvD_B_RE_MD) RQ_A_SvD_B_MD_AA_C3 =3.

IF (RQ_A_SvD_B_MD<RQ_A_SvD_B_RE_MD) RQ_A_SvD_B_MD_AA_C3 =2.

VARIABLE LABELS RQ_A_SvD_B_MD_AA_C3 'Absoluut agreement voor "Syndroom van down" test-thentest - MD, C3' .

VALUE LABELS RQ_A_SvD_B_MD_AA_C3

1 'test-thentest gelijk'

2 'test positiever dan hertest'

3 'test negatiever dan hertest' .

FREQUENCIES RQ_A_SvD_B_MD_AA_C3.

NUMERIC RQ_A_Gbp_B_MD_AA_C3 (F2).

COMPUTE RQ_A_Gbp_B_MD_AA_C3 = $SYSMIS.

IF (RQ_A_GBP_B_MD=RQ_A_Gbp_B_RE_MD) RQ_A_Gbp_B_MD_AA_C3 =1.

IF (RQ_A_GBP_B_MD>RQ_A_Gbp_B_RE_MD) RQ_A_Gbp_B_MD_AA_C3 =3.

IF (RQ_A_GBP_B_MD<RQ_A_Gbp_B_RE_MD) RQ_A_Gbp_B_MD_AA_C3 =2.

VARIABLE LABELS RQ_A_Gbp_B_MD_AA_C3 'Absoluut agreement voor "Geboorteplan" test-thentest - MD, C3' .

VALUE LABELS RQ_A_Gbp_B_MD_AA_C3

1 'test-thentest gelijk'

2 'test positiever dan hertest'

3 'test negatiever dan hertest' .

FREQUENCIES RQ_A_Gbp_B_MD_AA_C3.

*Privacy.

NUMERIC RQ_P_Med_B_MD_AA_C3 (F2).

COMPUTE RQ_P_Med_B_MD_AA_C3 = $SYSMIS.

IF (RQ_P_Med_B_MD=RQ_P_Med_B_RE_MD) RQ_P_Med_B_MD_AA_C3 =1.

IF (RQ_P_Med_B_MD>RQ_P_Med_B_RE_MD) RQ_P_Med_B_MD_AA_C3 =3.

IF (RQ_P_Med_B_MD<RQ_P_Med_B_RE_MD) RQ_P_Med_B_MD_AA_C3 =2.

VARIABLE LABELS RQ_P_Med_B_MD_AA_C3 'Absoluut agreement voor "Medisch dossier" test-thentest - MD, C3' .

VALUE LABELS RQ_P_Med_B_MD_AA_C3

1 'test-thentest gelijk'

2 'test positiever dan hertest'

3 'test negatiever dan hertest' .

FREQUENCIES RQ_P_Med_B_MD_AA_C3.

NUMERIC RQ_P_MLN_B_MD_AA_C3 (F2).

COMPUTE RQ_P_MLN_B_MD_AA_C3 = $SYSMIS.

IF (RQ_P_Mln_B_MD=RQ_P_Mln_B_RE_MD) RQ_P_MLN_B_MD_AA_C3 =1.

IF (RQ_P_Mln_B_MD>RQ_P_Mln_B_RE_MD) RQ_P_MLN_B_MD_AA_C3 =3.

IF (RQ_P_Mln_B_MD<RQ_P_Mln_B_RE_MD) RQ_P_MLN_B_MD_AA_C3 =2.

VARIABLE LABELS RQ_P_MLN_B_MD_AA_C3 'Absoluut agreement voor "Meeluisteren" test-thentest - MD, C3' .

VALUE LABELS RQ_P_MLN_B_MD_AA_C3

1 'test-thentest gelijk'

2 'test positiever dan hertest'

3 'test negatiever dan hertest' .

FREQUENCIES RQ_P_MLN_B_MD_AA_C3.

*Communicatie.

NUMERIC RQ_C_Ant_B_MD_AA_C3 (F2).

COMPUTE RQ_C_Ant_B_MD_AA_C3 = $SYSMIS.

IF (RQ_C_Ant_B_MD=RQ_C_Ant_B_RE_MD) RQ_C_Ant_B_MD_AA_C3 =1.

IF (RQ_C_Ant_B_MD>RQ_C_Ant_B_RE_MD) RQ_C_Ant_B_MD_AA_C3 =3.

IF (RQ_C_Ant_B_MD<RQ_C_Ant_B_RE_MD) RQ_C_Ant_B_MD_AA_C3 =2.

VARIABLE LABELS RQ_C_Ant_B_MD_AA_C3 'Absoluut agreement voor "Antwoord op vragen" test-thentest - MD, C3' .

VALUE LABELS RQ_C_Ant_B_MD_AA_C3

1 'test-thentest gelijk'

2 'test positiever dan hertest'

3 'test negatiever dan hertest' .

FREQUENCIES RQ_C_Ant_B_MD_AA_C3.

NUMERIC RQ_C_Adv_B_MD_AA_C3 (F2).

COMPUTE RQ_C_Adv_B_MD_AA_C3 = $SYSMIS.

IF (RQ_C_Adv_B_MD=RQ_C_Adv_B_RE_MD) RQ_C_Adv_B_MD_AA_C3 =1.

IF (RQ_C_Adv_B_MD>RQ_C_Adv_B_RE_MD) RQ_C_Adv_B_MD_AA_C3 =3.

IF (RQ_C_Adv_B_MD<RQ_C_Adv_B_RE_MD) RQ_C_Adv_B_MD_AA_C3 =2.

VARIABLE LABELS RQ_C_Adv_B_MD_AA_C3 'Absoluut agreement voor "Krijgen dezelfde adviezen" test-thentest - MD, C3' .

VALUE LABELS RQ_C_Adv_B_MD_AA_C3

1 'test-thentest gelijk'

2 'test positiever dan hertest'

3 'test negatiever dan hertest' .

FREQUENCIES RQ_C_Adv_B_MD_AA_C3.

NUMERIC RQ_C_Uit_B_MD_AA_C3 (F2).

COMPUTE RQ_C_Uit_B_MD_AA_C3 = $SYSMIS.

IF (RQ_C_Uit_B_MD=RQ_C_Uit_B_RE_MD) RQ_C_Uit_B_MD_AA_C3 =1.

IF (RQ_C_Uit_B_MD>RQ_C_Uit_B_RE_MD) RQ_C_Uit_B_MD_AA_C3 =3.

IF (RQ_C_Uit_B_MD<RQ_C_Uit_B_RE_MD) RQ_C_Uit_B_MD_AA_C3 =2.

VARIABLE LABELS RQ_C_Uit_B_MD_AA_C3 'Absoluut agreement voor "Begrijpen uitleg" test-thentest - MD, C3' .

VALUE LABELS RQ_C_Uit_B_MD_AA_C3

1 'test-thentest gelijk'

2 'test positiever dan hertest'

3 'test negatiever dan hertest' .

FREQUENCIES RQ_C_Uit_B_MD_AA_C3.

NUMERIC RQ_C_Inf_B_MD_AA_C3 (F2).

COMPUTE RQ_C_Inf_B_MD_AA_C3 = $SYSMIS.

IF (RQ_C_Inf_B_MD=RQ_C_Inf_B_RE_MD) RQ_C_Inf_B_MD_AA_C3 =1.

IF (RQ_C_Inf_B_MD>RQ_C_Inf_B_RE_MD) RQ_C_Inf_B_MD_AA_C3 =3.

IF (RQ_C_Inf_B_MD<RQ_C_Inf_B_RE_MD) RQ_C_Inf_B_MD_AA_C3 =2.

VARIABLE LABELS RQ_C_Inf_B_MD_AA_C3 'Absoluut agreement voor "Informatie tijdens behandeling" test-thentest - MD, C3' .

VALUE LABELS RQ_C_Inf_B_MD_AA_C3

1 'test-thentest gelijk'

2 'test positiever dan hertest'

3 'test negatiever dan hertest' .

FREQUENCIES RQ_C_Inf_B_MD_AA_C3.

*Tijd tot hulp.

NUMERIC RQ_T_Ghd_B_MD_AA_C3 (F2).

COMPUTE RQ_T_Ghd_B_MD_AA_C3 = $SYSMIS.

IF (RQ_T_Ghd_B_MD=RQ_T_Ghd_B_RE_MD) RQ_T_Ghd_B_MD_AA_C3 =1.

IF (RQ_T_Ghd_B_MD>RQ_T_Ghd_B_RE_MD) RQ_T_Ghd_B_MD_AA_C3 =3.

IF (RQ_T_Ghd_B_MD<RQ_T_Ghd_B_RE_MD) RQ_T_Ghd_B_MD_AA_C3 =2.

VARIABLE LABELS RQ_T_Ghd_B_MD_AA_C3 'Absoluut agreement voor "Hulp als dringend" test-thentest - MD, C3' .

VALUE LABELS RQ_T_Ghd_B_MD_AA_C3

1 'test-thentest gelijk'

2 'test positiever dan hertest'

3 'test negatiever dan hertest' .

FREQUENCIES RQ_T_Ghd_B_MD_AA_C3.

NUMERIC RQ_T_Gnd_B_MD_AA_C3 (F2).

COMPUTE RQ_T_Gnd_B_MD_AA_C3 = $SYSMIS.

IF (RQ_T_Gnd_B_MD=RQ_T_Gnd_B_RE_MD) RQ_T_Gnd_B_MD_AA_C3 =1.

IF (RQ_T_Gnd_B_MD>RQ_T_Gnd_B_RE_MD) RQ_T_Gnd_B_MD_AA_C3 =3.

IF (RQ_T_Gnd_B_MD<RQ_T_Gnd_B_RE_MD) RQ_T_Gnd_B_MD_AA_C3 =2.

VARIABLE LABELS RQ_T_Gnd_B_MD_AA_C3 'Absoluut agreement voor "Hulp als niet dringend" test-thentest - MD, C3' .

VALUE LABELS RQ_T_Gnd_B_MD_AA_C3

1 'test-thentest gelijk'

2 'test positiever dan hertest'

3 'test negatiever dan hertest' .

FREQUENCIES RQ_T_Gnd_B_MD_AA_C3.

NUMERIC RQ_T_Tyd_B_MD_AA_C3 (F2).

COMPUTE RQ_T_Tyd_B_MD_AA_C3 = $SYSMIS.

IF (RQ_T_TYD_B_MD=RQ_T_TYD_B_RE_MD) RQ_T_Tyd_B_MD_AA_C3 =1.

IF (RQ_T_TYD_B_MD>RQ_T_TYD_B_RE_MD) RQ_T_Tyd_B_MD_AA_C3 =3.

IF (RQ_T_TYD_B_MD<RQ_T_TYD_B_RE_MD) RQ_T_Tyd_B_MD_AA_C3 =2.

VARIABLE LABELS RQ_T_Tyd_B_MD_AA_C3 'Absoluut agreement voor "Tijd als nodig" test-thentest - MD, C3' .

VALUE LABELS RQ_T_Tyd_B_MD_AA_C3

1 'test-thentest gelijk'

2 'test positiever dan hertest'

3 'test negatiever dan hertest' .

FREQUENCIES RQ_T_Tyd_B_MD_AA_C3.

NUMERIC RQ_T_Anw_B_MD_AA_C3 (F2).

COMPUTE RQ_T_Anw_B_MD_AA_C3 = $SYSMIS.

IF (RQ_T_ANW_B_MD=RQ_T_ANW_B_RE_MD) RQ_T_Anw_B_MD_AA_C3 =1.

IF (RQ_T_ANW_B_MD>RQ_T_ANW_B_RE_MD) RQ_T_Anw_B_MD_AA_C3 =3.

IF (RQ_T_ANW_B_MD<RQ_T_ANW_B_RE_MD) RQ_T_Anw_B_MD_AA_C3 =2.

VARIABLE LABELS RQ_T_Anw_B_MD_AA_C3 'Absoluut agreement voor "bij afspraak snel aan de beurt" test-thentest - MD, C3' .

VALUE LABELS RQ_T_Anw_B_MD_AA_C3

1 'test-thentest gelijk'

2 'test positiever dan hertest'

3 'test negatiever dan hertest' .

FREQUENCIES RQ_T_Anw_B_MD_AA_C3.

NUMERIC RQ_T_BER_B_MD_AA_C3 (F2).

COMPUTE RQ_T_BER_B_MD_AA_C3 = $SYSMIS.

IF (RQ_T_BER_B_MD=RQ_T_BER_B_RE_MD) RQ_T_BER_B_MD_AA_C3 =1.

IF (RQ_T_BER_B_MD>RQ_T_BER_B_RE_MD) RQ_T_BER_B_MD_AA_C3 =3.

IF (RQ_T_BER_B_MD<RQ_T_BER_B_RE_MD) RQ_T_BER_B_MD_AA_C3 =2.

VARIABLE LABELS RQ_T_BER_B_MD_AA_C3 'Absoluut agreement voor "Bereikbaarheid locatie" test-thentest - MD, C3' .

VALUE LABELS RQ_T_BER_B_MD_AA_C3

1 'test-thentest gelijk'

2 'test positiever dan hertest'

3 'test negatiever dan hertest' .

FREQUENCIES RQ_T_BER_B_MD_AA_C3.

NUMERIC RQ_T_TEL_B_MD_AA_C3 (F2).

COMPUTE RQ_T_TEL_B_MD_AA_C3 = $SYSMIS.

IF (RQ_T_TEL_B_MD=RQ_T_TEL_B_RE_MD) RQ_T_TEL_B_MD_AA_C3 =1.

IF (RQ_T_TEL_B_MD>RQ_T_TEL_B_RE_MD) RQ_T_TEL_B_MD_AA_C3 =3.

IF (RQ_T_TEL_B_MD<RQ_T_TEL_B_RE_MD) RQ_T_TEL_B_MD_AA_C3 =2.

VARIABLE LABELS RQ_T_TEL_B_MD_AA_C3 'Absoluut agreement voor "Telefonische bereikbaarheid" test-thentest - MD, C3' .

VALUE LABELS RQ_T_TEL_B_MD_AA_C3

1 'test-thentest gelijk'

2 'test positiever dan hertest'

3 'test negatiever dan hertest' .

FREQUENCIES RQ_T_TEL_B_MD_AA_C3.

*Sociale ondersteuning.

NUMERIC RQ_S_Fam_B_MD_AA_C3 (F2).

COMPUTE RQ_S_Fam_B_MD_AA_C3 = $SYSMIS.

IF (RQ_S_Fam_B_MD=RQ_S_Fam_B_RE_MD) RQ_S_Fam_B_MD_AA_C3 =1.

IF (RQ_S_Fam_B_MD>RQ_S_Fam_B_RE_MD) RQ_S_Fam_B_MD_AA_C3 =3.

IF (RQ_S_Fam_B_MD<RQ_S_Fam_B_RE_MD) RQ_S_Fam_B_MD_AA_C3 =2.

VARIABLE LABELS RQ_S_Fam_B_MD_AA_C3 'Absoluut agreement voor "Betrekken familie" test-thentest - MD, C3' .

VALUE LABELS RQ_S_Fam_B_MD_AA_C3

1 'test-thentest gelijk'

2 'test positiever dan hertest'

3 'test negatiever dan hertest' .

FREQUENCIES RQ_S_Fam_B_MD_AA_C3.

NUMERIC RQ_S_Rhm_B_MD_AA_C3 (F2).

COMPUTE RQ_S_Rhm_B_MD_AA_C3 = $SYSMIS.

IF (RQ_S_Rhm_B_MD=RQ_S_Rhm_B_RE_MD) RQ_S_Rhm_B_MD_AA_C3 =1.

IF (RQ_S_Rhm_B_MD>RQ_S_Rhm_B_RE_MD) RQ_S_Rhm_B_MD_AA_C3 =3.

IF (RQ_S_Rhm_B_MD<RQ_S_Rhm_B_RE_MD) RQ_S_Rhm_B_MD_AA_C3 =2.

VARIABLE LABELS RQ_S_Rhm_B_MD_AA_C3 'Absoluut agreement voor "Rekening houden met gezin" test-thentest - MD, C3' .

VALUE LABELS RQ_S_Rhm_B_MD_AA_C3

1 'test-thentest gelijk'

2 'test positiever dan hertest'

3 'test negatiever dan hertest' .

FREQUENCIES RQ_S_Rhm_B_MD_AA_C3.

NUMERIC RQ_S_Ste_B_MD_AA_C3 (F2).

COMPUTE RQ_S_Ste_B_MD_AA_C3 = $SYSMIS.

IF (RQ_S_Ste_B_MD=RQ_S_Ste_B_RE_MD) RQ_S_Ste_B_MD_AA_C3 =1.

IF (RQ_S_Ste_B_MD>RQ_S_Ste_B_RE_MD) RQ_S_Ste_B_MD_AA_C3 =3.

IF (RQ_S_Ste_B_MD<RQ_S_Ste_B_RE_MD) RQ_S_Ste_B_MD_AA_C3 =2.

VARIABLE LABELS RQ_S_Ste_B_MD_AA_C3 'Absoluut agreement voor "Gesteund voelen" test-thentest - MD, C3' .

VALUE LABELS RQ_S_Ste_B_MD_AA_C3

1 'test-thentest gelijk'

2 'test positiever dan hertest'

3 'test negatiever dan hertest' .

FREQUENCIES RQ_S_Ste_B_MD_AA_C3.

*Faciliteiten.

NUMERIC RQ_F_Cmf_B_MD_AA_C3 (F2).

COMPUTE RQ_F_Cmf_B_MD_AA_C3 = $SYSMIS.

IF (RQ_F_Cmf_B_MD=RQ_F_Cmf_B_RE_MD) RQ_F_Cmf_B_MD_AA_C3 =1.

IF (RQ_F_Cmf_B_MD>RQ_F_Cmf_B_RE_MD) RQ_F_Cmf_B_MD_AA_C3 =3.

IF (RQ_F_Cmf_B_MD<RQ_F_Cmf_B_RE_MD) RQ_F_Cmf_B_MD_AA_C3 =2.

VARIABLE LABELS RQ_F_Cmf_B_MD_AA_C3 'Absoluut agreement voor "Comfort" test-thentest - MD, C3' .

VALUE LABELS RQ_F_Cmf_B_MD_AA_C3

1 'test-thentest gelijk'

2 'test positiever dan hertest'

3 'test negatiever dan hertest' .

FREQUENCIES RQ_F_Cmf_B_MD_AA_C3.

NUMERIC RQ_F_Hyg_B_MD_AA_C3 (F2).

COMPUTE RQ_F_Hyg_B_MD_AA_C3 = $SYSMIS.

IF (RQ_F_Hyg_B_MD=RQ_F_Hyg_B_RE_MD) RQ_F_Hyg_B_MD_AA_C3 =1.

IF (RQ_F_Hyg_B_MD>RQ_F_Hyg_B_RE_MD) RQ_F_Hyg_B_MD_AA_C3 =3.

IF (RQ_F_Hyg_B_MD<RQ_F_Hyg_B_RE_MD) RQ_F_Hyg_B_MD_AA_C3 =2.

VARIABLE LABELS RQ_F_Hyg_B_MD_AA_C3 'Absoluut agreement voor "Hygiene" test-thentest - MD, C3' .

VALUE LABELS RQ_F_Hyg_B_MD_AA_C3

1 'test-thentest gelijk'

2 'test positiever dan hertest'

3 'test negatiever dan hertest' .

FREQUENCIES RQ_F_Hyg_B_MD_AA_C3.

NUMERIC RQ_F_Toe_B_MD_AA_C3 (F2).

COMPUTE RQ_F_Toe_B_MD_AA_C3 = $SYSMIS.

IF (RQ_F_Toe_B_MD=RQ_F_Toe_B_RE_MD) RQ_F_Toe_B_MD_AA_C3 =1.

IF (RQ_F_Toe_B_MD>RQ_F_Toe_B_RE_MD) RQ_F_Toe_B_MD_AA_C3 =3.

IF (RQ_F_Toe_B_MD<RQ_F_Toe_B_RE_MD) RQ_F_Toe_B_MD_AA_C3 =2.

VARIABLE LABELS RQ_F_Toe_B_MD_AA_C3 'Absoluut agreement voor "Toegankelijkheid ruimtes" test-thentest - MD, C3' .

VALUE LABELS RQ_F_Toe_B_MD_AA_C3

1 'test-thentest gelijk'

2 'test positiever dan hertest'

3 'test negatiever dan hertest' .

FREQUENCIES RQ_F_Toe_B_MD_AA_C3.

*Keuze en continuiteit.

NUMERIC RQ_K_Wis_B_MD_AA_C3 (F2).

COMPUTE RQ_K_Wis_B_MD_AA_C3 = $SYSMIS.

IF (RQ_K_Wis_B_MD=RQ_K_Wis_B_RE_MD) RQ_K_Wis_B_MD_AA_C3 =1.

IF (RQ_K_Wis_B_MD>RQ_K_Wis_B_RE_MD) RQ_K_Wis_B_MD_AA_C3 =3.

IF (RQ_K_Wis_B_MD<RQ_K_Wis_B_RE_MD) RQ_K_Wis_B_MD_AA_C3 =2.

VARIABLE LABELS RQ_K_Wis_B_MD_AA_C3 'Absoluut agreement voor "Wisselen zorgverlener" test-thentest - MD, C3' .

VALUE LABELS RQ_K_Wis_B_MD_AA_C3

1 'test-thentest gelijk'

2 'test positiever dan hertest'

3 'test negatiever dan hertest' .

FREQUENCIES RQ_K_Wis_B_MD_AA_C3.

NUMERIC RQ_K_Vwz_B_MD_AA_C3 (F2).

COMPUTE RQ_K_Vwz_B_MD_AA_C3 = $SYSMIS.

IF (RQ_K_Vwz_B_MD=RQ_K_Vwz_B_RE_MD) RQ_K_Vwz_B_MD_AA_C3 =1.

IF (RQ_K_Vwz_B_MD>RQ_K_Vwz_B_RE_MD) RQ_K_Vwz_B_MD_AA_C3 =3.

IF (RQ_K_Vwz_B_MD<RQ_K_Vwz_B_RE_MD) RQ_K_Vwz_B_MD_AA_C3 =2.

VARIABLE LABELS RQ_K_Vwz_B_MD_AA_C3 'Absoluut agreement voor "Verwijzen naar ziekenhuis" test-thentest - MD, C3' .

VALUE LABELS RQ_K_Vwz_B_MD_AA_C3

1 'test-thentest gelijk'

2 'test positiever dan hertest'

3 'test negatiever dan hertest' .

FREQUENCIES RQ_K_Vwz_B_MD_AA_C3.

NUMERIC RQ_K_Soo_B_MD_AA_C3 (F2).

COMPUTE RQ_K_Soo_B_MD_AA_C3 = $SYSMIS.

IF (RQ_K_Soo_B_MD=RQ_K_Soo_B_RE_MD) RQ_K_Soo_B_MD_AA_C3 =1.

IF (RQ_K_Soo_B_MD>RQ_K_Soo_B_RE_MD) RQ_K_Soo_B_MD_AA_C3 =3.

IF (RQ_K_Soo_B_MD<RQ_K_Soo_B_RE_MD) RQ_K_Soo_B_MD_AA_C3 =2.

VARIABLE LABELS RQ_K_Soo_B_MD_AA_C3 'Absoluut agreement voor "Keuze soort zorgverlener" test-thentest - MD, C3' .

VALUE LABELS RQ_K_Soo_B_MD_AA_C3

1 'test-thentest gelijk'

2 'test positiever dan hertest'

3 'test negatiever dan hertest' .

FREQUENCIES RQ_K_Soo_B_MD_AA_C3.

NUMERIC RQ_K_Lei_B_MD_AA_C3 (F2).

COMPUTE RQ_K_Lei_B_MD_AA_C3 = $SYSMIS.

IF (RQ_K_Lei_B_MD=RQ_K_Lei_B_RE_MD) RQ_K_Lei_B_MD_AA_C3 =1.

IF (RQ_K_Lei_B_MD>RQ_K_Lei_B_RE_MD) RQ_K_Lei_B_MD_AA_C3 =3.

IF (RQ_K_Lei_B_MD<RQ_K_Lei_B_RE_MD) RQ_K_Lei_B_MD_AA_C3 =2.

VARIABLE LABELS RQ_K_Lei_B_MD_AA_C3 'Absoluut agreement voor "Leiding zorg" test-thentest - MD, C3' .

VALUE LABELS RQ_K_Lei_B_MD_AA_C3

1 'test-thentest gelijk'

2 'test positiever dan hertest'

3 'test negatiever dan hertest' .

FREQUENCIES RQ_K_Lei_B_MD_AA_C3.

### Mean score

*Respect.

NUMERIC RQ_R_Pri_B_Mean_AA_C3 (F2).

COMPUTE RQ_R_Pri_B_Mean_AA_C3 = $SYSMIS.

IF (RQ_R_Pri_B_M=RQ_R_Pri_B_RE_M) RQ_R_Pri_B_Mean_AA_C3 =1.

IF (RQ_R_Pri_B_M>RQ_R_Pri_B_RE_M) RQ_R_Pri_B_Mean_AA_C3 =2.

IF (RQ_R_Pri_B_M<RQ_R_Pri_B_RE_M) RQ_R_Pri_B_Mean_AA_C3 =3.

VARIABLE LABELS RQ_R_Pri_B_Mean_AA_C3 'Absoluut agreement voor "Rekening houden privacy" test-thentest - MEAN, C3' .

VALUE LABELS RQ_R_Pri_B_Mean_AA_C3

1 'test-thentest gelijk'

2 'test positiever dan hertest'

3 'test negatiever dan hertest' .

FREQUENCIES RQ_R_Pri_B_Mean_AA_C3.

NUMERIC RQ_R_RES_B_Mean_AA_C3 (F2).

COMPUTE RQ_R_RES_B_Mean_AA_C3 = $SYSMIS.

IF (RQ_R_RES_B_M=RQ_R_RES_B_RE_M) RQ_R_RES_B_Mean_AA_C3 =1.

IF (RQ_R_RES_B_M>RQ_R_RES_B_RE_M) RQ_R_RES_B_Mean_AA_C3 =2.

IF (RQ_R_RES_B_M<RQ_R_RES_B_RE_M) RQ_R_RES_B_Mean_AA_C3 =3.

VARIABLE LABELS RQ_R_RES_B_Mean_AA_C3 'Absoluut agreement voor "Behandeld met respect" test-thentest - MEAN, C3' .

VALUE LABELS RQ_R_RES_B_Mean_AA_C3

1 'test-thentest gelijk'

2 'test positiever dan hertest'

3 'test negatiever dan hertest' .

FREQUENCIES RQ_R_RES_B_Mean_AA_C3.

NUMERIC RQ_R_Per_B_Mean_AA_C3 (F2).

COMPUTE RQ_R_Per_B_Mean_AA_C3 = $SYSMIS.

IF (RQ_R_Per_B_M=RQ_R_Per_B_RE_M) RQ_R_Per_B_Mean_AA_C3 =1.

IF (RQ_R_Per_B_M>RQ_R_Per_B_RE_M) RQ_R_Per_B_Mean_AA_C3 =2.

IF (RQ_R_Per_B_M<RQ_R_Per_B_RE_M) RQ_R_Per_B_Mean_AA_C3 =3.

VARIABLE LABELS RQ_R_Per_B_Mean_AA_C3 'Absoluut agreement voor "Persoonlijke aandacht" test-thentest - MEAN, C3' .

VALUE LABELS RQ_R_Per_B_Mean_AA_C3

1 'test-thentest gelijk'

2 'test positiever dan hertest'

3 'test negatiever dan hertest' .

FREQUENCIES RQ_R_Per_B_Mean_AA_C3.

NUMERIC RQ_R_Vri_B_Mean_AA_C3 (F2).

COMPUTE RQ_R_Vri_B_Mean_AA_C3 = $SYSMIS.

IF (RQ_R_Vri_B_M=RQ_R_Vri_B_RE_M) RQ_R_Vri_B_Mean_AA_C3 =1.

IF (RQ_R_Vri_B_M>RQ_R_Vri_B_RE_M) RQ_R_Vri_B_Mean_AA_C3 =2.

IF (RQ_R_Vri_B_M<RQ_R_Vri_B_RE_M) RQ_R_Vri_B_Mean_AA_C3 =3.

VARIABLE LABELS RQ_R_Vri_B_Mean_AA_C3 'Absoluut agreement voor "Vriendelijk behandeld" test-thentest - MEAN, C3' .

VALUE LABELS RQ_R_Vri_B_Mean_AA_C3

1 'test-thentest gelijk'

2 'test positiever dan hertest'

3 'test negatiever dan hertest' .

FREQUENCIES RQ_R_Vri_B_Mean_AA_C3.

NUMERIC RQ_R_Wen_B_Mean_AA_C3 (F2).

COMPUTE RQ_R_Wen_B_Mean_AA_C3 = $SYSMIS.

IF (RQ_R_Wen_B_M=RQ_R_Wen_B_RE_M) RQ_R_Wen_B_Mean_AA_C3 =1.

IF (RQ_R_Wen_B_M>RQ_R_Wen_B_RE_M) RQ_R_Wen_B_Mean_AA_C3 =2.

IF (RQ_R_Wen_B_M<RQ_R_Wen_B_RE_M) RQ_R_Wen_B_Mean_AA_C3 =3.

VARIABLE LABELS RQ_R_Wen_B_Mean_AA_C3 'Absoluut agreement voor "Wensen en behoeften" test-thentest - MEAN, C3' .

VALUE LABELS RQ_R_Wen_B_Mean_AA_C3

1 'test-thentest gelijk'

2 'test positiever dan hertest'

3 'test negatiever dan hertest' .

FREQUENCIES RQ_R_Wen_B_Mean_AA_C3.

NUMERIC RQ_R_Ver_B_Mean_AA_C3 (F2).

COMPUTE RQ_R_Ver_B_Mean_AA_C3 = $SYSMIS.

IF (RQ_R_Ver_B_M=RQ_R_Ver_B_RE_M) RQ_R_Ver_B_Mean_AA_C3 =1.

IF (RQ_R_Ver_B_M>RQ_R_Ver_B_RE_M) RQ_R_Ver_B_Mean_AA_C3 =2.

IF (RQ_R_Ver_B_M<RQ_R_Ver_B_RE_M) RQ_R_Ver_B_Mean_AA_C3 =3.

VARIABLE LABELS RQ_R_Ver_B_Mean_AA_C3 'Absoluut agreement voor "Vertrouwen" test-thentest - MEAN, C3' .

VALUE LABELS RQ_R_Ver_B_Mean_AA_C3

1 'test-thentest gelijk'

2 'test positiever dan hertest'

3 'test negatiever dan hertest' .

FREQUENCIES RQ_R_Ver_B_Mean_AA_C3.

*autonomie.

NUMERIC RQ_A_Wei_B_Mean_AA_C3 (F2).

COMPUTE RQ_A_Wei_B_Mean_AA_C3 = $SYSMIS.

IF (RQ_A_Wei_B_M=RQ_A_Wei_B_RE_M) RQ_A_Wei_B_Mean_AA_C3 =1.

IF (RQ_A_Wei_B_M>RQ_A_Wei_B_RE_M) RQ_A_Wei_B_Mean_AA_C3 =2.

IF (RQ_A_Wei_B_M<RQ_A_Wei_B_RE_M) RQ_A_Wei_B_Mean_AA_C3 =3.

VARIABLE LABELS RQ_A_Wei_B_Mean_AA_C3 'Absoluut agreement voor "weigeren behandeling" test-thentest - MEAN, C3' .

VALUE LABELS RQ_A_Wei_B_Mean_AA_C3

1 'test-thentest gelijk'

2 'test positiever dan hertest'

3 'test negatiever dan hertest' .

FREQUENCIES RQ_A_Wei_B_Mean_AA_C3.

NUMERIC RQ_A_Mee_B_Mean_AA_C3 (F2).

COMPUTE RQ_A_Mee_B_Mean_AA_C3 = $SYSMIS.

IF (RQ_A_Mee_B_M=RQ_A_Mee_B_RE_M) RQ_A_Mee_B_Mean_AA_C3 =1.

IF (RQ_A_Mee_B_M>RQ_A_Mee_B_RE_M) RQ_A_Mee_B_Mean_AA_C3 =2.

IF (RQ_A_Mee_B_M<RQ_A_Mee_B_RE_M) RQ_A_Mee_B_Mean_AA_C3 =3.

VARIABLE LABELS RQ_A_Mee_B_Mean_AA_C3 'Absoluut agreement voor "Meebeslissen behandeling" test-thentest - MEAN, C3' .

VALUE LABELS RQ_A_Mee_B_Mean_AA_C3

1 'test-thentest gelijk'

2 'test positiever dan hertest'

3 'test negatiever dan hertest' .

FREQUENCIES RQ_A_Mee_B_Mean_AA_C3.

NUMERIC RQ_A_SvD_B_Mean_AA_C3 (F2).

COMPUTE RQ_A_SvD_B_Mean_AA_C3 = $SYSMIS.

IF (RQ_A_SvD_M_Cat3=RQ_A_SvD_Re_M_Cat3) RQ_A_SvD_B_Mean_AA_C3 =1.

IF (RQ_A_SvD_M_Cat3>RQ_A_SvD_Re_M_Cat3) RQ_A_SvD_B_Mean_AA_C3 =2.

IF (RQ_A_SvD_M_Cat3<RQ_A_SvD_Re_M_Cat3) RQ_A_SvD_B_Mean_AA_C3 =3.

VARIABLE LABELS RQ_A_SvD_B_Mean_AA_C3 'Absoluut agreement voor "Syndroom van down" test-thentest - MEAN, C3' .

VALUE LABELS RQ_A_SvD_B_Mean_AA_C3

1 'test-thentest gelijk'

2 'test positiever dan hertest'

3 'test negatiever dan hertest' .

FREQUENCIES RQ_A_SvD_B_Mean_AA_C3.

NUMERIC RQ_A_Gbp_B_Mean_AA_C3 (F2).

COMPUTE RQ_A_Gbp_B_Mean_AA_C3 = $SYSMIS.

IF (RQ_A_Gbp_B_M_Cat5=RQ_A_Gbp_Re_M_Cat5) RQ_A_Gbp_B_Mean_AA_C3 =1.

IF (RQ_A_Gbp_B_M_Cat5>RQ_A_Gbp_Re_M_Cat5) RQ_A_Gbp_B_Mean_AA_C3 =2.

IF (RQ_A_Gbp_B_M_Cat5<RQ_A_Gbp_Re_M_Cat5) RQ_A_Gbp_B_Mean_AA_C3 =3.

VARIABLE LABELS RQ_A_Gbp_B_Mean_AA_C3 'Absoluut agreement voor "Geboorteplan" test-thentest - MEAN, C3' .

VALUE LABELS RQ_A_Gbp_B_Mean_AA_C3

1 'test-thentest gelijk'

2 'test positiever dan hertest'

3 'test negatiever dan hertest' .

FREQUENCIES RQ_A_Gbp_B_Mean_AA_C3.

*Privacy.

NUMERIC RQ_P_Med_B_Mean_AA_C3 (F2).

COMPUTE RQ_P_Med_B_Mean_AA_C3 = $SYSMIS.

IF (RQ_P_Med_B_M=RQ_P_Med_B_RE_M) RQ_P_Med_B_Mean_AA_C3 =1.

IF (RQ_P_Med_B_M>RQ_P_Med_B_RE_M) RQ_P_Med_B_Mean_AA_C3 =2.

IF (RQ_P_Med_B_M<RQ_P_Med_B_RE_M) RQ_P_Med_B_Mean_AA_C3 =3.

VARIABLE LABELS RQ_P_Med_B_Mean_AA_C3 'Absoluut agreement voor "Medisch dossier" test-thentest - MEAN, C3' .

VALUE LABELS RQ_P_Med_B_Mean_AA_C3

1 'test-thentest gelijk'

2 'test positiever dan hertest'

3 'test negatiever dan hertest' .

FREQUENCIES RQ_P_Med_B_Mean_AA_C3.

NUMERIC RQ_P_MLN_B_Mean_AA_C3 (F2).

COMPUTE RQ_P_MLN_B_Mean_AA_C3 = $SYSMIS.

IF (RQ_P_Mln_B_M=RQ_P_Mln_B_RE_M) RQ_P_MLN_B_Mean_AA_C3 =1.

IF (RQ_P_Mln_B_M>RQ_P_Mln_B_RE_M) RQ_P_MLN_B_Mean_AA_C3 =2.

IF (RQ_P_Mln_B_M<RQ_P_Mln_B_RE_M) RQ_P_MLN_B_Mean_AA_C3 =3.

VARIABLE LABELS RQ_P_MLN_B_Mean_AA_C3 'Absoluut agreement voor "Meeluisteren" test-thentest - MEAN, C3' .

VALUE LABELS RQ_P_MLN_B_Mean_AA_C3

1 'test-thentest gelijk'

2 'test positiever dan hertest'

3 'test negatiever dan hertest' .

FREQUENCIES RQ_P_MLN_B_Mean_AA_C3.

*Communicatie.

NUMERIC RQ_C_Ant_B_Mean_AA_C3 (F2).

COMPUTE RQ_C_Ant_B_Mean_AA_C3 = $SYSMIS.

IF (RQ_C_Ant_B_M=RQ_C_Ant_B_RE_M) RQ_C_Ant_B_Mean_AA_C3 =1.

IF (RQ_C_Ant_B_M>RQ_C_Ant_B_RE_M) RQ_C_Ant_B_Mean_AA_C3 =2.

IF (RQ_C_Ant_B_M<RQ_C_Ant_B_RE_M) RQ_C_Ant_B_Mean_AA_C3 =3.

VARIABLE LABELS RQ_C_Ant_B_Mean_AA_C3 'Absoluut agreement voor "Antwoord op vragen" test-thentest - MEAN, C3' .

VALUE LABELS RQ_C_Ant_B_Mean_AA_C3

1 'test-thentest gelijk'

2 'test positiever dan hertest'

3 'test negatiever dan hertest' .

FREQUENCIES RQ_C_Ant_B_Mean_AA_C3.

NUMERIC RQ_C_Adv_B_Mean_AA_C3 (F2).

COMPUTE RQ_C_Adv_B_Mean_AA_C3 = $SYSMIS.

IF (RQ_C_Adv_B_M=RQ_C_Adv_B_RE_M) RQ_C_Adv_B_Mean_AA_C3 =1.

IF (RQ_C_Adv_B_M>RQ_C_Adv_B_RE_M) RQ_C_Adv_B_Mean_AA_C3 =2.

IF (RQ_C_Adv_B_M<RQ_C_Adv_B_RE_M) RQ_C_Adv_B_Mean_AA_C3 =3.

VARIABLE LABELS RQ_C_Adv_B_Mean_AA_C3 'Absoluut agreement voor "Krijgen dezelfde adviezen" test-thentest - MEAN, C3' .

VALUE LABELS RQ_C_Adv_B_Mean_AA_C3

1 'test-thentest gelijk'

2 'test positiever dan hertest'

3 'test negatiever dan hertest' .

FREQUENCIES RQ_C_Adv_B_Mean_AA_C3.

NUMERIC RQ_C_Uit_B_Mean_AA_C3 (F2).

COMPUTE RQ_C_Uit_B_Mean_AA_C3 = $SYSMIS.

IF (RQ_C_Uit_B_M=RQ_C_Uit_B_RE_M) RQ_C_Uit_B_Mean_AA_C3 =1.

IF (RQ_C_Uit_B_M>RQ_C_Uit_B_RE_M) RQ_C_Uit_B_Mean_AA_C3 =2.

IF (RQ_C_Uit_B_M<RQ_C_Uit_B_RE_M) RQ_C_Uit_B_Mean_AA_C3 =3.

VARIABLE LABELS RQ_C_Uit_B_Mean_AA_C3 'Absoluut agreement voor "Begrijpen uitleg" test-thentest - MEAN, C3' .

VALUE LABELS RQ_C_Uit_B_Mean_AA_C3

1 'test-thentest gelijk'

2 'test positiever dan hertest'

3 'test negatiever dan hertest' .

FREQUENCIES RQ_C_Uit_B_Mean_AA_C3.

NUMERIC RQ_C_Inf_B_Mean_AA_C3 (F2).

COMPUTE RQ_C_Inf_B_Mean_AA_C3 = $SYSMIS.

IF (RQ_C_Inf_B_M=RQ_C_Inf_B_RE_M) RQ_C_Inf_B_Mean_AA_C3 =1.

IF (RQ_C_Inf_B_M>RQ_C_Inf_B_RE_M) RQ_C_Inf_B_Mean_AA_C3 =2.

IF (RQ_C_Inf_B_M<RQ_C_Inf_B_RE_M) RQ_C_Inf_B_Mean_AA_C3 =3.

VARIABLE LABELS RQ_C_Inf_B_Mean_AA_C3 'Absoluut agreement voor "Informatie tijdens behandeling" test-thentest - MEAN, C3' .

VALUE LABELS RQ_C_Inf_B_Mean_AA_C3

1 'test-thentest gelijk'

2 'test positiever dan hertest'

3 'test negatiever dan hertest' .

FREQUENCIES RQ_C_Inf_B_Mean_AA_C3.

*Tijd tot hulp.

NUMERIC RQ_T_Ghd_B_Mean_AA_C3 (F2).

COMPUTE RQ_T_Ghd_B_Mean_AA_C3 = $SYSMIS.

IF (RQ_T_Ghd_B_M_Cat5=RQ_T_Ghd_B_Re_M_Cat5) RQ_T_Ghd_B_Mean_AA_C3 =1.

IF (RQ_T_Ghd_B_M_Cat5>RQ_T_Ghd_B_Re_M_Cat5) RQ_T_Ghd_B_Mean_AA_C3 =2.

IF (RQ_T_Ghd_B_M_Cat5<RQ_T_Ghd_B_Re_M_Cat5) RQ_T_Ghd_B_Mean_AA_C3 =3.

VARIABLE LABELS RQ_T_Ghd_B_Mean_AA_C3 'Absoluut agreement voor "Hulp als dringend" test-thentest - MEAN, C3' .

VALUE LABELS RQ_T_Ghd_B_Mean_AA_C3

1 'test-thentest gelijk'

2 'test positiever dan hertest'

3 'test negatiever dan hertest' .

FREQUENCIES RQ_T_Ghd_B_Mean_AA_C3.

NUMERIC RQ_T_Gnd_B_Mean_AA_C3 (F2).

COMPUTE RQ_T_Gnd_B_Mean_AA_C3 = $SYSMIS.

IF (RQ_T_Gnd_B_M=RQ_T_Gnd_B_RE_M) RQ_T_Gnd_B_Mean_AA_C3 =1.

IF (RQ_T_Gnd_B_M>RQ_T_Gnd_B_RE_M) RQ_T_Gnd_B_Mean_AA_C3 =2.

IF (RQ_T_Gnd_B_M<RQ_T_Gnd_B_RE_M) RQ_T_Gnd_B_Mean_AA_C3 =3.

VARIABLE LABELS RQ_T_Gnd_B_Mean_AA_C3 'Absoluut agreement voor "Hulp als niet dringend" test-thentest - MEAN, C3' .

VALUE LABELS RQ_T_Gnd_B_Mean_AA_C3

1 'test-thentest gelijk'

2 'test positiever dan hertest'

3 'test negatiever dan hertest' .

FREQUENCIES RQ_T_Gnd_B_Mean_AA_C3.

NUMERIC RQ_T_Tyd_B_Mean_AA_C3 (F2).

COMPUTE RQ_T_Tyd_B_Mean_AA_C3 = $SYSMIS.

IF (RQ_T_TYD_B_M=RQ_T_TYD_B_RE_M) RQ_T_Tyd_B_Mean_AA_C3 =1.

IF (RQ_T_TYD_B_M>RQ_T_TYD_B_RE_M) RQ_T_Tyd_B_Mean_AA_C3 =2.

IF (RQ_T_TYD_B_M<RQ_T_TYD_B_RE_M) RQ_T_Tyd_B_Mean_AA_C3 =3.

VARIABLE LABELS RQ_T_Tyd_B_Mean_AA_C3 'Absoluut agreement voor "Tijd als nodig" test-thentest - MEAN, C3' .

VALUE LABELS RQ_T_Tyd_B_Mean_AA_C3

1 'test-thentest gelijk'

2 'test positiever dan hertest'

3 'test negatiever dan hertest' .

FREQUENCIES RQ_T_Tyd_B_Mean_AA_C3.

NUMERIC RQ_T_Anw_B_Mean_AA_C3 (F2).

COMPUTE RQ_T_Anw_B_Mean_AA_C3 = $SYSMIS.

IF (RQ_T_ANW_B_M=RQ_T_ANW_B_RE_M) RQ_T_Anw_B_Mean_AA_C3 =1.

IF (RQ_T_ANW_B_M>RQ_T_ANW_B_RE_M) RQ_T_Anw_B_Mean_AA_C3 =2.

IF (RQ_T_ANW_B_M<RQ_T_ANW_B_RE_M) RQ_T_Anw_B_Mean_AA_C3 =3.

VARIABLE LABELS RQ_T_Anw_B_Mean_AA_C3 'Absoluut agreement voor "bij afspraak snel aan de beurt" test-thentest - MEAN, C3' .

VALUE LABELS RQ_T_Anw_B_Mean_AA_C3

1 'test-thentest gelijk'

2 'test positiever dan hertest'

3 'test negatiever dan hertest' .

FREQUENCIES RQ_T_Anw_B_Mean_AA_C3.

NUMERIC RQ_T_BER_B_Mean_AA_C3 (F2).

COMPUTE RQ_T_BER_B_Mean_AA_C3 = $SYSMIS.

IF (RQ_T_BER_B_M=RQ_T_BER_B_RE_M) RQ_T_BER_B_Mean_AA_C3 =1.

IF (RQ_T_BER_B_M>RQ_T_BER_B_RE_M) RQ_T_BER_B_Mean_AA_C3 =2.

IF (RQ_T_BER_B_M<RQ_T_BER_B_RE_M) RQ_T_BER_B_Mean_AA_C3 =3.

VARIABLE LABELS RQ_T_BER_B_Mean_AA_C3 'Absoluut agreement voor "Bereikbaarheid locatie" test-thentest - MEAN, C3' .

VALUE LABELS RQ_T_BER_B_Mean_AA_C3

1 'test-thentest gelijk'

2 'test positiever dan hertest'

3 'test negatiever dan hertest' .

FREQUENCIES RQ_T_BER_B_Mean_AA_C3.

NUMERIC RQ_T_TEL_B_Mean_AA_C3 (F2).

COMPUTE RQ_T_TEL_B_Mean_AA_C3 = $SYSMIS.

IF (RQ_T_TEL_B_M=RQ_T_TEL_B_RE_M) RQ_T_TEL_B_Mean_AA_C3 =1.

IF (RQ_T_TEL_B_M>RQ_T_TEL_B_RE_M) RQ_T_TEL_B_Mean_AA_C3 =2.

IF (RQ_T_TEL_B_M<RQ_T_TEL_B_RE_M) RQ_T_TEL_B_Mean_AA_C3 =3.

VARIABLE LABELS RQ_T_TEL_B_Mean_AA_C3 'Absoluut agreement voor "Telefonische bereikbaarheid" test-thentest - MEAN, C3' .

VALUE LABELS RQ_T_TEL_B_Mean_AA_C3

1 'test-thentest gelijk'

2 'test positiever dan hertest'

3 'test negatiever dan hertest' .

FREQUENCIES RQ_T_TEL_B_Mean_AA_C3.

*Sociale ondersteuning.

NUMERIC RQ_S_Fam_B_Mean_AA_C3 (F2).

COMPUTE RQ_S_Fam_B_Mean_AA_C3 = $SYSMIS.

IF (RQ_S_Fam_B_M_Cat5=RQ_S_Fam_B_Re_M_cat5) RQ_S_Fam_B_Mean_AA_C3 =1.

IF (RQ_S_Fam_B_M_Cat5>RQ_S_Fam_B_Re_M_cat5) RQ_S_Fam_B_Mean_AA_C3 =2.

IF (RQ_S_Fam_B_M_Cat5<RQ_S_Fam_B_Re_M_cat5) RQ_S_Fam_B_Mean_AA_C3 =3.

VARIABLE LABELS RQ_S_Fam_B_Mean_AA_C3 'Absoluut agreement voor "Betrekken familie" test-thentest - MEAN, C3' .

VALUE LABELS RQ_S_Fam_B_Mean_AA_C3

1 'test-thentest gelijk'

2 'test positiever dan hertest'

3 'test negatiever dan hertest' .

FREQUENCIES RQ_S_Fam_B_Mean_AA_C3.

NUMERIC RQ_S_Rhm_B_Mean_AA_C3 (F2).

COMPUTE RQ_S_Rhm_B_Mean_AA_C3 = $SYSMIS.

IF (RQ_S_Rhm_B_M=RQ_S_Rhm_B_RE_M) RQ_S_Rhm_B_Mean_AA_C3 =1.

IF (RQ_S_Rhm_B_M>RQ_S_Rhm_B_RE_M) RQ_S_Rhm_B_Mean_AA_C3 =2.

IF (RQ_S_Rhm_B_M<RQ_S_Rhm_B_RE_M) RQ_S_Rhm_B_Mean_AA_C3 =3.

VARIABLE LABELS RQ_S_Rhm_B_Mean_AA_C3 'Absoluut agreement voor "Rekening houden met gezin" test-thentest - MEAN, C3' .

VALUE LABELS RQ_S_Rhm_B_Mean_AA_C3

1 'test-thentest gelijk'

2 'test positiever dan hertest'

3 'test negatiever dan hertest' .

FREQUENCIES RQ_S_Rhm_B_Mean_AA_C3.

NUMERIC RQ_S_Ste_B_Mean_AA_C3 (F2).

COMPUTE RQ_S_Ste_B_Mean_AA_C3 = $SYSMIS.

IF (RQ_S_Ste_B_M=RQ_S_Ste_B_RE_M) RQ_S_Ste_B_Mean_AA_C3 =1.

IF (RQ_S_Ste_B_M>RQ_S_Ste_B_RE_M) RQ_S_Ste_B_Mean_AA_C3 =2.

IF (RQ_S_Ste_B_M<RQ_S_Ste_B_RE_M) RQ_S_Ste_B_Mean_AA_C3 =3.

VARIABLE LABELS RQ_S_Ste_B_Mean_AA_C3 'Absoluut agreement voor "Gesteund voelen" test-thentest - MEAN, C3' .

VALUE LABELS RQ_S_Ste_B_Mean_AA_C3

1 'test-thentest gelijk'

2 'test positiever dan hertest'

3 'test negatiever dan hertest' .

FREQUENCIES RQ_S_Ste_B_Mean_AA_C3.

*Faciliteiten.

NUMERIC RQ_F_Cmf_B_Mean_AA_C3 (F2).

COMPUTE RQ_F_Cmf_B_Mean_AA_C3 = $SYSMIS.

IF (RQ_F_Cmf_B_M=RQ_F_Cmf_B_RE_M) RQ_F_Cmf_B_Mean_AA_C3 =1.

IF (RQ_F_Cmf_B_M>RQ_F_Cmf_B_RE_M) RQ_F_Cmf_B_Mean_AA_C3 =2.

IF (RQ_F_Cmf_B_M<RQ_F_Cmf_B_RE_M) RQ_F_Cmf_B_Mean_AA_C3 =3.

VARIABLE LABELS RQ_F_Cmf_B_Mean_AA_C3 'Absoluut agreement voor "Comfort" test-thentest - MEAN, C3' .

VALUE LABELS RQ_F_Cmf_B_Mean_AA_C3

1 'test-thentest gelijk'

2 'test positiever dan hertest'

3 'test negatiever dan hertest' .

FREQUENCIES RQ_F_Cmf_B_Mean_AA_C3.

NUMERIC RQ_F_Hyg_B_Mean_AA_C3 (F2).

COMPUTE RQ_F_Hyg_B_Mean_AA_C3 = $SYSMIS.

IF (RQ_F_Hyg_B_M=RQ_F_Hyg_B_RE_M) RQ_F_Hyg_B_Mean_AA_C3 =1.

IF (RQ_F_Hyg_B_M>RQ_F_Hyg_B_RE_M) RQ_F_Hyg_B_Mean_AA_C3 =2.

IF (RQ_F_Hyg_B_M<RQ_F_Hyg_B_RE_M) RQ_F_Hyg_B_Mean_AA_C3 =3.

VARIABLE LABELS RQ_F_Hyg_B_Mean_AA_C3 'Absoluut agreement voor "Hygiene" test-thentest - MEAN, C3' .

VALUE LABELS RQ_F_Hyg_B_Mean_AA_C3

1 'test-thentest gelijk'

2 'test positiever dan hertest'

3 'test negatiever dan hertest' .

FREQUENCIES RQ_F_Hyg_B_Mean_AA_C3.

NUMERIC RQ_F_Toe_B_Mean_AA_C3 (F2).

COMPUTE RQ_F_Toe_B_Mean_AA_C3 = $SYSMIS.

IF (RQ_F_Toe_B_M=RQ_F_Toe_B_RE_M) RQ_F_Toe_B_Mean_AA_C3 =1.

IF (RQ_F_Toe_B_M>RQ_F_Toe_B_RE_M) RQ_F_Toe_B_Mean_AA_C3 =2.

IF (RQ_F_Toe_B_M<RQ_F_Toe_B_RE_M) RQ_F_Toe_B_Mean_AA_C3 =3.

VARIABLE LABELS RQ_F_Toe_B_Mean_AA_C3 'Absoluut agreement voor "Toegankelijkheid ruimtes" test-thentest - MEAN, C3' .

VALUE LABELS RQ_F_Toe_B_Mean_AA_C3

1 'test-thentest gelijk'

2 'test positiever dan hertest'

3 'test negatiever dan hertest' .

FREQUENCIES RQ_F_Toe_B_Mean_AA_C3.

*Keuze en continuiteit.

NUMERIC RQ_K_Wis_B_Mean_AA_C3 (F2).

COMPUTE RQ_K_Wis_B_Mean_AA_C3 = $SYSMIS.

IF (RQ_K_Wis_B_M_Cat4=RQ_K_Wis_B_Re_M_Cat4) RQ_K_Wis_B_Mean_AA_C3 =1.

IF (RQ_K_Wis_B_M_Cat4>RQ_K_Wis_B_Re_M_Cat4) RQ_K_Wis_B_Mean_AA_C3 =2.

IF (RQ_K_Wis_B_M_Cat4<RQ_K_Wis_B_Re_M_Cat4) RQ_K_Wis_B_Mean_AA_C3 =3.

VARIABLE LABELS RQ_K_Wis_B_Mean_AA_C3 'Absoluut agreement voor "Wisselen zorgverlener" test-thentest - MEAN, C3' .

VALUE LABELS RQ_K_Wis_B_Mean_AA_C3

1 'test-thentest gelijk'

2 'test positiever dan hertest'

3 'test negatiever dan hertest' .

FREQUENCIES RQ_K_Wis_B_Mean_AA_C3.

NUMERIC RQ_K_Vwz_B_Mean_AA_C3 (F2).

COMPUTE RQ_K_Vwz_B_Mean_AA_C3 = $SYSMIS.

IF (RQ_K_Vwz_B_M_Cat5=RQ_K_Vwz_B_Re_M_Cat5) RQ_K_Vwz_B_Mean_AA_C3 =1.

IF (RQ_K_Vwz_B_M_Cat5>RQ_K_Vwz_B_Re_M_Cat5) RQ_K_Vwz_B_Mean_AA_C3 =2.

IF (RQ_K_Vwz_B_M_Cat5<RQ_K_Vwz_B_Re_M_Cat5) RQ_K_Vwz_B_Mean_AA_C3 =3.

VARIABLE LABELS RQ_K_Vwz_B_Mean_AA_C3 'Absoluut agreement voor "Verwijzen naar ziekenhuis" test-thentest - MEAN, C3' .

VALUE LABELS RQ_K_Vwz_B_Mean_AA_C3

1 'test-thentest gelijk'

2 'test positiever dan hertest'

3 'test negatiever dan hertest' .

FREQUENCIES RQ_K_Vwz_B_Mean_AA_C3.

NUMERIC RQ_K_Soo_B_Mean_AA_C3 (F2).

COMPUTE RQ_K_Soo_B_Mean_AA_C3 = $SYSMIS.

IF (RQ_K_Soo_B_M=RQ_K_Soo_B_RE_M) RQ_K_Soo_B_Mean_AA_C3 =1.

IF (RQ_K_Soo_B_M>RQ_K_Soo_B_RE_M) RQ_K_Soo_B_Mean_AA_C3 =2.

IF (RQ_K_Soo_B_M<RQ_K_Soo_B_RE_M) RQ_K_Soo_B_Mean_AA_C3 =3.

VARIABLE LABELS RQ_K_Soo_B_Mean_AA_C3 'Absoluut agreement voor "Keuze soort zorgverlener" test-thentest - MEAN, C3' .

VALUE LABELS RQ_K_Soo_B_Mean_AA_C3

1 'test-thentest gelijk'

2 'test positiever dan hertest'

3 'test negatiever dan hertest' .

FREQUENCIES RQ_K_Soo_B_Mean_AA_C3.

NUMERIC RQ_K_Lei_B_Mean_AA_C3 (F2).

COMPUTE RQ_K_Lei_B_Mean_AA_C3 = $SYSMIS.

IF (RQ_K_Lei_B_M=RQ_K_Lei_B_RE_M) RQ_K_Lei_B_Mean_AA_C3 =1.

IF (RQ_K_Lei_B_M>RQ_K_Lei_B_RE_M) RQ_K_Lei_B_Mean_AA_C3 =2.

IF (RQ_K_Lei_B_M<RQ_K_Lei_B_RE_M) RQ_K_Lei_B_Mean_AA_C3 =3.

VARIABLE LABELS RQ_K_Lei_B_Mean_AA_C3 'Absoluut agreement voor "Leiding zorg" test-thentest - MEAN, C3' .

VALUE LABELS RQ_K_Lei_B_Mean_AA_C3

1 'test-thentest gelijk'

2 'test positiever dan hertest'

3 'test negatiever dan hertest' .

FREQUENCIES RQ_K_Lei_B_Mean_AA_C3.

## Table 4.

Impact of experiences during pregnancy, childbirth and postnatal period, care process, interventions during childbirth, and patient reported outcomes on the total experience score during pregnancy measured after childbirth, expressed as having a negative experience, above the median score and mean score (n=462).

## Negative score

*antentale ervaring als totaal score.

LOGISTIC REGRESSION VARIABLES RQ_EvT_Neg_B_Re

/METHOD=ENTER

RQ_EvT_Neg_B RQ_EvT_Neg_C RQ_EvT_Neg_D

SD_Opl_M_C2 SD_Etm_M_C2 EE_Bew_M_C2

OV_Kza_M_C2 ZB_ZPR_M_C3 ZB_Vvw_M_C2

ZB_Bbv_M_C2 ZB_Mbv_M_C2

PB_Eub_M_C2 PB_EuM_M_C2

/CONTRAST (SD_Opl_M_C2)=Indicator(2)

/CONTRAST (SD_Etm_M_C2)=Indicator(1)

/CONTRAST (EE_Bew_M_C2)=Indicator(1)

/CONTRAST (RQ_EvT_Neg_B)=Indicator(1)

/CONTRAST (RQ_EvT_Neg_C)=Indicator(1)

/CONTRAST (RQ_EvT_Neg_D)=Indicator(1)

/CONTRAST (OV_Kza_M_C2)=Indicator(1)

/CONTRAST (ZB_ZPR_M_C3)=Indicator(1)

/CONTRAST (ZB_Vvw_M_C2)=Indicator(1)

/CONTRAST (ZB_Bbv_M_C2)=Indicator(2)

/CONTRAST (ZB_Mbv_M_C2)=Indicator(1)

/CONTRAST (PB_Eub_M_C2)=Indicator(1)

/CONTRAST (PB_EuM_M_C2)=Indicator(1)

/SAVE=PRED PGROUP COOK LEVER DFBETA ZRESID

/CLASSPLOT

/CASEWISE OUTLIER(2)

/PRINT=GOODFIT ITER(1) CI(95)

/CRITERIA=PIN(0.05) POUT(0.10) ITERATE(20) CUT(0.5).

### Median score

NUMERIC RQ_EvT_MD_B_O2 (F2).

COMPUTE RQ_EvT_MD_B_O2 = $SYSMIS.

IF (RQ_EvT_MD_B=1) RQ_EvT_MD_B_O2=2.

IF (RQ_EvT_MD_B=2) RQ_EvT_MD_B_O2=1.

VARIABLE LABELS RQ_EvT_MD_B_O2 'Boven mediaan, 2e helft zwangerschap'.

VALUE LABELS RQ_EvT_MD_B_O2

2 'positief'

1 'negatief'.

FREQUENCIES RQ_EvT_MD_B_O2.

NUMERIC RQ_EvT_MD_B_Re_O2 (F2).

COMPUTE RQ_EvT_MD_B_Re_O2 = $SYSMIS.

IF (RQ_EvT_MD_B_Re=1) RQ_EvT_MD_B_Re_O2=2.

IF (RQ_EvT_MD_B_Re=2) RQ_EvT_MD_B_Re_O2=1.

VARIABLE LABELS RQ_EvT_MD_B_Re_O2 'Boven mediaan, 2e helft zwangerschap'.

VALUE LABELS RQ_EvT_MD_B_Re_O2

2 'positief'

1 'negatief'.

FREQUENCIES RQ_EvT_MD_B_Re_O2.

NUMERIC RQ_EvT_MD_C_O2 (F2).

COMPUTE RQ_EvT_MD_C_O2 = $SYSMIS.

IF (RQ_EvT_MD_C=1) RQ_EvT_MD_C_O2=2.

IF (RQ_EvT_MD_C=2) RQ_EvT_MD_C_O2=1.

VARIABLE LABELS RQ_EvT_MD_C_O2 'Boven mediaan, bevalling'.

VALUE LABELS RQ_EvT_MD_C_O2

2 'positief'

1 'negatief'.

FREQUENCIES RQ_EvT_MD_C_O2.

NUMERIC RQ_EvT_MD_D_O2 (F2).

COMPUTE RQ_EvT_MD_D_O2 = $SYSMIS.

IF (RQ_EvT_MD_D=1) RQ_EvT_MD_D_O2=2.

IF (RQ_EvT_MD_D=2) RQ_EvT_MD_D_O2=1.

VARIABLE LABELS RQ_EvT_MD_D_O2 'Boven mediaan, kraamzorg'.

VALUE LABELS RQ_EvT_MD_D_O2

2 'positief'

1 'negatief'.

FREQUENCIES RQ_EvT_MD_D_O2.

*antentale ervaring als totaal score.

LOGISTIC REGRESSION VARIABLES RQ_EvT_MD_B_Re_O2

/METHOD=ENTER

RQ_EvT_MD_B_O2 RQ_EvT_MD_C_O2 RQ_EvT_MD_D_O2

SD_Opl_M_C2 SD_Etm_M_C2 EE_Bew_M_C2

OV_Kza_M_C2 ZB_ZPR_M_C3 ZB_Vvw_M_C2

ZB_Bbv_M_C2 ZB_Mbv_M_C2

PB_Eub_M_C2 PB_EuM_M_C2

/CONTRAST (SD_Opl_M_C2)=Indicator(2)

/CONTRAST (SD_Etm_M_C2)=Indicator(1)

/CONTRAST (EE_Bew_M_C2)=Indicator(1)

/CONTRAST (RQ_EvT_MD_B_O2)=Indicator(1)

/CONTRAST (RQ_EvT_MD_C_O2)=Indicator(1)

/CONTRAST (RQ_EvT_MD_D_O2)=Indicator(1)

/CONTRAST (OV_Kza_M_C2)=Indicator(1)

/CONTRAST (ZB_ZPR_M_C3)=Indicator(1)

/CONTRAST (ZB_Vvw_M_C2)=Indicator(1)

/CONTRAST (ZB_Bbv_M_C2)=Indicator(2)

/CONTRAST (ZB_Mbv_M_C2)=Indicator(1)

/CONTRAST (PB_Eub_M_C2)=Indicator(1)

/CONTRAST (PB_EuM_M_C2)=Indicator(1)

/SAVE=PRED PGROUP COOK LEVER DFBETA ZRESID

/CLASSPLOT

/CASEWISE OUTLIER(2)

/PRINT=GOODFIT ITER(1) CI(95)

/CRITERIA=PIN(0.05) POUT(0.10) ITERATE(20) CUT(0.5).

### Mean score

**Leeftijdd

**zorgproces.

DO IF (1-MISSING(ZB_ZPR_M_C3)).

RECODE ZB_ZPR_M_C3 (2=1) (ELSE=0) INTO ZB_VTP.

RECODE ZB_ZPR_M_C3 (3=1) (ELSE=0) INTO ZB_VTB.

END IF.

VARIABLE LABELS ZB_VTP 'verwezen tijdens zwangerschap'.

VARIABLE LABELS ZB_VTB 'verwezen tijdens bevalling zonder spoed'.

EXECUTE.

*STAP 3A: MULTIPLE REGRESSIE ANALYSE - FORCED ENTRY.

*antenatale ervaring: totale score.

REGRESSION

/DESCRIPTIVES MEAN STDDEV CORR SIG N

/MISSING LISTWISE

/STATISTICS COEFF OUTS CI(95) R ANOVA COLLIN TOL CHANGE ZPP

/CRITERIA=PIN(.05) POUT(.10)

/NOORIGIN

/DEPENDENT RQ_EvT_B_Re

/METHOD=ENTER RQ_EvT_B

/METHOD=ENTER RQ_EvT_C

/METHOD=ENTER RQ_EvT_D

/METHOD=ENTER OV_Kza_M_C2

/METHOD=ENTER ZB_VTP ZB_VTB

/METHOD=ENTER ZB_Vvw_M_C2

/METHOD=ENTER ZB_Mbv_M_C2

/METHOD=ENTER PB_Eub_M_C2

/METHOD=ENTER PB_Eum_M_C2

/METHOD=ENTER SD_Opl_M_C2

/METHOD=ENTER SD_Etm_M_C2

/METHOD=ENTER EE_Bew_M_C2

/METHOD=ENTER ZB_Bbv_M_C2

/RESIDUALS DURBIN

/CASEWISE PLOT(ZRESID) OUTLIERS(2.5).
